# Exclusive Hydroformylation of Terminal Olefins: A Phosphorus‐Free Ruthenium System for the Selective Functionalization of Complex Mixtures

**DOI:** 10.1002/anie.3316419

**Published:** 2026-07-23

**Authors:** Mohamed Niyaz Vellala Syed Ali, Ida Ziccarelli, Dilver Peña Fuentes, Christoph Kubis, Robert Franke, Ralf Jackstell, Matthias Beller

**Affiliations:** ^1^ Department of Applied Homogeneous Catalysis Leibniz‐Institut für Katalyse e. V. Albert‐Einstein‐Straße 29a Rostock Germany; ^2^ Laboratory of Industrial and Synthetic Organic Chemistry (LISOC) Department of Chemistry and Chemical Technologies University of Calabria Arcavacata di Rende Cosenza Italy; ^3^ Evonik Oxeno GmbH Paul‐Baumann‐Straße. 1 Marl Germany; ^4^ Lehrstuhl Für Theoretische Chemie Ruhr‐Universität Bochum Bochum Germany

**Keywords:** chemo‐ and regioselective hydroformylation, DBU as a ligand, phosphor‐free ruthenium catalyst, ruthenium‐hydride intermediate, selective terminal olefin functionalization

## Abstract

We report a paradigm shift in ruthenium‐catalyzed hydroformylation using a remarkably simple, phosphorus‐free system based on ruthenium carbonyl complexes and DBU (1,8‐diazabicyclo[5.4.0]undec‐7‐ene). The optimal catalytic system operates under mild conditions and exhibits an unprecedented “terminal‐only” selectivity. It quantitatively discriminates between terminal and internal or multi‐substituted double bonds, which has been a long‐standing challenge in oxo synthesis. Traditional systems often trigger the isomerization of internal olefins, but our catalyst leaves them untouched, even within complex industrial feedstocks. Comprehensive mechanistic investigations, including in situ IR spectroscopy and high‐pressure VT NMR spectroscopy, identify the tetranuclear ruthenium‐hydride cluster [DBUH]_2_^+^[H_2_Ru_4_(CO)_12_]^2−^ as the pivotal active species. Isolating this cluster and successfully reapplying it confirms its role as the catalytic resting state. Combining low‐cost, air‐stable components with exceptional chemo‐ and regioselectivity provides this system with a robust, economically superior alternative to precious rhodium‐phosphine catalysts for academic synthesis and large‐scale industrial applications .

## Graphical abstract

Development of a phosphorus‐free Ru/DBU catalyst enabling highly chemo‐ and regioselective hydroformylation of terminal olefins, sparing internal double bonds. Optimization of reaction parameters, ligand effects, and substrate scope including industrial feedstocks was realized. Mechanistic and spectroscopic insights reveal the tetranuclear anionic ruthenium cluster [DBUH]_2_^+^[H_2_Ru_4_(CO)_12_]^2−^ as key intermediate during the reaction. A simple, economical, scalable catalyst platform with broad synthetic and industrial relevance is presented.

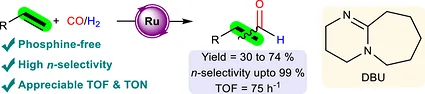

## 1 Introduction

**SCHEME 1:**
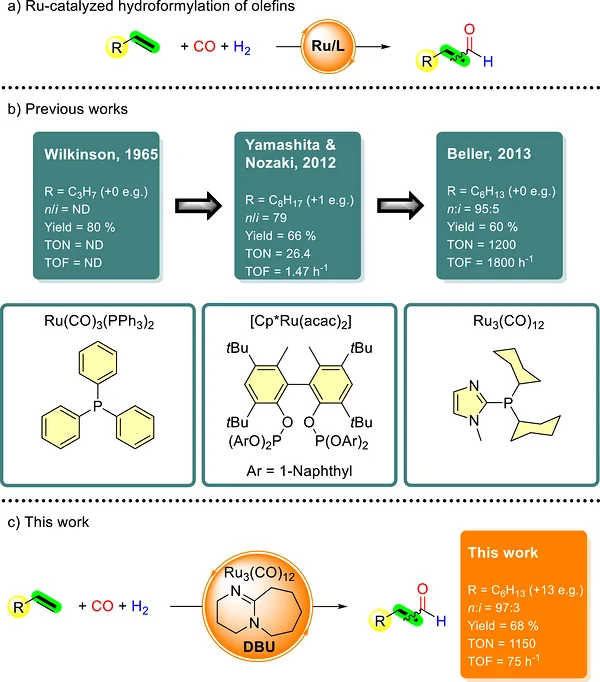
Development of ruthenium catalysts for hydroformylation of olefins (reported examples and this work).

Hydroformylation (oxo synthesis) is an important industrial reaction to synthesize aldehydes, where olefins react with synthesis gas (a mixture of H_2_/CO) in the presence of a suitable catalyst (Scheme 1a) [1, 2, 3, 4, 5, 6, 7, 8, 9, 10]. The aldehydes produced play a role as key intermediates in a wide range of industrial applications, including plasticizers for PVC, perfumes, agricultural chemicals, pharmaceuticals, disinfectants, and dyes. Worldwide, nearly 10 million tons of oxo products are produced annually [11, 12, 13]. Despite it being nearly a century since its discovery by Otto Roelen, who was a pioneer of the industrial homogeneous catalysis [14, 15], significant opportunities remain open worldwide in research on novel catalysts and methods to further optimize the process.

In general, hydroformylation reactions demand a peculiar selectivity, both in regioselectivity and chemoselectivity. Regioselectivity is particularly challenging when hydroformylating terminal olefins, because the reaction can produce mixtures of branched (*i*) and linear (*n*) aldehydes, depending on whether the inner or terminal carbon atom of the olefin is formylated. In addition, the double bond of the olefin can also isomerize, which can lead to an increased proportion of branched products. Chemoselectivity is also equally important, as the olefin substrates and/or the resulting aldehyde products can also further undergo hydrogenation to form alkanes or alcohols, respectively mainly depending on the catalyst and reaction conditions. For large‐scale industrial applications, linear aldehydes are predominantly targeted [16, 17].

Since its discovery, hydroformylation has been catalyzed by a variety of transition metal complexes having the general form H‐M(CO)_n_L_m_ (L = ligand, typically phosphines or phosphites) as an active species. Initially, cobalt catalysts were employed [18, 19, 20], after the 1970s rhodium complexes [21, 22, 23, 24, 25, 26, 27, 28, 29] became dominating due to the fact of their excellent activity and selectivity. Even then, considerable efforts have been made to develop effective catalysts based on other metals, and in general, these alternatives generally exhibit lower efficiency compared to the rhodium catalysts. The transition metal's catalytic activity for hydroformylation follows the trend: Rh > Co > Ir > Ru > Os > Pt > Pd > Fe > Ni [8].

Nevertheless, recent studies, including our own work, have demonstrated that efficiency can be significantly improved by altering reaction conditions or using specific ligands that allow the expensive rhodium system to be replaced with more affordable metals such as ruthenium [30, 31, 32, 33, 34, 35, 36, 37, 38]. In this context, due to their availability and lower cost, ruthenium (Ru) complexes have emerged and are emerging as a promising alternative for hydroformylation catalysts. However, controlling the regioselectivity and chemoselectivity with Ru catalysts remains a significant challenge [39]. It is also worth noting that olefin isomerization arising from the reversible hydrometallation/β‐hydride elimination activity of ruthenium hydride intermediates is a recognized side reaction in ruthenium‐based hydroformylation [40], and even rhodium–phosphine systems are not fully immune to this, particularly at elevated temperatures or with less hindered substrates [8].

A literature study reveals that the first ruthenium‐catalyzed hydroformylation was described in 1965 by Evans, Osborn, Jardine, and Wilkinson [41]. Despite this early discovery, Ru‐based systems initially lagged a bit behind rhodium systems mainly in terms of efficiency until Süss‐Fink et al. [42], demonstrated that trinuclear ruthenium carbonyl clusters, such as [HRu_3_(CO)_11_]^−^ could catalyze the hydroformylation of ethylene and propene under mild conditions with high chemoselectivity. In parallel, Knifton et al. [43] introduced the so‐called “ruthenium melt” systems, where ruthenium complexes were dispersed in low‐melting phosphonium salts, and later combined with chelating ligands that enabled the conversion of internal olefins to linear alcohols.

Then, more recently, Haukka et al. [44, 45] conducted a series of investigations both experimentally and theoretically to understand the steric and electronic effect of the ligands used and their correlation with the catalytic performance. To further address the persistent challenges in selectivity and substrate scope, Nozaki et al. [46, 47] demonstrated in 2012 that [Ru^II^(Cp*)] complexes in combination with bidentate ligands enable the hydroformylation of propene and 1‐decene in high regioselectivity under comparatively milder conditions, but this was limited by moderate activity and substrate generality. Shortly thereafter, we [48, 49] showed that in situ generated Ru complexes bearing an electron‐rich imidazole‐substituted dialkylphosphines achieve both regioselectivity and excellent catalytic activity in the hydroformylation of 1‐octene under mild conditions as well (Scheme 1b).

Obviously, as far as our understanding goes, in hydroformylation reactions, both the metal center and the added ligands play a crucial role in controlling the activity and selectivity, and traditionally, this has been achieved using phosphines or phosphites as ligands of choice [50, 51, 52, 53, 54, 55, 56, 57]. However, these ligands may be comparably costly to the noble metals themselves and frequently necessitate specialized handling procedures, thereby imposing significant cost burdens upon large‐scale industrial implementation in terms of both capital expenditures (CAPEX) and operational expenditures (OPEX). Being motivated by the goal of designing catalysts based on “cheap metals and cheap ligands,” we focused our attention on exploring phosphorus (P)‐free ruthenium (Ru) catalysts for carbonylation processes. Here, we demonstrated that the combination of Ru_3_(CO)_12_ with catalytic amounts of amidines, such as DBU and DBN, offers a viable alternative to expensive rhodium complexes with air‐sensitive phosphines. This catalyst system is not only more cost‐effective but also easier to handle while maintaining the highly chemo‐ and regioselective hydroformylation reactions (Scheme 1c).

## 2 Results and Discussion

### 2.1 Catalyst Development and Reaction Parameter Optimizations

At the beginning of this research project, we have selected 1‐octene **1a** as a model system to develop a P‐free Ru catalyst for hydroformylation. Our initial catalytic experiments were performed using Ru‐, Rh‐, and Co‐based carbonyl precursors under identical conditions, employing a mixture of CO/H_2_ (1:3) totaling to 40 bar pressure and 120°C in a solvent mixture of NMP (*N*‐methyl‐2‐pyrrolidone) and H_2_O. Under these conditions, Ru_3_(CO)_12_ alone showed moderate hydroformylation activity (Table 1, entry 1), affording 29% C_9_‐aldehydes with an unexpectedly high *n*/*i* selectivity (93:7) compared to the Rh and Co catalysts (Table S1). The addition of DBU resulted in an improvement, yielding 53% aldehydes with excellent regioselectivity (97:3), along with some olefin isomerization happening (Table 1, entry 11). In contrast, Rh(acac)(CO)_2_ provided the highest aldehyde yield (85%) without DBU (Table S1, entry 2) but showed poor linear selectivity (53:47). Co_2_(CO)_8_ was totally inactive and performed only double‐bond migration (Table S1, entry 3). Interestingly, the addition of DBU completely disturbs the reactivity of both Rh and Co catalysts (Table S1, entries 4 and 5), stating that DBU acts as a promoter uniquely for ruthenium‐based systems. These results indicate that Ru/DBU operates via a unique catalytic pathway, showing high activity and excellent *n*‐selectivity, and this cannot be achieved with conventional Rh‐ or Co‐based systems under identical conditions.

**TABLE 1:** Ru‐catalyzed hydroformylation of 1‐octene: Variation of nitrogen‐based ligands.^a^ ^.^

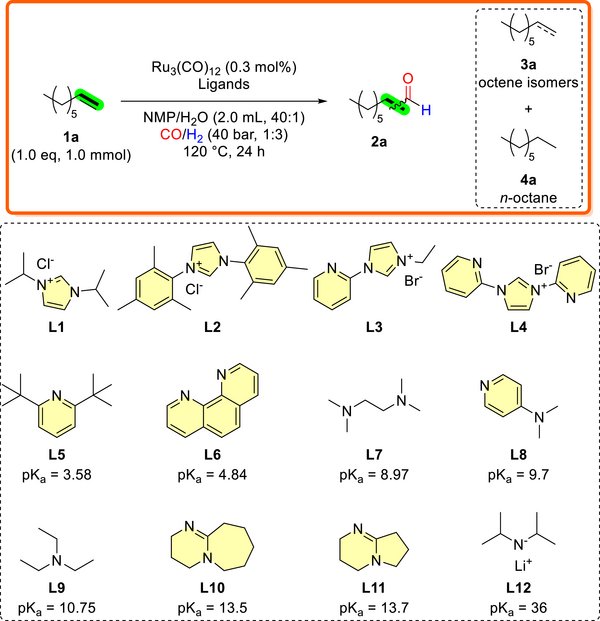					
Entry	Ligands	Conversion of 1a [%]	Yield [%]		
			2a (*n*:*i*)	3a	4a
1	—	92	29 (93:7)	55	8
2	L1	90	54 (85:15)	31	5
3	L2	90	47 (85:15)	38	5
4	L3	79	39 (94:6)	36	4
5	L4	51	24 (85:15)	23	4
6	L5	91	31 (95:5)	56	4
7	L6	85	41 (96:4)	41	3
8	L7	89	44 (99:1)	42	3
9	L8	87	46 (99:1)	38	3
10	L9	90	46 (99:1)	41	3
11	L10	95	53 (97:3)	38	4
12	L11	97	49 (97:3)	42	6
13	L12	95	42 (97:3)	49	4
14^b^	L10 + LiCl	94	49 (97:3)	41	4
15^c^	L10 + LiCl	94	47 (97:3)	44	3
16^d^	L10 + LiCl	94	41 (97:3)	49	4

^a^ Reaction conditions: **1a** (1.0 eq, 1.0 mmol), Ru_3_(CO)_12_ (0.3 mol%), added ligands (4.0 mol%), NMP/H_2_O (2.0 mL, 40:1), CO/H_2_ (40 bar, 1:3), 120°C, 24 h reaction time.

^b^ LiCl (5.0 mol%).

^c^ LiCl (10.0 mol%).

^d^ LiCl (20.0 mol%). The ratio of linear to all branched products = *n*:*i*. Yields and *n*:*i* are determined by GC analysis using isooctane (57.0 mg, 0.5 mmol) as internal standard.

Next, we started optimizing the ligands. First, we tried performing it in the presence of various *N*‐heterocyclic carbene (NHC) precursors. As shown in Table 1, entries 2–5, the addition of 4 mol% imidazolylidene carbenes led to an increase in the aldehyde yields (39%–54%), but the regioselectivity decreased slightly. Notably, the addition of 1,10‐phenanthroline, a ligand that is well known to stabilize Ru complexes [58, 59], further improved the yield and selectivity compared to Ru_3_(CO)_12_ alone (Table 1, entry 7 vs. entry 1). This encouraged us to expand the optimization to other nitrogen‐based ligands. As illustrated in Table 1, entries 6–13, the pK_a_ value of the nitrogen‐based ligand appeared to influence both the yield of the aldehyde (**2a**) and the selectivity.

In general, we observed that an increase in the pKa value resulted in higher yields of **2a**, and we noticed the best result for 1,8‐diazabicyclo[5.4.0]undec‐7‐ene (DBU) (Table 1, entry 11). Additionally, a few reactions were also conducted with inorganic bases (Table S1, entries 6 and 7), but this led to a significant decrease in both yield and regioselectivity of **2a**. It is also good to observe that the regioselectivity for the industrially desired linear isomer remained very high (95:5–99:1) for all nitrogen‐based ligands. The use of DBU particularly showed an improvement in both the regioselectivity and yield of **2a** by surpassing the performance of traditional Ru/phosphine catalysts under similar conditions. While the general trend of increasing aldehyde yield with increasing ligand pKa value points toward basicity as a key factor in promoting the formation of the active anionic cluster, we acknowledge that steric contributions from the nitrogen‐based ligands cannot be fully excluded based on the current data, and a more systematic investigation of this aspect is planned for future work.

In addition to the nitrogen‐based ligands, the influence of other additives was also tested. Mainly, Sasaki et al. [60] then later the Haukka et al. [61] as well as our group [62] have previously reported a positive effect of LiCl in Ru‐catalyzed carbonylation of olefins. We also observed the same effect in our model reaction too (Table S2, entries 1–4), with the yield of product **2a** increasing from 29% in the absence of LiCl to 59% when 10.0 mol% of lithium chloride was added. However, while the yield improved, both the chemo‐ and regioselectivity were lower compared to the system with DBU. We further optimized the LiCl concentration (Table S3) and found that the best yield of **2a** was observed at 10.0 mol% of LiCl (Table S3, entry 3). Although the presence of LiCl improved the yield, it also reduced the selectivity of the reaction. When DBU was used in combination with LiCl, regioselectivity was good, but a gradual decrease in the yield of **2a** was observed (Table 1, entries 14–16). By this we understood that the use of cyclic amidines such as DBU or DBN allowed for the synthesis of the desired aldehyde **2a** in yields of up to 53% even without the addition of LiCl.

**FIGURE 1:**
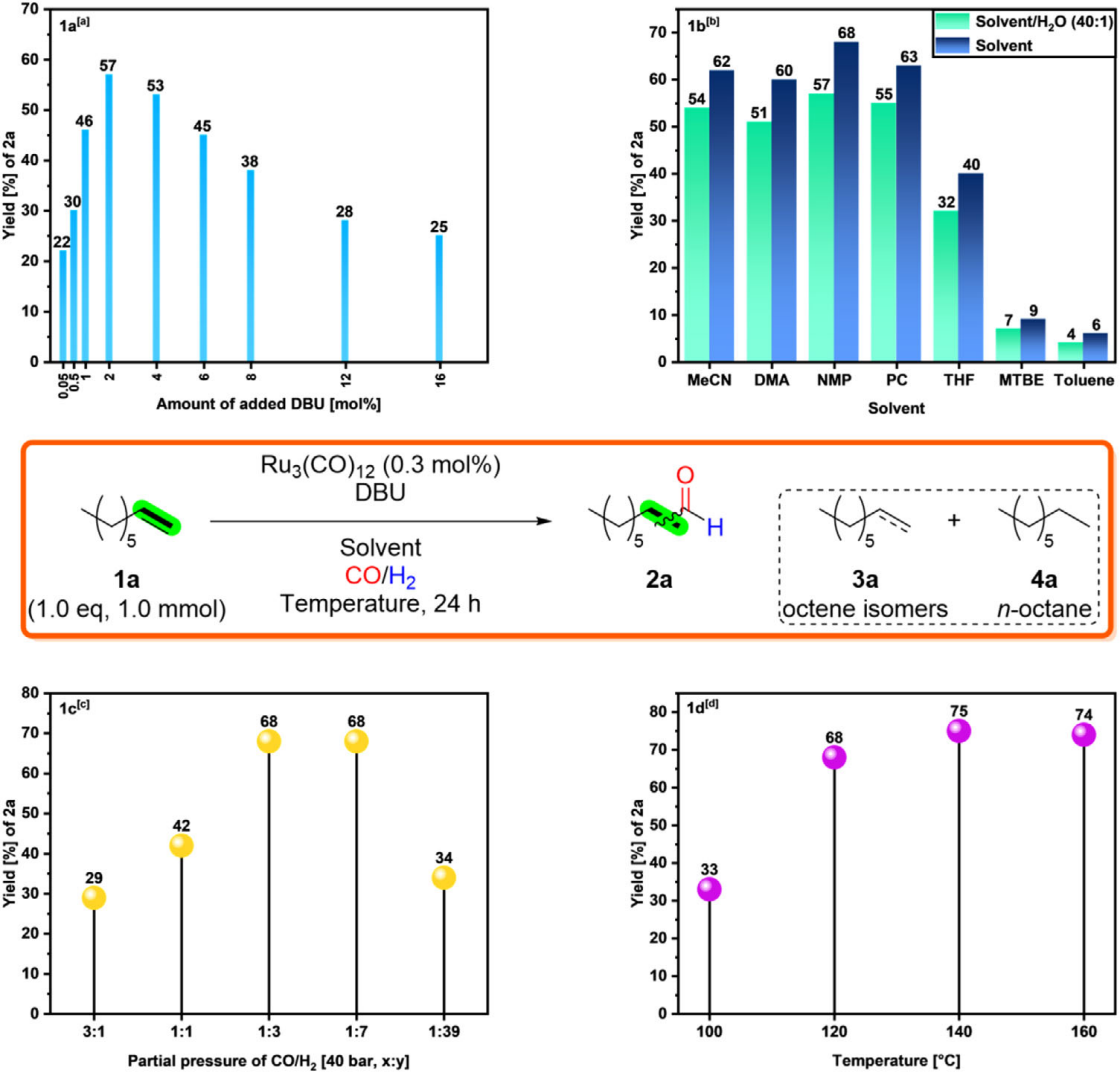
Study of critical reaction parameters: Amount of DBU, solvent, partial pressure, and temperature. (a) Reaction conditions: **1a** (1.0 eq, 1.0 mmol), Ru_3_(CO)_12_ (0.3 mol%), DBU (mol%), NMP/H_2_O (2.0 mL, 40:1), CO/H_2_ (40 bar, 1:3), 120°C, 24 h reaction time. (b) Reaction conditions: **1a** (1.0 eq, 1.0 mmol), Ru_3_(CO)_12_ (0.3 mol%), DBU (2.0 mol%), Solvent (2.0 mL), CO/H_2_ (40 bar, 1:3), 120°C, 24 h reaction time. (c) Reaction conditions: **1a** (1.0 eq, 1.0 mmol), Ru_3_(CO)_12_ (0.3 mol%), DBU (2.0 mol%), NMP (2.0 mL), CO/H_2_ (40 bar, x:y), 120°C, 24 h reaction time. (d) Reaction conditions: **1a** (1.0 eq, 1.0 mmol), Ru_3_(CO)_12_ (0.3 mol%), DBU (2.0 mol%), NMP (2.0 mL), CO/H_2_ (40 bar, 1:7), Temperature (°C), 24 h reaction time. The ratio of linear to all branched products = *n*:*i*. Yields and *n*:*i* are determined by GC analysis using isooctane (57.0 mg, 0.5 mmol) as internal standard.

To further improve the performance of our system, we tried varying the key reaction parameters, such as the amount of DBU, solvent, partial pressures of CO and H_2_, and temperature (Figure 1). The catalytic system showed sensitivity toward the amount of DBU used, while the regioselectivity remained unaffected, as illustrated in Figure 1a (Table S4). The optimal amount of DBU was found to be 2.0 mol%, as it gave the highest yield of **2a** at 57%. We also tested various polar and non‐polar solvents, both with and without the addition of water. As shown in Figure 1b (Table S5), we observed that the addition of water slightly reduces catalytic activity. The best results were observed in polar aprotic solvents, such as dimethylacetamide (DMA), propylene carbonate (PC), *N*‐methyl‐2‐pyrrolidone (NMP), and acetonitrile, all of which yielded in the same range. In contrast, non‐polar solvents like THF, MTBE, and toluene led to lower yields of the aldehyde.

Then the effect of partial pressures of CO and H_2_ was also tested, as illustrated in Figure 1c (Table S6). While varying the ratios of CO and H_2_, once again the system did not show any change in the regioselectivity, but the yield was changed. High yields of **2a** were achieved at a CO:H_2_ ratio of 1:7, and deviations from this ratio led to a decrease in aldehyde yield. Finally, we examined the impact of temperature (Figure 1d and Table S7). Reactions were performed at 100°C, 120°C, 140°C, and 160°C. As expected, the reaction at 100°C showed low conversion and so did the yield. Notably, the yields at 120°C, 140°C, and 160°C were nearly in the same range, indicating that our system remains stable even at elevated temperatures. Now, considering energy efficiency, we selected 120°C as the optimal reaction temperature.

### 2.2 Experimental Investigations of the Reaction Mechanism and Spectroscopic Studies

To understand the catalytic efficiency of our system, we recorded the turnover number (TON) and turnover frequency (TOF) by hourly sampling of the reaction mixture, and in parallel, the gas consumption was also monitored (Table S8 and Figure S1). Based on the kinetic data (Table S8 and Figure S1), the catalytic system demonstrates high stability throughout the reaction course, with the reaction rate tapering off only after near‐complete substrate conversion (∼98%) is reached at 22–26 h (TON = 1150). While the plateau primarily reflects substrate depletion, potential long‐term deactivation pathways typical for homogeneous cluster catalysis, such as thermal decomposition or aggregation under prolonged reaction times, cannot be fully ruled out at this stage, as formal recycling experiments were not conducted. The catalyst TON was calculated based on the maximum yield achieved at the conversion plateau, while TOF was calculated from the yield observed within the first 2 h. The 2‐h window was selected based on the approximately linear increase in product yield observed during this early stage of the reaction (Table S8), before substrate depletion and product inhibition begin to influence the rate representing the most reliable timeframe for estimating intrinsic catalytic activity. Throughout the experiment, the ruthenium concentration was maintained at 0.3 mM. Under these conditions, the TON was 1150, and the TOF was calculated to be 75 h^−1^ over the 2‐h period (Table S9).

**FIGURE 2:**
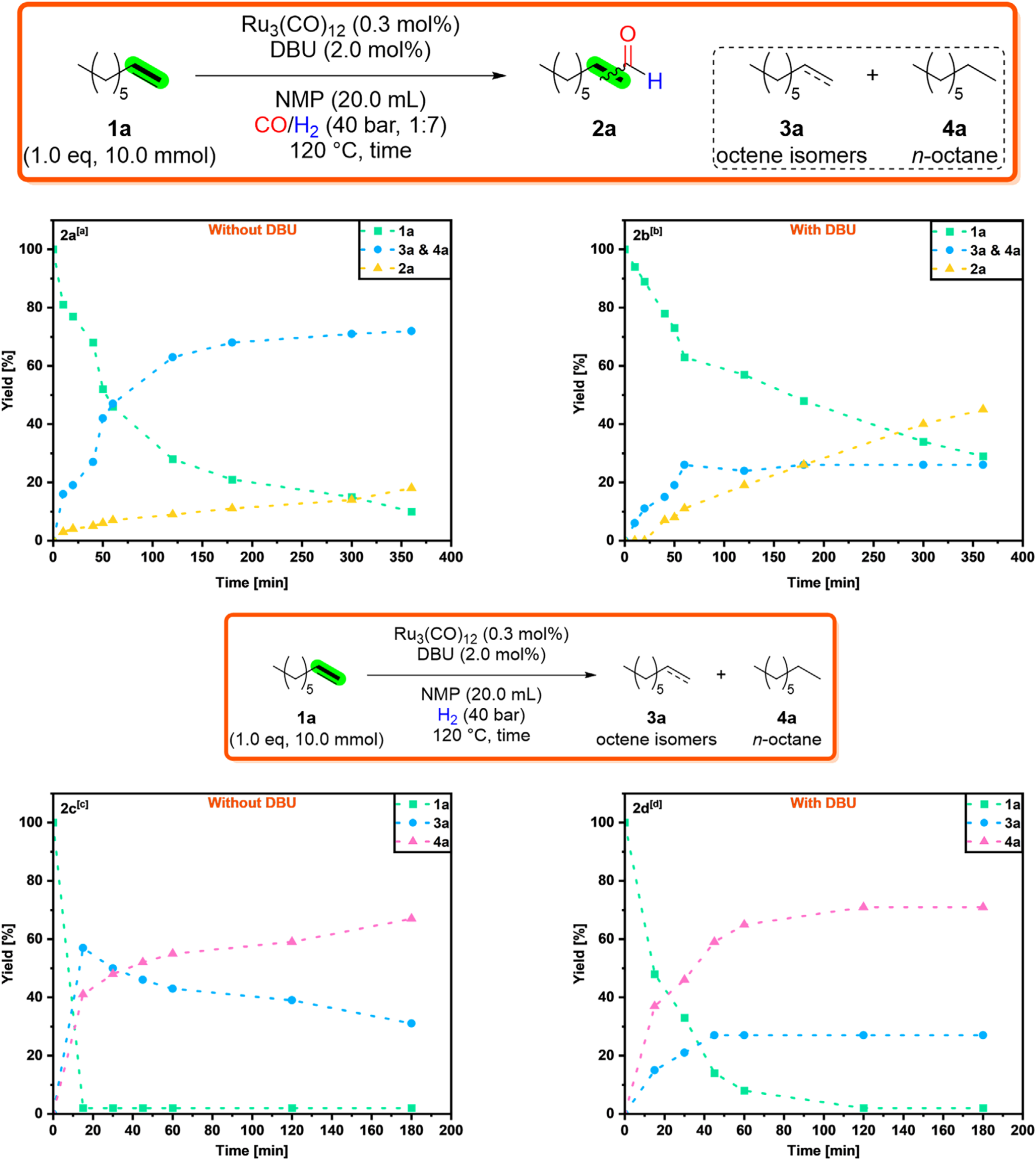
Kinetic profiles of the hydroformylation and hydrogenation reactions: Comparative analysis with and without DBU. (a) Reaction conditions: **1a** (1.0 eq, 10.0 mmol), Ru_3_(CO)_12_ (0.3 mol%), NMP (20.0 mL), CO/H_2_ (40 bar, 1:7), 120°C, time (min). (b) Reaction conditions: **1a** (1.0 eq, 10.0 mmol), Ru_3_(CO)_12_ (0.3 mol%), DBU (2.0 mol%), NMP (20.0 mL), CO/H_2_ (40 bar, 1:7), 120°C, time (min). (c) Reaction conditions: **1a** (1.0 eq, 10.0 mmol), Ru_3_(CO)_12_ (0.3 mol%), NMP (20.0 mL), H_2_ (40 bar), 120°C, time (min). (d) Reaction conditions: **1a** (1.0 eq, 10.0 mmol), Ru_3_(CO)_12_ (0.3 mol%), DBU (2.0 mol%), NMP (20.0 mL), H_2_ (40 bar), 120°C, time (min). The ratio of linear to all branched products = *n*:*i*. Yields and *n*:*i* are determined by GC analysis using isooctane (57.0 mg, 0.5 mmol) as internal standard.

Following the initial kinetic profile, four additional kinetic control experiments were also conducted to further investigate the role of DBU in the reaction system (Figure 2). In the first part, hydroformylation reactions were performed in both the absence (Figure 2a and Table S10) and presence (Figure 2b and Table S11) of DBU, and the corresponding product yields were recorded periodically. From the kinetic curves we observed that in the presence of DBU, the formation of octene isomers increased steadily at first, then suddenly stopped and remained constant for the remainder of the reaction.

To further confirm this unusual behavior, secondly, a control hydrogenation experiment was also carried out under similar conditions (Figure 2c,d; Tables S12 and S13). Interestingly, we observed a similar pattern where the generation of octene isomers increased initially, but then suddenly stopped, showing complete inhibition of further isomerization. These findings suggest that the presence of DBU suppresses the isomerization of olefins, thereby promoting the gradual consumption of the initial terminal olefins. The kinetic profiles also showed an induction period of around 30 min in reactions containing DBU, as shown in Figure 2b, and this may be due to the pre‐formation of the active Ru complex. To confirm this, we also performed a pre‐activation experiment (Table S14 and Figure S2) in which the catalytic system was allowed to react under standard conditions for 30 min before adding **1a**. After the addition of **1a**, the reaction was then monitored. By performing this, the induction period was effectively eliminated, and the initial reaction rate was boosted compared to the non‐activated system.

With the aim of gaining some insights into the pre‐formation of the active Ru‐complex and to elucidate the structure of the molecular complexes involved in the phosphorus‐free ruthenium‐catalyzed hydroformylation, we performed some in situ infrared as well as high‐pressure variable temperature NMR (VT‐HP NMR) experiments. As the ruthenium‐catalyzed hydroformylation of terminal olefins proceeded well in acetonitrile in terms of conversion and selectivity, and because of the availability of this deuterated solvent and its suitability for infrared measurements, we selected it for the NMR and IR spectroscopic studies. Preliminary ex situ IR measurements at 25°C of solutions from the starting materials DBU ($\tilde{\nu }$ = 1615 cm^−1^) and [Ru_3_(CO)_12_] ($\tilde{\nu }$ = 2014, 2029, and 2062 cm^−1^) in acetonitrile under argon were conducted. The infrared spectrum after the treatment of [Ru_3_(CO)_12_] with DBU in acetonitrile for 4 h under argon showed significant changes in the band positions and pattern within the region of terminal carbonyl ligands ($\tilde{\nu }$ = 1955, 1989, 2017, and 2075 cm^−1^). Additional bands at $\tilde{\nu }$ = 1714, 1757, and 1789 cm^−1^ were observed, which might be assigned to *μ*‐bridged carbonyl ligands of an anionic ruthenium carbonyl complex or a respective complex mixture (Figure S3). The band at $\tilde{\nu }$ = 1650 cm^−1^ likely stems from protonated [DBUH]^+^[63]. According to the literature, the spectroscopic features with respect to the band positions and intensity pattern are in part in good agreement with the formation of the cluster anion [HRu_3_(CO)_11_]^−^ [64, 65].

**FIGURE 3:**
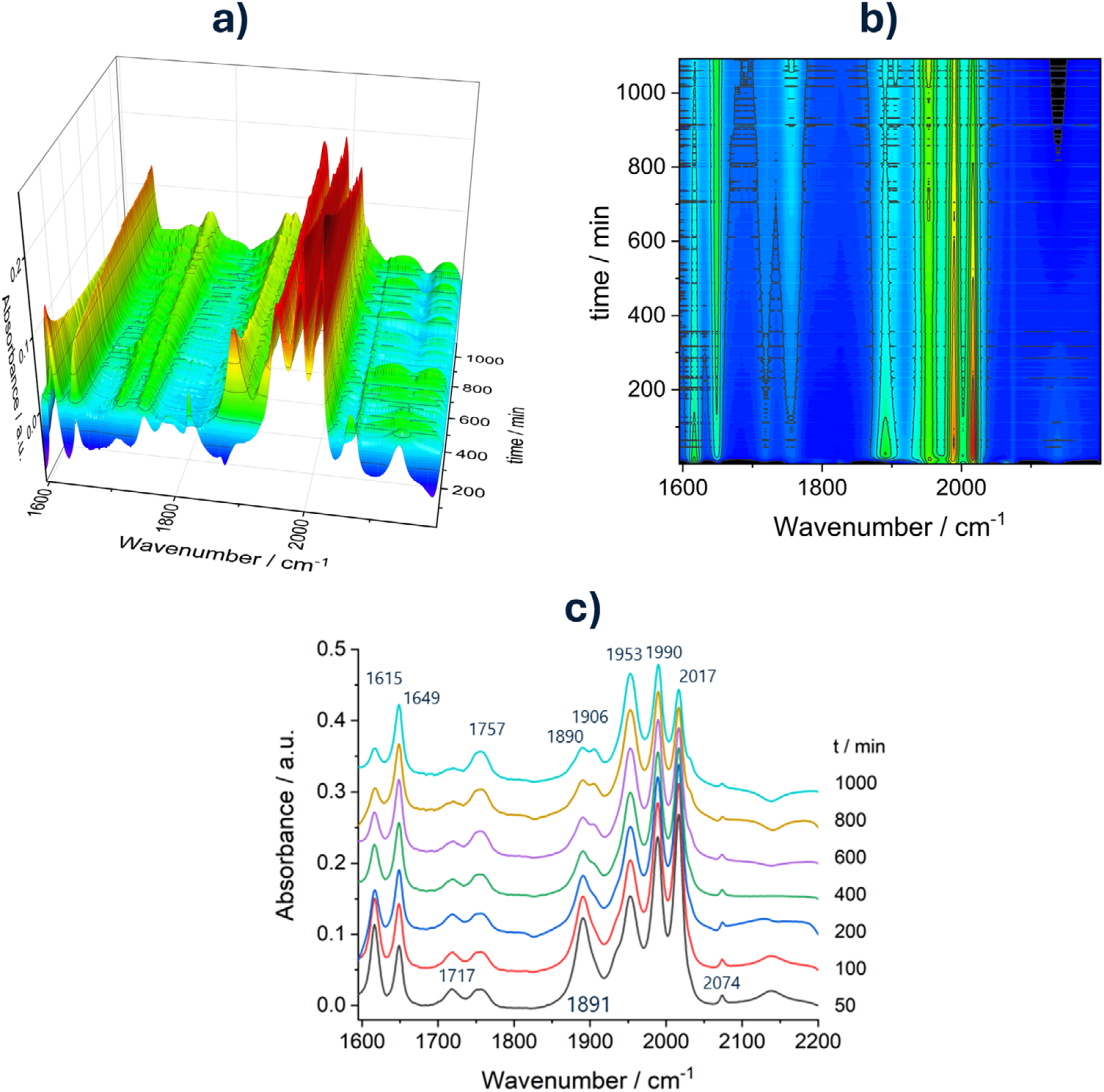
In situ infrared spectroscopic data (3‐D plot (a), contour plot (b), selected spectra as 2‐D plot (c)) collected during the treatment of [Ru_3_(CO)_12_]/DBU at reaction conditions for 1000 min: ϑ = 120°C, p(H2) = 35 bar, p(CO) = 5 bar.

Under reaction conditions at 120°C in the presence of hydrogen (p(H_2_) = 35 bar) and carbon monoxide (p(CO) = 5 bar), besides the band features for [HRu_3_(CO)_11_]^−^ an additional band at 1891 cm^−1^ is observable, which was reported in the literature but not attributed to a molecular structure. Within the time span of 16 h (1000 min) the relative intensity pattern of the bands ($\tilde{\nu }$ = 1953, 1990, 2017, and 2074 cm^−1^) in the region of terminal carbonyl ligands changed, indicating the transformation of the initially formed [HRu_3_(CO)_11_]^−^. The intensity of the band at $\tilde{\nu }$ = 1891 cm^−1^ decreased noticeably, revealing another overlapping band at 1906 cm^−1^ (Figure 3). The decrease of the band at $\tilde{\nu }$ = 1615 cm^−1^ together with the increase of the band at $\tilde{\nu }$ = 1649 cm^−1^ showed that most of the DBU was transformed into its protonated form [DBUH]^+^. Reaching this point, we continued performing an isotopic exchange of hydrogen to deuterium, which was conducted to clarify whether an H/D‐CO‐trans orientation exists in the molecular structure of the complex(es). No shift of respective bands was observed; thus, a H/D‐CO‐trans orientation is not expected. The pattern of the bands at $\tilde{\nu }$ = 1757, 1889, 1906, 1952, 1990, 2017, and 2032 cm^−1^ after reaching a nearly quasi‐stationary state kept relatively constant over 20 h with only slight changes in band intensities. It is important to note that the band of protonated DBU ([DBUH]^+^) shifted from $\tilde{\nu }$ = 1650 to $\tilde{\nu }$ = 1639 cm^−1^ after isotopic exchange to deuterium (Figure S4).

**FIGURE 4:**
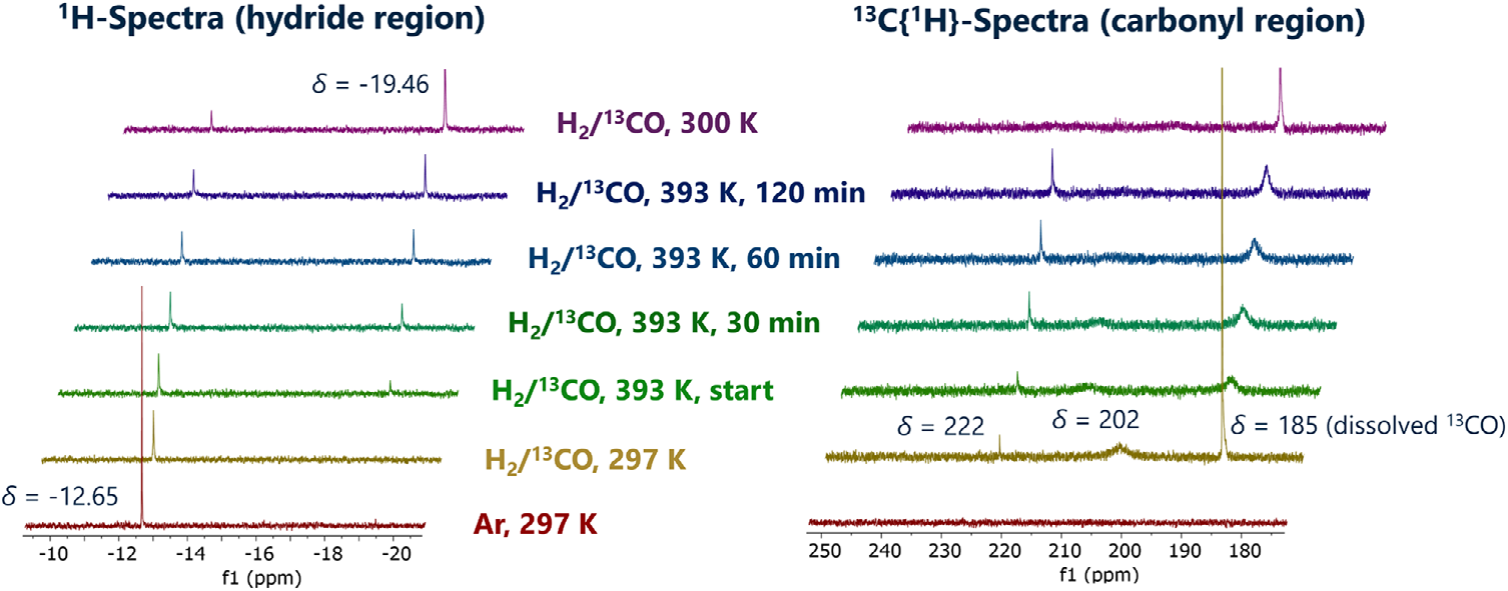
In situ ^1^H‐ and ^13^C{^1^H} HP‐VT NMR spectra for the reaction of [Ru_3_(CO)_12_] with DBU under H_2_/^13^CO. Reaction conditions: [Ru] = 12 mM, [DBU] = 15.6 mM (1.3 eq.), solvent = CD_3_CN, 𝜗 = 393 K (120°C), p(^13^CO) = 5 bar, p(H_2_) = 35 bar, reaction time = 2 h.

NMR experiments were also carried out to investigate the preformation of the active Ru complex. A solution of [Ru_3_(CO)_12_] (4 mM) and DBU (16 mM) in a dry and degassed acetonitrile‐*d*_3_ was transferred to a sapphire high‐pressure NMR cell under argon and then pressurized with 5 bar ^13^CO and 35 bar H_2_. Since the reaction could not be monitored for a long period of time in the spectrometer, the remaining solution was transferred to a 10 mL Swagelok reactor, where the reaction proceeded for 15 h. The reaction output was evaluated afterwards by ex situ NMR. The control ^1^H‐NMR spectrum of the reaction mixture under argon showed the immediate formation of a hydride complex with a resonance at *δ* = −12.65 ppm (Figure 4) in a very low concentration (about 7%) in comparison with the concentration of DBU. This signal agrees with the formation of the cluster anion [HRu_3_(CO)_11_]^−^, with [DBUH]^+^ as the cation. The previously reported [NEt_4_]^+^[HRu_3_(CO)_11_]^−^ complex showed an almost identical resonance for the hydride signal at *δ* = −12.76 ppm in acetonitrile‐*d*_3_ [65]. Since [DBUH]^+^[HRu_3_(CO)_11_]^−^ is generated under argon and dry conditions, it seems reasonable that the barely acidic acetonitrile acts as the H‐source. Upon addition of H_2_ and CO at room temperature, followed by heating up to 120°C, [DBUH]^+^[HRu_3_(CO)_11_]^−^ remained as the main hydride species, but a new very low intensity signal appeared in the hydride region of the ^1^H‐NMR spectrum with a resonance at *δ* = −19.46 ppm at 120°C. The intensity of the new signal increases readily with the time to become the main hydride species after 2 h. These results agree with the kinetic profiles observed for the hydroformylation reaction without and with pre‐activation of the catalyst (Figures 2b and S2). At the beginning of the reaction, mainly isomerization of 1‐octene is observed with very low aldehyde product formation. This effect could be attributed to the cluster anion [HRu_3_(CO)_11_]^−^ with reported activity in the hydroformylation of olefins accompanied by a strong isomerizing activity [40]. The steadily increased and then abruptly plateaued 1‐octene isomerization escorted by the increased aldehyde formation seems to be related to the transformation of [DBUH]^+^[HRu_3_(CO)_11_]^−^ into the new hydride complex. The ^13^C‐NMR spectra at room temperature showed average resonances at *δ* = 202 and 222 ppm, corresponding to carbonyl groups of occurring complexes.

For the ex‐situ NMR investigation of the reaction mixture after 15 in the reactor, the reactor was not purged with argon. Instead, the pressure was slowly released until 1.1 bar, and the obtained yellow solution was transferred to a Young‐NMR tube under argon. Under these conditions a portion of dissolved gases usually remains in solution. The ex situ ^1^H‐NMR spectra showed the formation of the same complex detected by in situ measurements almost quantitatively with a resonance at *δ* = −19.46 ppm in the ^1^H‐NMR spectrum. A very low intensity signal for [DBUH]^+^[HRu_3_(CO)_11_]^−^ at *δ* = −12.65 ppm could also be detected. This could imply an incomplete transformation or the existence of an equilibrium between both species (Figure S5). Comparison of the DBU resonances with pure DBU in acetonitrile‐*d*_3_ revealed the shift in all signals, which corroborates the results of the in situ infrared experiment and agrees with the reports for protonated DBU [66]. The ^13^C{^1^H} VT‐NMR measurements showed a complex pattern of signals for the carbonyl ligands, typical for anionic Ru‐clusters, together with traces of dissolved ^13^CO (Figure S6). The infrared bands associated with the newly formed complex after 16 h, where the band at $\tilde{\nu }$ = 1757 cm^−1^ is typical for *μ*‐bridged carbonyl ligands of an anionic ruthenium carbonyl complex (Figure 3), together with the ^1^H‐NMR hydride resonance at *δ* = −19.46  ppm agree with the spectroscopic data reported for the ruthenium anionic cluster [H_2_Ru_4_(CO)_12_]^2−^ [67].

The NMR spectra measured during the in situ and ex situ NMR spectroscopic investigations of the ruthenium‐catalyzed hydroformylation of 3,3‐Dimethylbut‐1‐ene (neohexene) showed an almost identical behavior related to the formation of the molecular complexes as during the preformation of [Ru_3_(CO)_12_] with DBU under H_2_/CO. Initial formation of [HRu_3_(CO)_11_]^−^ (*δ_H_* = −12.65 ppm) at room temperature was followed by its transformation into [H_2_Ru_4_(CO)_12_]^2−^ (*δ_H_* = −19.46 ppm) at 120°C (Figure S7). These findings reinforced our assumptions about the role of [DBUH]_2_^+^[H_2_Ru_4_(CO)_12_]^2−^ as the active catalytic species or as the resting state of the catalyst during the ruthenium/DBU‐catalyzed hydroformylation of olefins.

Encouraged by our findings during the spectroscopic studies that suggested the formation of [DBUH]_2_^+^[H_2_Ru_4_(CO)_12_]^2−^ as the main molecular complex with certain stability under argon, we proceeded to its isolation for a complete characterization and posterior evaluation as a catalyst for the hydroformylation of olefins. After the reaction of [Ru_3_(CO)_12_] with DBU under H_2_ (35 bar)/CO (5 bar) at 120°C for 48 h, the complex could be isolated as an orange solid in ca. 43% yield. The chemical shifts for the resonances of [DBUH]^+^ in the ^1^H and ^13^C‐NMR spectra shifted clearly with respect to the free base (Figure S8). Comparative tables with the ^1^H and ^13^C chemical shifts for DBU and DBUH^+^ are provided in the supplementary information (Tables S15 and S16). Protonation of DBU causes an increase in shielding for the C4, C6, and C3 nuclei, displaying the major changes in the chemical shifts and a decrease in shielding for the C5a and C10 nuclei in good agreement with the reports of Wiench et al. for the salt [DBUH]^+^[TFA]^−^ [66]. A ^1^H‐^15^N HMBC also confirmed that the N5 nucleus is more shielded, whilst the N1 nucleus is deshielded with the protonation of DBU (Figure S9). The ^15^N resonances for the countercation DBUH^+^ are detected at *δ* = −267.6 (N1) and 258.5 ppm (N5) in comparison with the chemical shifts found for DBU at *δ* = −286.4 (N1) and −183.1 ppm (N5). A coordination of DBU to the ruthenium center can be clearly ruled out, since the coordination of DBU to ruthenium causes a downfield shift of all DBU resonances compared with free DBU, noticeably for the C4 nucleus with a difference of about 10 ppm [68].

**SCHEME 2:**
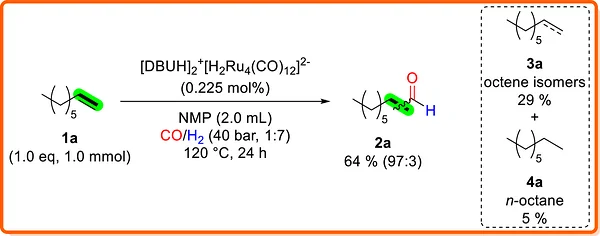
Isolated complex in hydroformylation reactions. (a) Reaction conditions: **1a** (1.0 eq, 1.0 mmol), [DBUH]_2_^+^[H_2_Ru_4_(CO)_12_]^2−^ (0.225 mol%), NMP (2.0 mL), CO/H_2_ (40 bar, 1:7), 120°C, 24 h reaction time. The ratio of linear to all branched products = *n*:*i*. Yields and *n*:*i* are determined by GC analysis using isooctane (57.0 mg, 0.5 mmol) as internal standard.

In the ^13^C‐NMR spectra at room temperature, an average resonance at *δ* = 222 ppm could be observed for the carbonyl groups in good agreement with that reported for the cluster anion [H_2_Ru_4_(CO)_12_]^2−^ [69]. The infrared spectrum of the isolated complex in acetonitrile‐*d*_3_ under argon also shows quite similar band positions and intensity patterns to those reported by Cesari et al. for [NEt_4_]_2_^+^[H_2_Ru_4_(CO)_12_]^2−^ (Figure S10) [67]. Further, we then used the isolated ruthenium complex [DBUH]_2_^+^[H_2_Ru_4_(CO)_12_]^2−^ directly in the hydroformylation of **1a** (Scheme 2), without adding any extra DBU, and to our surprise, we observed that the yield and high linear selectivity of the product aldehyde were nearly the same as compared with the one when adding [Ru_3_(CO)_12_] and DBU separately.

Finally, to confirm the homogeneous nature of the active catalyst system, a mercury poisoning test was also conducted (Scheme S1). The resulting yield of 66% for **2a** in the presence of mercury clearly demonstrates that the catalytic system is purely homogeneous.

**SCHEME 3:**
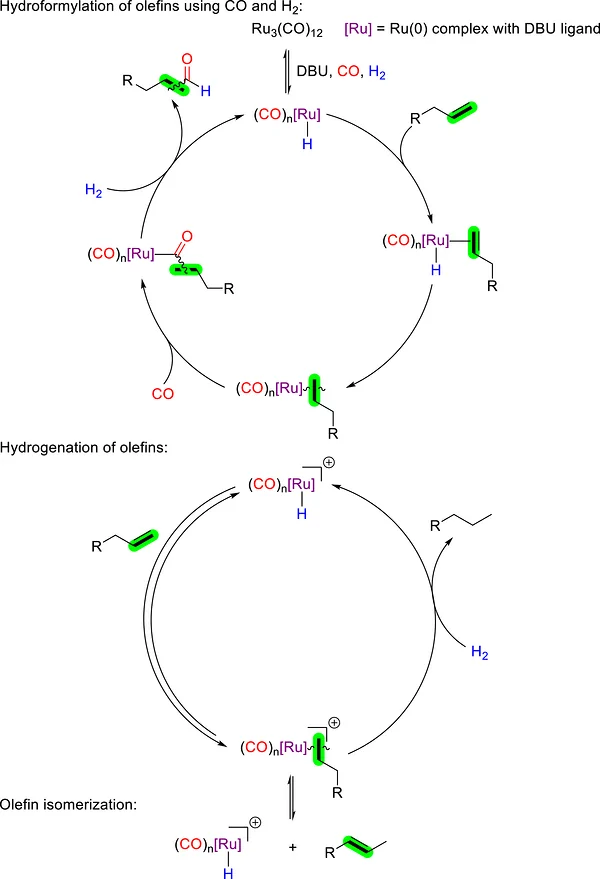
Proposed catalytic cycle of Ruthenium‐Catalyzed Hydroformylation of olefins using CO and H_2_.

The kinetic profiles and the spectroscopic studies suggest that the induction period corresponds to the formation of [DBUH]_2_^+^[H_2_Ru_4_(CO)_12_]^2−^, which could act as the catalytically active intermediate or as the resting state of the catalyst in the hydroformylation cycle. Previous spectroscopic and isotope labeling studies by Süss‐Fink and Herrmann have disclosed that olefin hydroformylation can proceed through intact trinuclear metal cluster intermediates, and importantly, the propionyl intermediate of [HRu_3_(CO)_11_]^−^ catalyzed ethylene hydroformylation was successfully trapped by acidification, providing direct evidence for the cluster‐bound acyl species [70]. Guided by this precedent, we propose a plausible catalytic cycle for [DBUH]_2_^+^[H_2_Ru_4_(CO)_12_]^2−^ (Scheme 3), with analogous intermediate trapping experiments for the tetranuclear system planned as part of a dedicated follow‐up mechanistic study. The olefin first coordinates to the ruthenium center, leading to hydrometallation and the formation of the ruthenium–alkyl intermediate. Then CO insertion between the ruthenium‐alkyl intermediate generates a ruthenium–acyl species, which undergoes reductive elimination to release the aldehyde product and regenerate the Ru─H catalytic species. Apart from the hydroformylation, the Ru─H species can also catalyze olefin hydrogenation, thereby forming saturated alkanes under similar conditions. Furthermore, these Ru–H species are proposed to promote olefin isomerization via a reversible hydrometallation/β‐hydride elimination sequence of the ruthenium–alkyl intermediate, facilitating double‐bond rearrangement pathway consistent with the well‐documented isomerizing activity of ruthenium hydride species reported in the literature [40].

### 2.3 Scope and Applications

**SCHEME 4:**
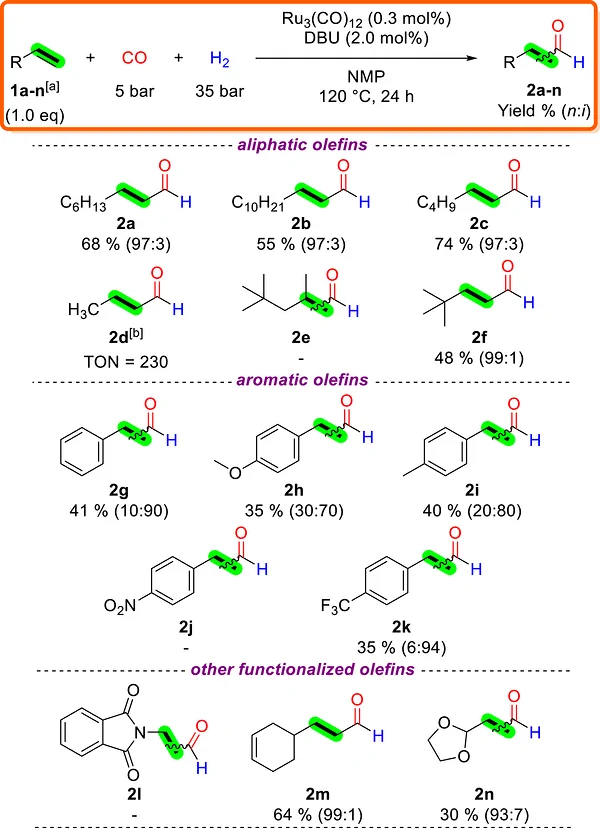
Scope and limitations of the ruthenium catalyzed hydroformylation of different olefins using DBU. (a) Reaction conditions: **1a‐n** (1.0 eq, 1.0 mmol), Ru_3_(CO)_12_ (0.3 mol%), DBU (2.0 mol%), NMP (2.0 mL), CO/H_2_ (40 bar, 1:7), 120°C, 24 h reaction time. (b) Hydroformylation of 1‐propene was carried out in a 100.0 mL autoclave, reaction conditions: **1d** (5.0 bars), Ru_3_(CO)_12_ (0.048 mmol), DBU (0.32 mmol), NMP (20.0 mL), CO/H_2_ (40 bar, 1:7), 120°C, 24 h reaction time. The ratio of linear to all branched products = *n*:*i*. Yields and *n*:*i* are determined by ^1^H NMR and GC analysis using 1,4‐dimethoxybenzene (∼0.5 eq to **1a‐n**) as internal standard.

To demonstrate the broad applicability of the developed Ru‐catalyzed hydroformylation system, a diverse range of olefins, including functionalized substrates as well as industrially relevant feedstocks, were tested under the optimized reaction conditions (Scheme 4). For linear aliphatic alkenes (**1a–d**), the aldehyde yield increases with the decreasing chain length while maintaining the high *n*/*i*‐selectivity across the series. As expected, the non‐isomerizable substrate 3,3‐dimethyl‐1‐butene (**1f**) showed the highest linear selectivity.

Apart from the aliphatic systems, aromatic olefins were also well‐tolerated. In these cases, the regioselectivity shifted toward the branched isomer, likely due to the thermodynamic stability of the corresponding ruthenium–η^3^‐allyl intermediates. For instance, styrene (**1g**) produced the desired aldehyde in 41% yield with a linear‐to‐branched ratio of 10:90. While electron‐donating substituents at the para‐position (**1h** and **1i**) resulted in slightly reduced yields with moderate regioselectivity, electron‐withdrawing groups had a pronounced deactivating effect. No reaction was observed with *para*‐nitrostyrene (**1j**), while 1‐(trifluoromethyl)‐4‐vinylbenzene (**1k**) provided a modest yield (35%) but with excellent selectivity toward the branched isomer (6:94). These results highlight a certain influence of electronic substituents on aromatic rings in both reactivity and regioselectivity.

**SCHEME 5:**
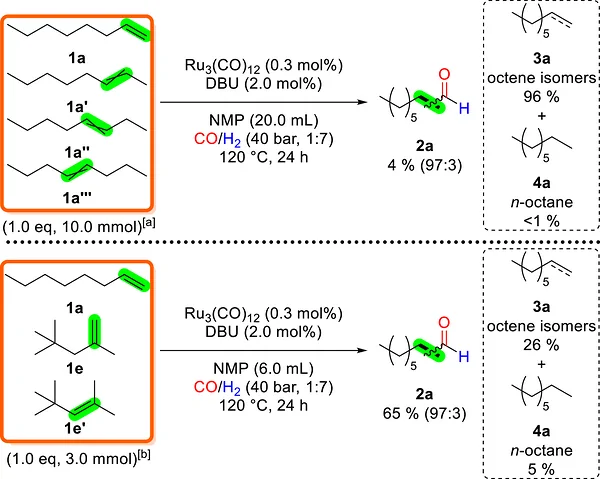
Selective hydroformylation of terminal olefins in mixed olefin mixtures. (a) Reaction conditions: **1a, 1a’, 1a″, 1a‴** (1.0 eq, 10.0 mmol), Ru_3_(CO)_12_ (0.3 mol%), DBU (2.0 mol%), NMP (20.0 mL), CO/H_2_ (40 bar, 1:7), 120°C, 24 h reaction time. The composition of industrial octene mixtures are as follows: **1a** = 6%; **1a′** (E:Z) = 47% (72:28); **1a″** (E:Z) = 34% (81:19); **1a‴** (E:Z) = 13% (100:0). (b) Reaction conditions: **1a, 1e, 1e′** (1.0 eq, 3.0 mmol), Ru_3_(CO)_12_ (0.3 mol%), DBU (2.0 mol%), NMP (6.0 mL), CO/H_2_ (40 bar, 1:7), 120°C, 24 h reaction time. The composition ratio of industrial octene mixtures are as follows: **1a:1e:1e′** = 2:3:1.The ratio of all E‐ to all Z‐olefins = E:Z. The ratio of linear to all branched products = *n*:*i*. Composition, E:Z, yields and *n*:*i* are determined by GC analysis using isooctane (57.0 mg, 0.5 mmol) as internal standard.

Furthermore, we expanded our scope to other functionalized olefins. 2‐Vinylisoindoline‐1,3‐dione (**1l**) was completely unreactive, while 2‐vinyl‐1,3‐dioxolane (**1n**) underwent hydroformylation to afford the aldehyde in modest yield with high linear selectivity. Interestingly, in the case of 1‐vinyl‐3‐cyclohexene (**1m**), which contains both terminal and internal double bonds, the reaction selectively occurred at the terminal olefin with excellent regioselectivity. This particular behavior of activating only the terminal olefins was further confirmed by testing 2,4,4‐trimethyl‐1‐pentene (1**e**), which was completely unreactive under the same conditions. These results suggest that our system can selectively functionalize terminal olefins in the presence of 1,1‐disubstituted or internal double bonds. To further prove this, we performed a reaction with an industrial mixture of terminal and internal octenes, as shown in Scheme 5. Notably, only 1‐octene (the terminal olefin) reacted, while the internal octenes remained intact, showing no reactivity! Even under these conditions, olefin isomerization and hydrogenation did not occur, as confirmed by a control experiment using 2‐octene (ESI, Scheme S2).

From these experimental observations, we conclude that the internal olefins remain completely unreacted under our catalytic conditions. To the best of our knowledge, such exclusive selectivity for terminal olefins, while internal olefins remain untouched, has not been reported for other ruthenium‐based or phosphorus‐free hydroformylation catalysts to date. It is worth noting that even if some degree of reversible isomerization occurs under the reaction conditions, the regioselectivity of the system is not governed by the equilibrium position of double‐bond migration, but rather by the inherent terminal‐olefin selectivity of the active cluster [DBUH]_2_^+^[H_2_Ru_4_(CO)_12_]^2−^, as unambiguously demonstrated by the complete inactivity of 2‐octene under identical catalytic conditions (Scheme S2). Together with our spectroscopic findings, this points out to an intact anionic ruthenium cluster intermediate as active catalyst for the olefin hydroformylation. This unique behavior highlights the uniqueness and potential advantages of our catalytic system.

## 3 Conclusion

We have developed a highly chemo‐ and regioselective hydroformylation catalyst system based on a simple combination of ruthenium carbonyl complexes and catalytic amounts of DBU, an amidine base. The developed phosphorus‐free system operates under mild and air‐stable conditions, providing an alternative to traditional hydroformylation catalysts, which rely on expensive and air‐sensitive phosphine ligands. More importantly, the Ru/DBU catalytic system peculiarly activates the terminal olefins while leaving the internal double bonds untouched, which seems to be a unique behavior for both ruthenium‐based and P‐free catalytic systems. Mechanistic and spectroscopic investigations revealed the formation of the tetranuclear anionic ruthenium cluster [DBUH]_2_^+^[H_2_Ru_4_(CO)_12_]^2−^, which seems to be mainly responsible for the catalytic activity and unusual selectivity. Furthermore, we studied the applicability of the system across a wide range of sub, including functionalized and industrially relevant olefins, which revealed their robustness and tolerance. A detailed kinetic investigation, including identification of the rate‐determining step and full mechanistic elucidation of the catalytic cycle of [DBUH]_2_^+^[H_2_Ru_4_(CO)_12_]^2−^, is currently underway and will be reported in a follow‐up study. In a nutshell, we anticipate that the developed air‐stable, phosphorus‐free catalytic system opens new possibilities for the selective transformation of complex olefin mixtures to oxo products in high selectivity.

## Author Contributions

**Mohamed Niyaz Vellala Syed Ali**: methodology, validation, investigation, writing – original draft. **Ida Ziccarelli**: investigation, writing – review and editing. **Dilver Peña Fuentes**: investigation, methodology. **Christoph Kubis**: methodology, formal analysis, writing – review and editing. **Robert Franke**: funding acquisition, writing – review and editing, supervision. **Ralf Jackstell**: investigation, supervision, writing – review and editing. **Matthias Beller**: conceptualization, supervision, funding acquisition, writing – review and editing.

## Conflicts of Interest

The authors declare no conflicts of interest.

## Supporting information




**Supporting File**: The authors have cited additional references within the Supporting Information [71–73].

## Data Availability

The data that support the findings of this study are available in the Supporting Information of this article.
