# Supplementary material for: Exclusive Hydroformylation of Terminal Olefins: A Phosphorus‐Free Ruthenium System for the Selective Functionalization of Complex Mixtures
Source: Angew Chem Int Ed Engl. 2026 Jul 23;65(39):e3316419. doi: 10.1002/anie.3316419 (PMC13592321; doi:10.1002/anie.3316419)
Supplement: Supplementary file 1 [file ANIE-65-e3316419-s001.pdf]

Exclusive Hydroformylation of Terminal Olefins: A Phosphorus-Free Ruthenium System for the Selective Functionalization of Complex Mixtures

Mohamed Niyaz Vellala Syed Ali†[a], Ida Ziccarelli†[b], Dilver Peña Fuentes[a], Christoph Kubis[a], Robert Franke*[c,d], Ralf Jackstell*[a], Matthias Beller*[a]

[a]Leibniz-Institut für Katalyse e. V., Albert-Einstein-Straße 29a, 18059 Rostock, Germany

[b]Laboratory of Industrial and Synthetic Organic Chemistry (LISOC), Department of Chemistry and Chemical Technologies, University of Calabria, Via P. Bucci 12/C, 87036 Arcavacata di Rende, CS, Italy

[c]Evonik Oxeno GmbH, Paul-Baumann-Straße. 1, 45772 Marl, Germany

[d]Lehrstuhl für Theoretische Chemie, Ruhr-Universität Bochum, 44780 Bochum, Germany

[†] These authors contributed equally to this work.

*Corresponding author. Email: matthias.beller@catalysis.de; ralf.jackstell@catalysis.de; robert.franke@evonik.com

Table of Contents

1. General information: Materials, methods, and procedure S2

2. Supplementary results of reaction condition optimization S4

3. In situ HP-FTIR and HP-NMR spectroscopy studies S25

4. Isolation and characterization of the cluster anionic complex $[DBUH]_2^+[H_2Ru_4(CO)_{12}]^{2-}$ S29

5. NMR Spectra S34

6. Supplementary references S47

1. General information: Materials, methods, and procedure

All commercial reagents were ordered from Alfa Aesar, Abcr, Aldrich, TCI or Strem, CO (4.7) gas was ordered by Linde. Unless otherwise stated, commercial reagents were used without purification. Dry solvents were prepared according to standard procedures.[71] Air- and moisture-sensitive syntheses were performed under argon atmosphere in glassware after heating gun dried under vacuum. Analytical data of literature known compounds were in accordance with reported data. NMR spectra were recorded on Bruker Avance III HD 300 and 400 spectrometers. Multiplets were assigned as s (singlet), d (doublet), t (triplet), dd (doublet of doublet), m (multiplet) and br. s (broad singlet). All measurements were carried out at room temperature unless otherwise stated. NMR measurements at 1 bar were conducted using a normal thin-walled J. Young NMR tube. High pressure variable temperature NMR (VT-HP NMR) experiments were carried out using an NMR-cell setup from Daedalus (Aston, USA) with a 5 mm OD (3.4 mm ID) sapphire tube and a cell holder made of titanium. The assessment of the NMR data was accomplished with TopSpin (Bruker) and Mestrenova (Mestrelab). The chemical shifts are reported relative to the solvent signals (DMSO-d_6: $\delta(^1H)$ = 2.50 ppm, $\delta(^{13}C)$ = 39.5 ppm; $CDCl_3$: $\delta(^1H)$ = 7.26 ppm, $\delta(^{13}C)$ = 77.2 ppm; CD_3CN: δ(1H) = 1.93 ppm, $\delta(^{13}C)$ = 1.3 ppm). Chemical shifts of nitrogen-15 are given relative to a reference frequency calculated from the actual ^1H reference frequency and $\Xi(^{15}N)$ = 10.136 767 MHz, which corresponds to neat nitromethane at 0 ppm.[72]

The in situ infrared spectroscopic measurements were conducted in a customized apparatus consisted of a 25 ml stainless steel cylinder (miniature cylinder, Swagelok) with a magnetic stirrer bar connected to a transmission infrared spectroscopic flow cell made of stainless steel. The reaction solution was circulated continuously between the reactor and the IR cell by a micro annular gear pump (mzr-7255, HNP Mikrosysteme GmbH, Parchim, Germany). The flow velocity of micro annular gear pump was set to 600 rpm (displacement volume = 48 µL). Swagelok stainless steel fittings and tubing

were used for connections. The in situ transmission IR cell was placed in a thermo-controlled heating device, fixed in the optical pathway of a modified Tensor 27 FTIR spectrometer with an MCT-A detector from Bruker. CaF2 (Korth Kristalle GmbH, Kiel, Germany) was used as a window material. The optical path length was 0.4 mm, adjusted with spacer disc elements made of stainless steel placed between the IR windows. FKM O-rings were used for vacuum and gas-tight sealing. FTIR spectra were registered in the spectral range between 3950 and 900 cm^{-1} with a spectral resolution of 2 cm^{-1}. For each individual FTIR spectrum ten scans were collected (double-sided, forward−backward). Preparations of catalyst solutions and the transfer of the liquids were carried out under inert atmosphere (argon) using standard Schlenk techniques. Prior to the in situ FTIR experiment, background spectra of the solvent acetonitrile have been recorded at the same experimental conditions.

Gas chromatography analysis were performed by an HP 6890 chromatograph with a 29.0 m HP5 column. Yields and linear to branched ratios were determined by ^{1}H NMR and GC analysis of the crude reaction mixture. Especially, all the olefins were purified by distillation or silica gel chromatography before testing.

Detailed procedure for the hydroformylation of 1-octene (1.0 mmol scale reaction): The solid starting materials ruthenium precursor, base, and additive were weighed and added to a 4.0 mL screw-cap vial with 6 mm magnetic stirring bar in glovebox. The vial was sealed by a Teflon/Silicone septum and phenolic plastic cap before transferring out of the glovebox. The vial was connected to a Schlenk line with a needle under argon flow. Specific amount of distilled 1-octene (157.0 µL, 1.0 mmol) and solvent (2.0 mL) were injected by microliter and milliliter syringe respectively. The vial was held on an alloy plate and put into a Parr 4560 series autoclave (300.0 mL). At room temperature, the autoclave was flushed with nitrogen gas for thrice. Then carbon monoxide was also flushed twice and charged to specific pressure, then hydrogen was fed to a specific total pressure. The reaction was heated to specific temperature and stirred (450 rpm) for a specific reaction time. Afterwards, the autoclave was cooled to room temperature using an ice-water bath, then the gas was carefully released. Isooctane (83.0 µL, 57.0 mg, 0.5 mmol) was added to the solution as internal standard, and the solution was filtered by celite and analyzed by gas chromatography.

2. Supplementary results of reaction condition optimization

Table S1 | Study of the variation of metal precursor and base

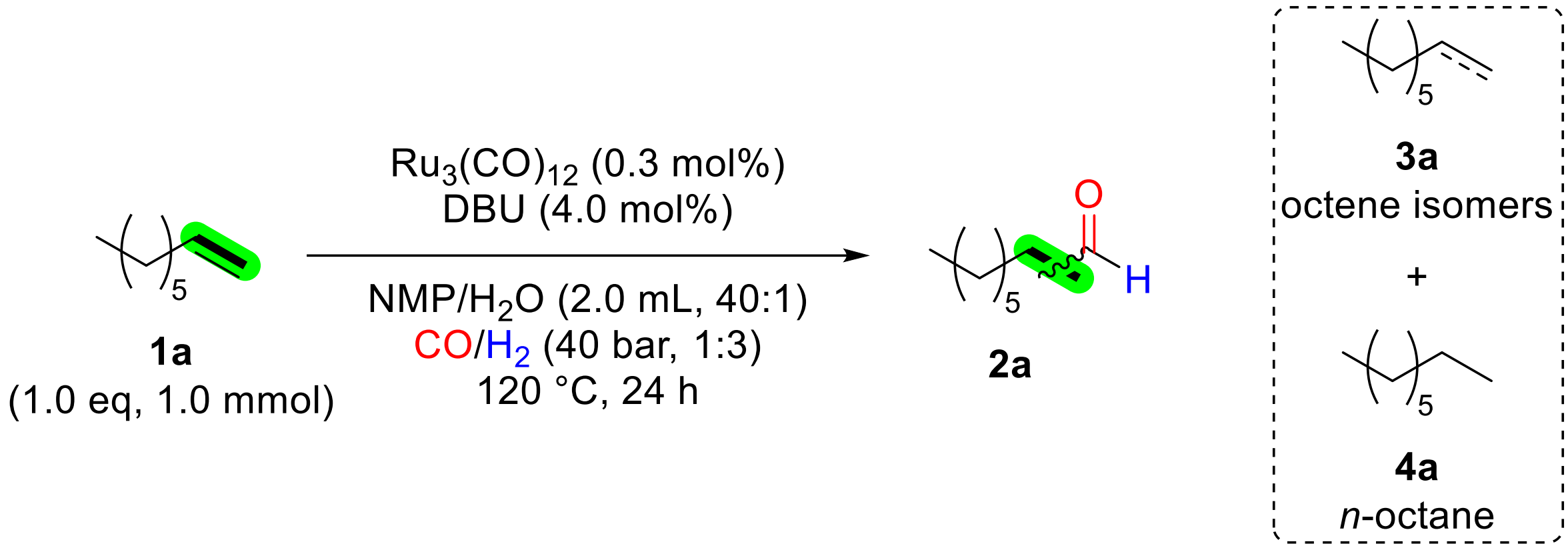

Entry	Variations	Conversion of 1a [%]	Yield [%]		
			**2a (*n*:*i*)**	**3a**	**4a**
1	-	95	53 (97:3)	38	4
2	Rh(acac)$(CO)_2$ instead of $Ru_3(CO)_{12}$ without DBU	99	85 (53:47)	4	10
3	$Co_2(CO)_8$ instead of $Ru_3(CO)_{12}$ without DBU	16	-	16	-

4	Rh(acac)(CO)2 instead of Ru3(CO)12 with DBU	0	-	-	-
5	Co2(CO)8 instead of Ru3(CO)12 with DBU	0	-	-	-
6	K_2CO_3 instead of DBU	25	14 (80:20)	5	6
7	KO^{t}Bu instead of DBU	27	11 (75:25)	8	8

Reaction conditions: **1a** (1.0 eq, 1.0 mmol), Metal precursor ([M] = 0.9 mol%), Base (4.0 mol%), NMP/H_2O (2.0 mL, 40:1), CO/H_2 (40 bar, 1:3), 120 °C, 24 hours reaction time. The ratio of linear to all branched products = *n*:*i*. Yields and *n*:*i* are determined by GC analysis using isooctane (57.0 mg, 0.5 mmol) as internal standard.

Table S2 | Study of the variation of additive

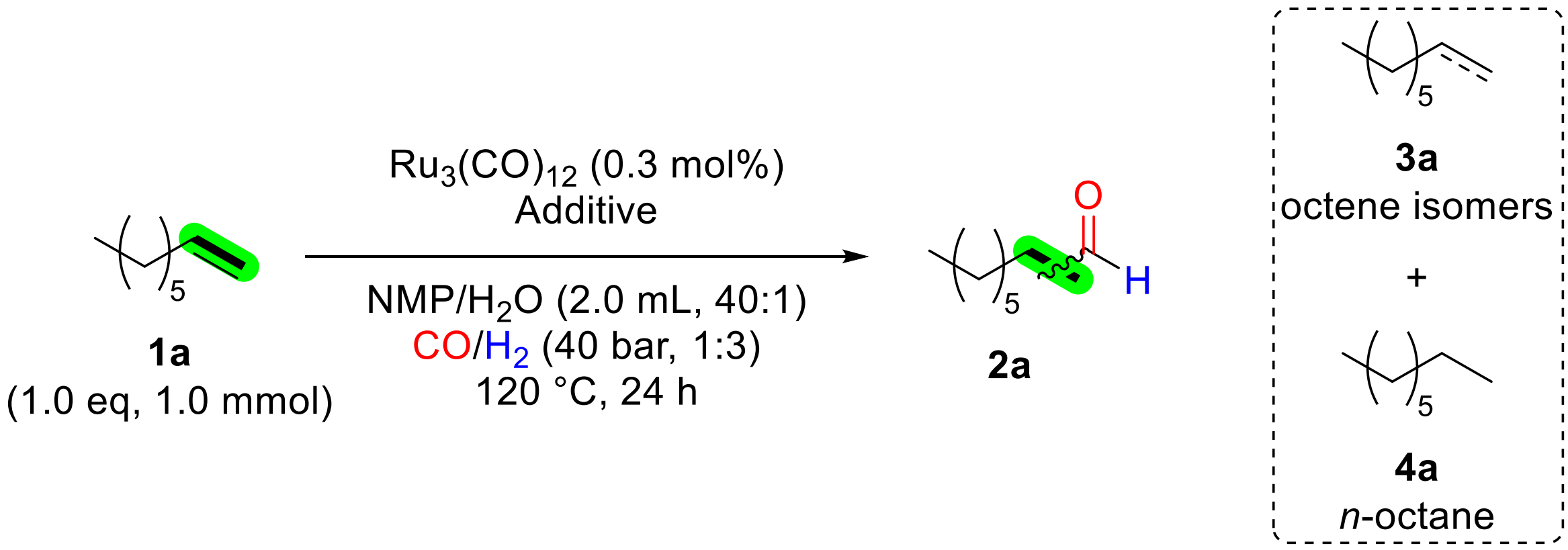

Entry	Additive	Conversion of 1a [%]	Yield [%]

			2a (*n*:*i*)	3a	4a
1	LiCl	98	59 (85:15)	30	9
2	LiBr	96	52 (82:18)	38	6
3	LiI	95	51 (86:14)	37	7
4	$LiBF_4$	89	37 (98:2)	49	3

Reaction conditions: **1a** (1.0 eq, 1.0 mmol), $Ru_3(CO)_{12}$ (0.3 mol%), Additive (10.0 mol%), NMP/H_2O (2.0 mL, 40:1), CO/H_2 (40 bar, 1:3), 120 °C, 24 hours reaction time. The ratio of linear to all branched products = *n*:*i*. Yields and *n*:*i* are determined by GC analysis using isooctane (57.0 mg, 0.5 mmol) as internal standard.

Table S3 | Study of the variation of mol% of LiCl

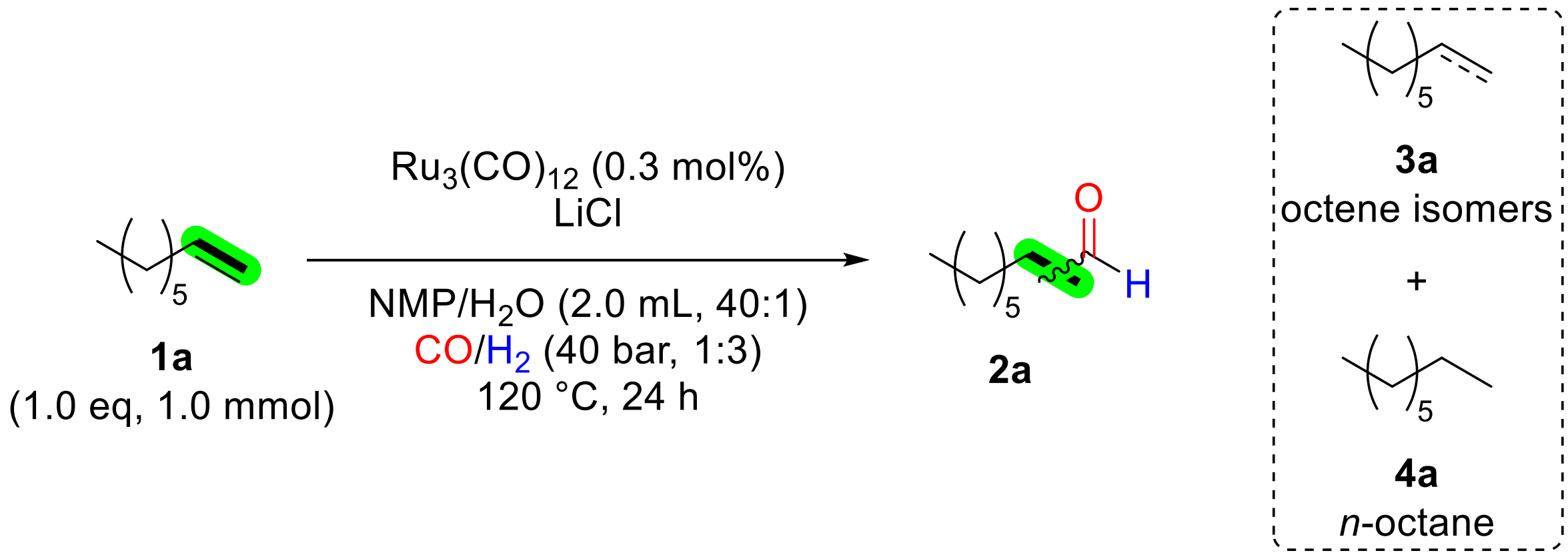

Entry	LiCl [mol%]	Conversion of 1a [%]	Yield [%]

			2a (*n*:*i*)	3a	4a
1	1	96	44 (80:20)	44	8
2	5	95	53 (85:15)	33	9
3	10	98	59 (85:15)	30	9
4	20	98	55 (86:14)	34	9
5	25	99	54 (87:13)	38	7
6	50	99	48 (87:13)	41	10

Reaction conditions: **1a** (1.0 eq, 1.0 mmol), $Ru_3(CO)_{12}$ (0.3 mol%), LiCl (mol%), NMP/H_2O (2.0 mL, 40:1), CO/H_2 (40 bar, 1:3), 120 °C, 24 hours reaction time. The ratio of linear to all branched products = *n*:*i*. Yields and *n*:*i* are determined by GC analysis using isooctane (57.0 mg, 0.5 mmol) as internal standard.

Table S4 | Study of the variation of mol% of DBU

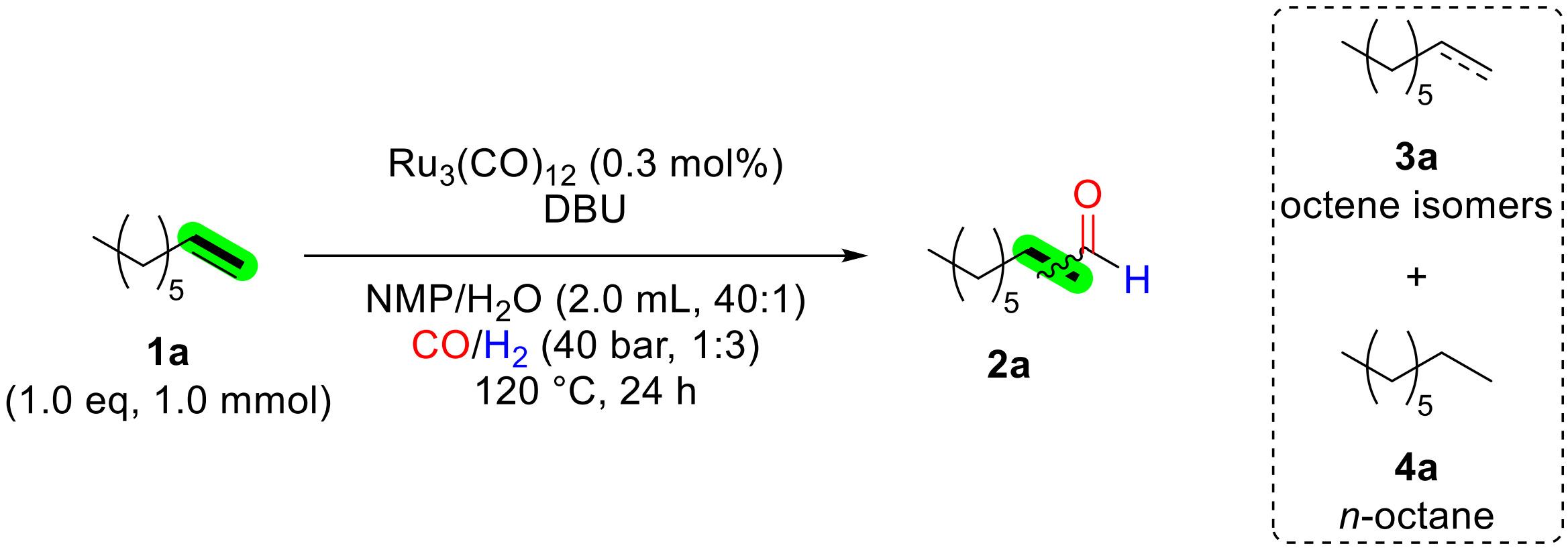

Entry	DBU [mol%]	Conversion of 1a [%]	Yield [%]		
			**2a** (*n*:*i*)	**3a**	**4a**
1	0.05	95	22 (97:3)	67	6
2	0.5	94	30 (97:3)	59	5
3	1	95	46 (97:3)	43	6
4	2	94	57 (97:3)	33	4
5	4	95	53 (97:3)	38	4
6	6	95	45 (97:3)	45	5
7	8	95	38 (97:3)	52	5

8	12	95	28 (97:3)	62	5
9	16	95	25 (97:3)	65	5

Reaction conditions: **1a** (1.0 eq, 1.0 mmol), $Ru_3(CO)_{12}$ (0.3 mol%), DBU (mol%), NMP/H_2O (2.0 mL, 40:1), CO/H_2 (40 bar, 1:3), 120 °C, 24 hours reaction time. The ratio of linear to all branched products = *n*:*i*. Yields and *n*:*i* are determined by GC analysis using isooctane (57.0 mg, 0.5 mmol) as internal standard.

Table S5 | Study of the variation of solvent

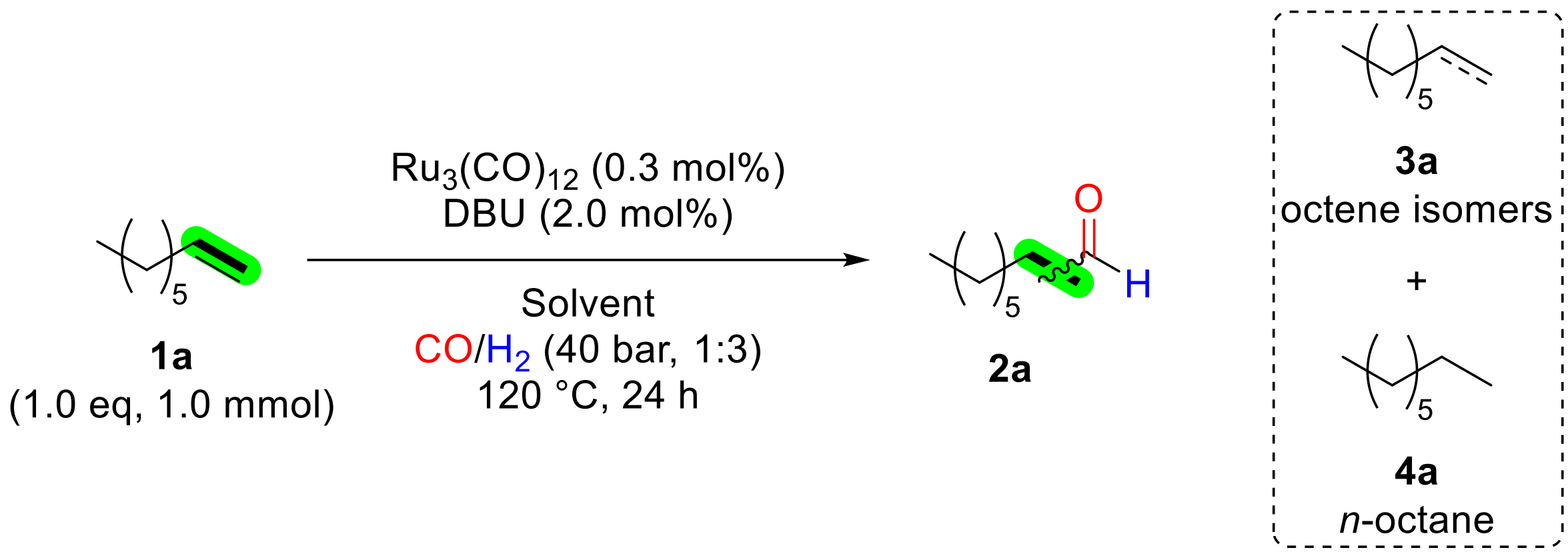

Entry	Solvent	Conversion of 1a [%]	Yield [%]		
			**2a (*n*:*i*)**	**3a**	**4a**
1	MeCN /H_2O (40:1)	82	54 (98:2)	24	4
2	MeCN	81	62 (98:2)	13	6

3	DMA /H_2O (40:1)	89	51 (97:3)	33	5
4	DMA	91	60 (97:3)	26	5
5	NMP/H_2O (40:1)	94	57 (97:3)	33	4
6	NMP	94	68 (97:3)	21	5
7	PC/H_2O (40:1)	80	55 (98:2)	20	5
8	PC	80	63 (98:2)	13	4
9	THF/H_2O (40:1)	91	32 (97:3)	54	5
10	THF	90	40 (97:3)	44	6
11	MTBE/H_2O (40:1)	88	7 (90:10)	75	6
12	MTBE	96	9 (90:10)	83	4
13	Toluene /H_2O (40:1)	89	4 (95:5)	80	5
14	Toluene	88	6 (95:5)	78	4

Reaction conditions: **1a** (1.0 eq, 1.0 mmol), $Ru_3(CO)_{12}$ (0.3 mol%), DBU (2.0 mol%), Solvent (2.0 mL), CO/H_2 (40 bar, 1:3), 120 °C, 24 hours reaction time. The ratio of linear to all branched products = *n:i*. Yields and *n:i* are determined by GC analysis using isooctane (57.0 mg, 0.5 mmol) as internal standard.

Table S6 | Study of the variation of CO/H_2 pressure

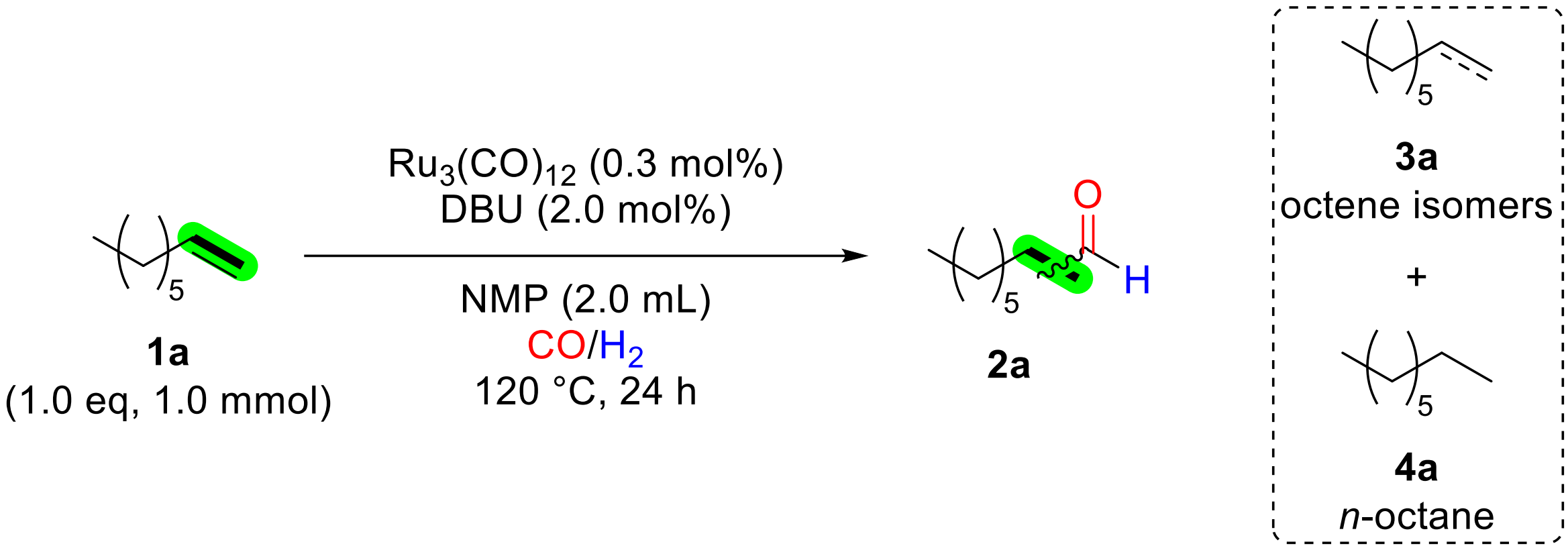

Entry	CO/H_2 [40 bar, x:y]	Conversion of 1a [%]	Yield [%]		
			2a (*n*:*i*)	3a	4a
1	3:1	92	29 (97:3)	55	8
2	1:1	94	42 (97:3)	47	5
3	1:3	94	68 (97:3)	21	5
4	1:7	96	68 (97:3)	22	6
5	1:39	98	34 (97:3)	24	40

Reaction conditions: **1a** (1.0 eq, 1.0 mmol), $Ru_3(CO)_{12}$ (0.3 mol%), DBU (2.0 mol%), NMP (2.0 mL), CO/H_2 (40 bar, x:y), 120 °C, 24 hours reaction time. The ratio of linear to all branched products = *n*:*i*. Yields and *n*:*I* are determined by GC analysis using isooctane (57.0 mg, 0.5 mmol) as internal standard.

Table S7 | Study of the variation of temperature

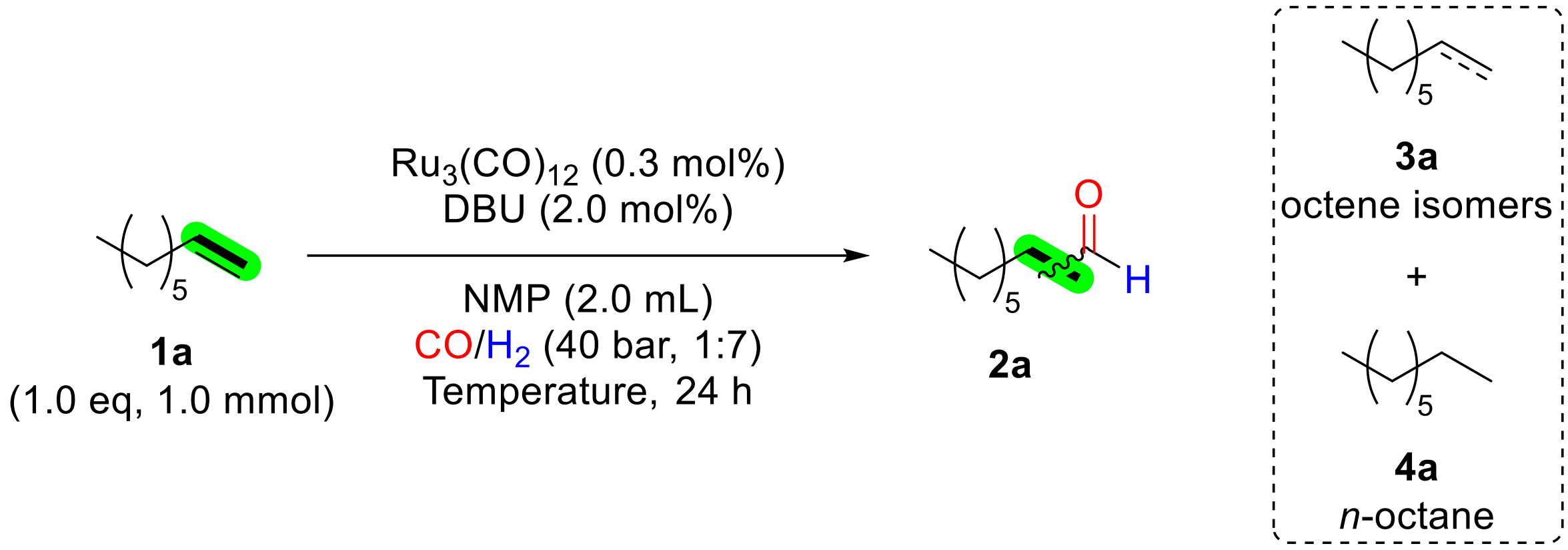

Entry	Temperature [°C]	Conversion of 1a [%]	Yield [%]		
			**2a (*n*:*i*)**	**3a**	**4a**
1	100	60	33 (97:3)	20	7
2	120	96	68 (97:3)	22	6
3	140	99	75 (97:3)	19	5
4	160	99	74 (97:3)	20	5

Reaction conditions: **1a** (1.0 eq, 1.0 mmol), $Ru_3(CO)_{12}$ (0.3 mol%), DBU (2.0 mol%), NMP (2.0 mL), CO/H_2 (40 bar, 1:7), Temperature (°C), 24 hours reaction time. The ratio of linear to all branched products = *n*:*i*. Yields and *n*:*I* are determined by GC analysis using isooctane (57.0 mg, 0.5 mmol) as internal standard.

Table S8 | Study of the kinetic profile by sample taking experiment

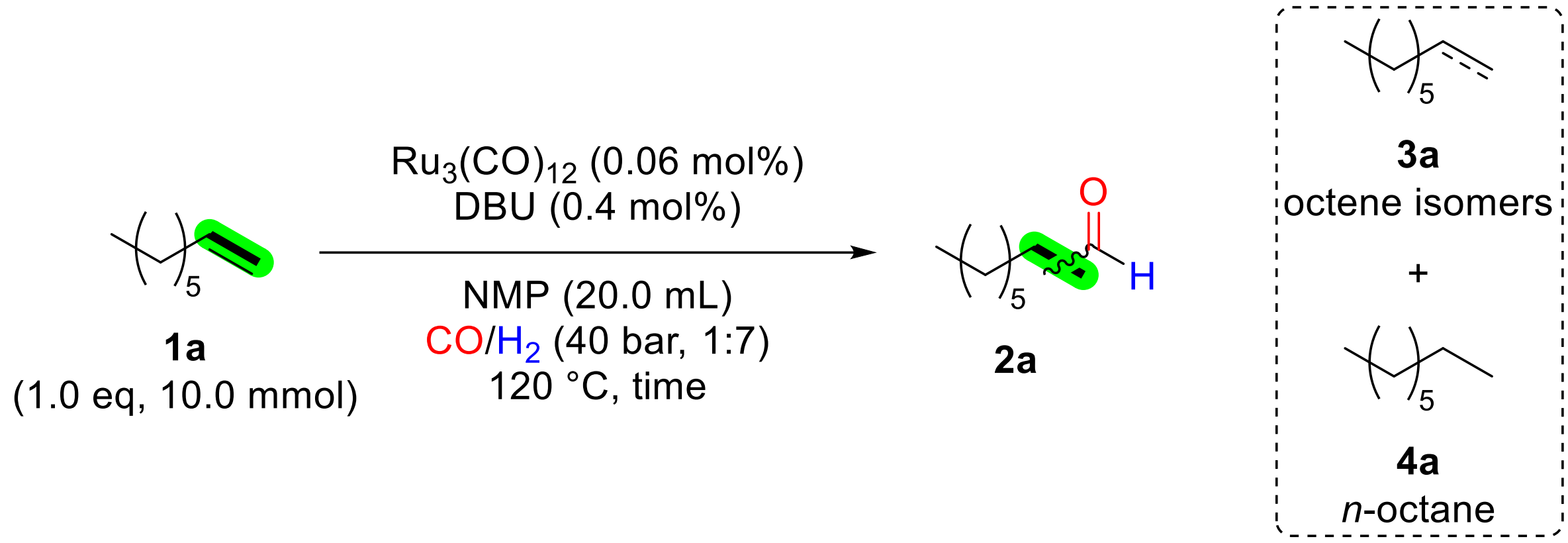

Entry	time [h]	Conversion of 1a [%]	Yield [%]	
			**2a**	**3a & 4a**
1	0	0	0	0
2	2	18	9	9 (major **3a**)
3	4	33	14	19 (major **3a**)
4	6	42	19	23 (major **3a**)
5	8	52	23	29 (major **3a**)
6	10	58	29	29 (major **3a**)

7	12	64	35	29 (major **3a**)
8	20	86	57	29 (major **3a**)
9	22	91	62	29 (major **3a**)
10	24	95	66	29 (major **3a**)
11	26	98	69	29 (major **3a**)
12	28	98	69	29 (major **3a**)
13	30	98	69	29 (major **3a**)

Reaction conditions: **1a** (1.0 eq, 10.0 mmol), $Ru_3(CO)_{12}$ (0.06 mol%), DBU (0.4 mol%), NMP (20.0 mL), CO/H_2 (40 bar, 1:7), 120 °C, time (h). The ratio of linear to all branched products = *n*:*i*. Yields and *n*:*i* are determined by GC analysis using isooctane (57.0 mg, 0.5 mmol) as internal standard.

Table S9 | Turnover number (TON) & Turnover frequency (TOF)

TON	$= \frac{\text{Amount of the product formed (mmol)}}{\text{Amount of the catalyst used (mmol)}}$ $= \frac{6.9\text{ (mmol)}}{0.006\text{ (mmol)}} = 1150$
TOF	$= \frac{\text{TON value for initial 2 hours}}{\text{time (h)}} = \frac{150}{2\text{ (h)}} = 75\ h^{-1}$

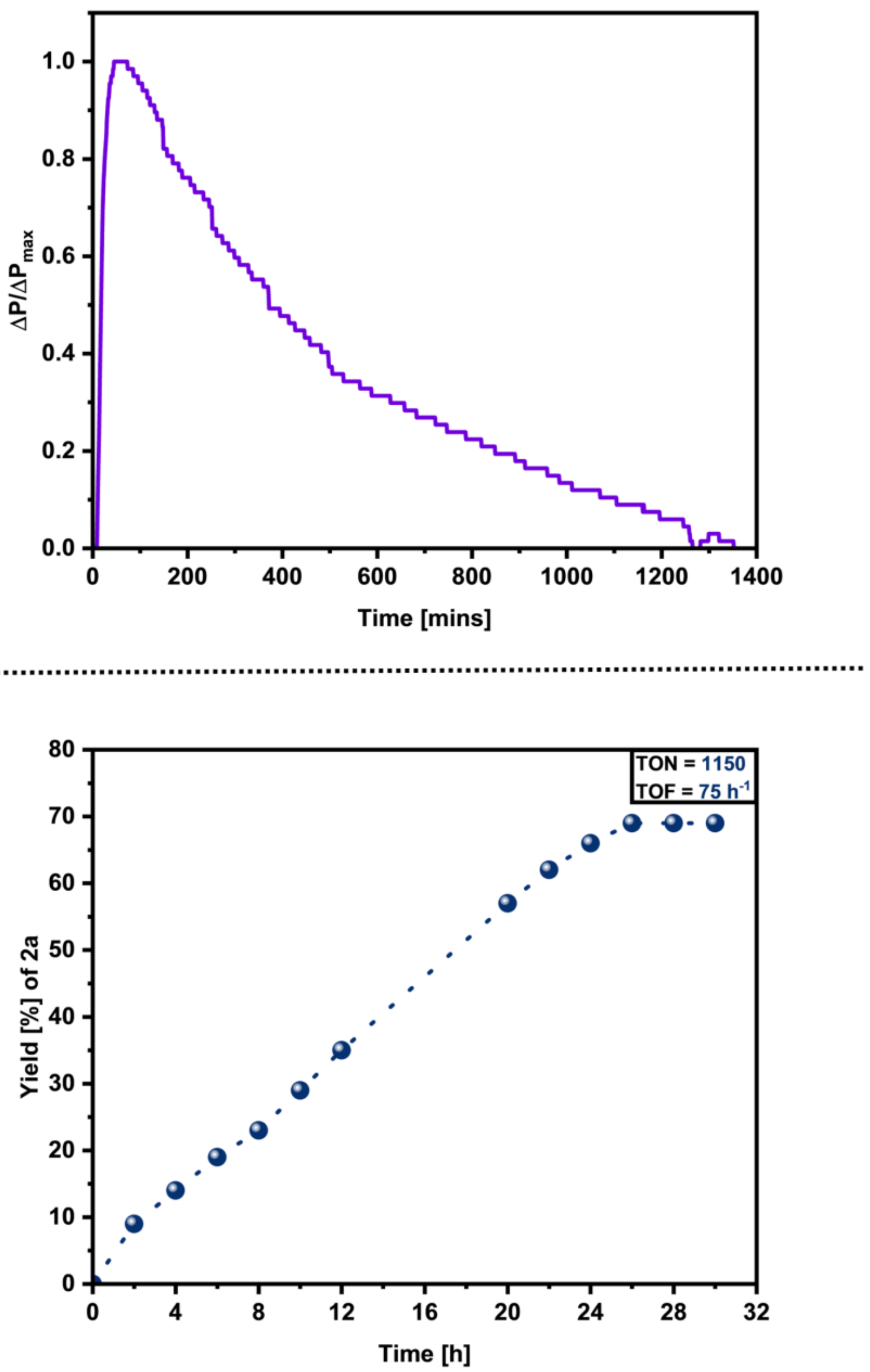

Figure S1 | Gas consumption analysis and kinetic profile

Table S10 | Behavior of the system in CO/H_2 without adding DBU

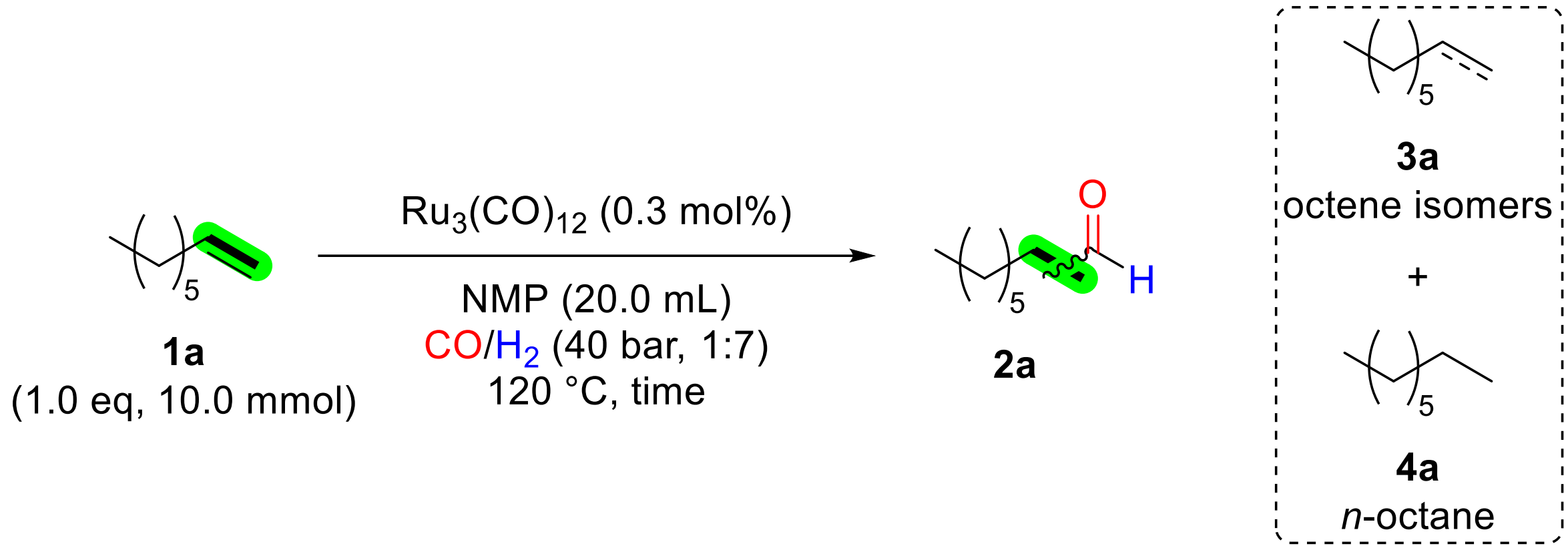

Entry	time [min]	Yield [%]		
		**1a**	**2a**	**3a & 4a**
1	0	100	0	0
2	10	81	3	16 (major **3a**)
3	20	77	4	19 (major **3a**)
4	40	68	5	27 (major **3a**)
5	50	52	6	42 (major **3a**)
6	60	46	7	47 (major **3a**)

7	120	28	9	63 (major **3a**)
8	180	21	11	68 (major **3a**)
9	300	15	14	71 (major **3a**)
10	360	10	18	72 (major **3a**)

Reaction conditions: **1a** (1.0 eq, 10.0 mmol), $Ru_3(CO)_{12}$ (0.3 mol%), NMP (20.0 mL), CO/H_2 (40 bar, 1:7), 120 °C, time (min). The ratio of linear to all branched products = *n*:*i*. Yields and *n*:*i* are determined by GC analysis using isooctane (57.0 mg, 0.5 mmol) as internal standard.

Table S11 | Behavior of the system in CO/H_2 with adding DBU

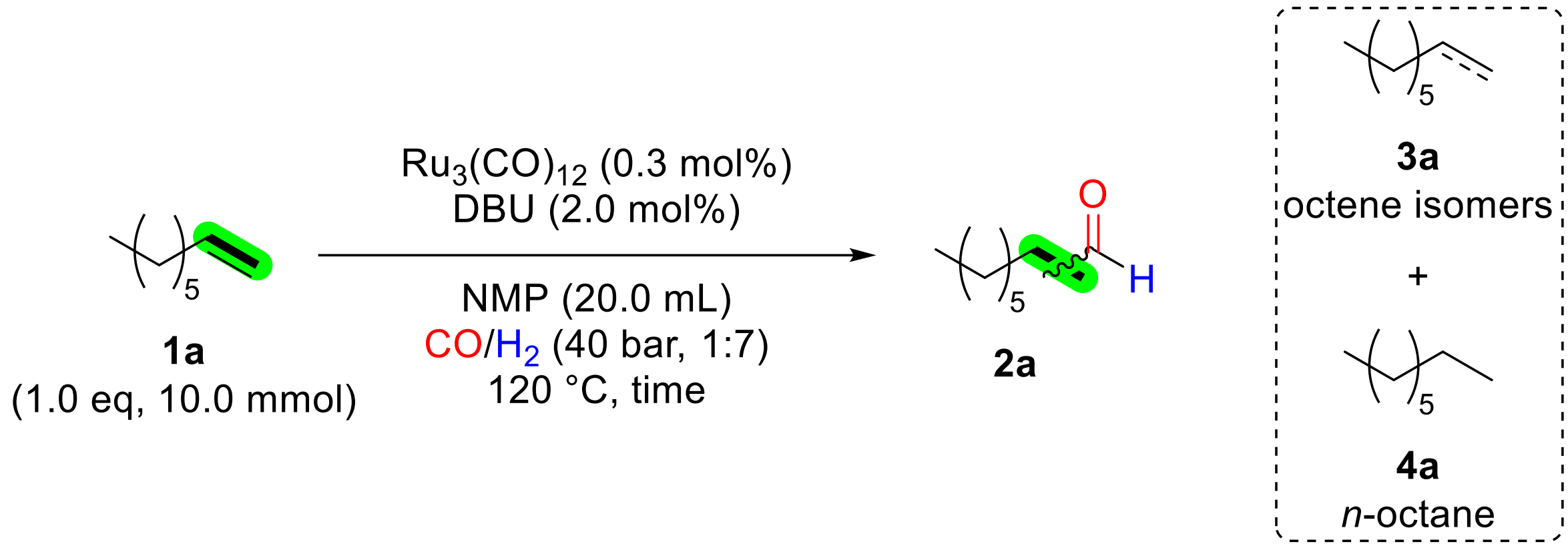

Entry	time [min]	Yield [%]		
		**1a**	**2a**	**3a & 4a**

1	0	100	0	0
2	10	94	0	6 (major **3a**)
3	20	89	0	11 (major **3a**)
4	40	78	7	15 (major **3a**)
5	50	73	8	19 (major **3a**)
6	60	63	11	26 (major **3a**)
7	120	57	19	24 (major **3a**)
8	180	48	26	26 (major **3a**)
9	300	34	40	26 (major **3a**)
10	360	29	45	26 (major **3a**)

Reaction conditions: **1a** (1.0 eq, 10.0 mmol), $Ru_3(CO)_{12}$ (0.3 mol%), DBU (2.0 mol%), NMP (20.0 mL), CO/H_2 (40 bar, 1:7), 120 °C, time (min). The ratio of linear to all branched products = *n*:*i*. Yields and *n*:*i* are determined by GC analysis using isooctane (57.0 mg, 0.5 mmol) as internal standard.

Table S12 | Behavior of the system in H_2 without adding DBU

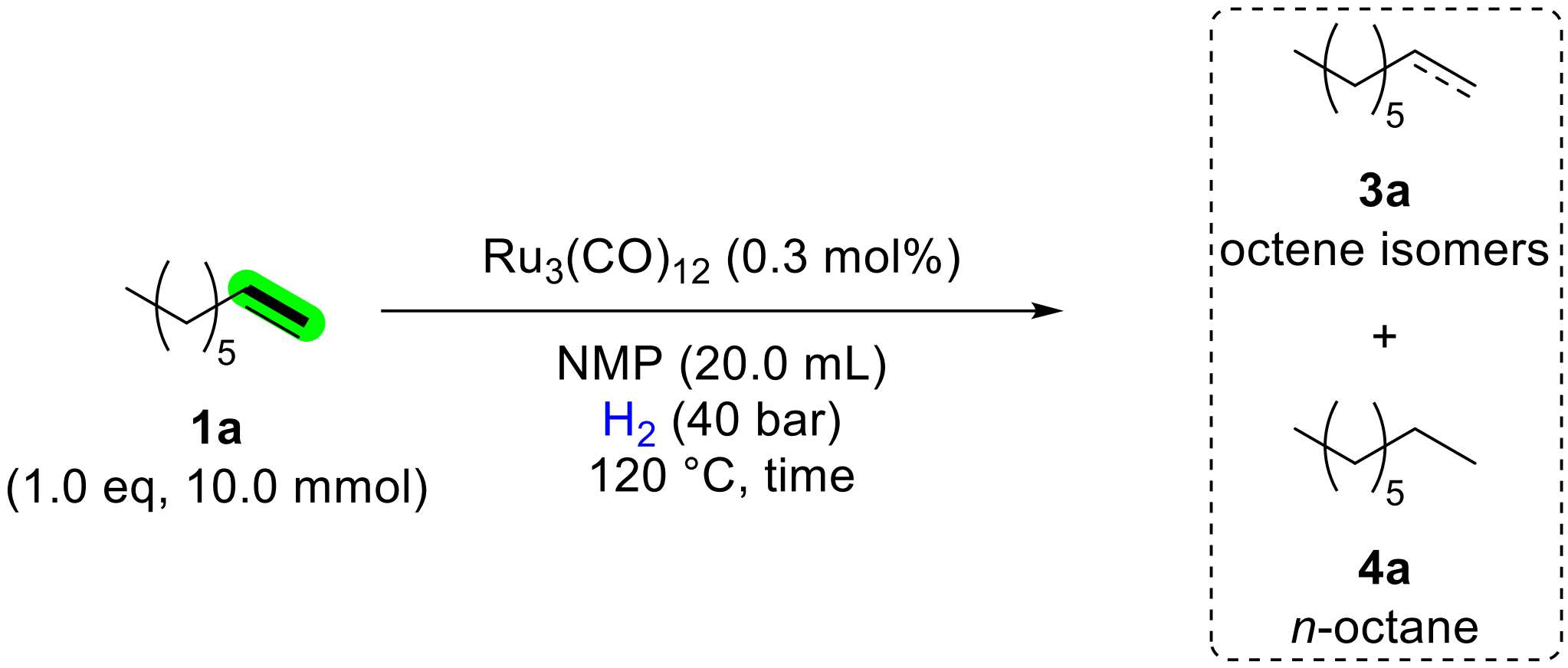

Entry	time [min]	Yield [%]		
		1a	3a	4a
1	0	100	0	0
2	15	2	57	41
3	30	2	50	48
4	45	2	46	52
5	60	2	43	55
6	120	2	39	59
7	180	2	31	67

Reaction conditions: **1a** (1.0 eq, 10.0 mmol), $Ru_3(CO)_{12}$ (0.3 mol%), NMP (20.0 mL), H_2 (40 bar), 120 °C, time (min). The ratio of linear to all branched products = *n*:*i*. Yields and *n*:*i* are determined by GC analysis using isooctane (57.0 mg, 0.5 mmol) as internal standard.

Table S13 | Behavior of the system in H_2 with adding DBU

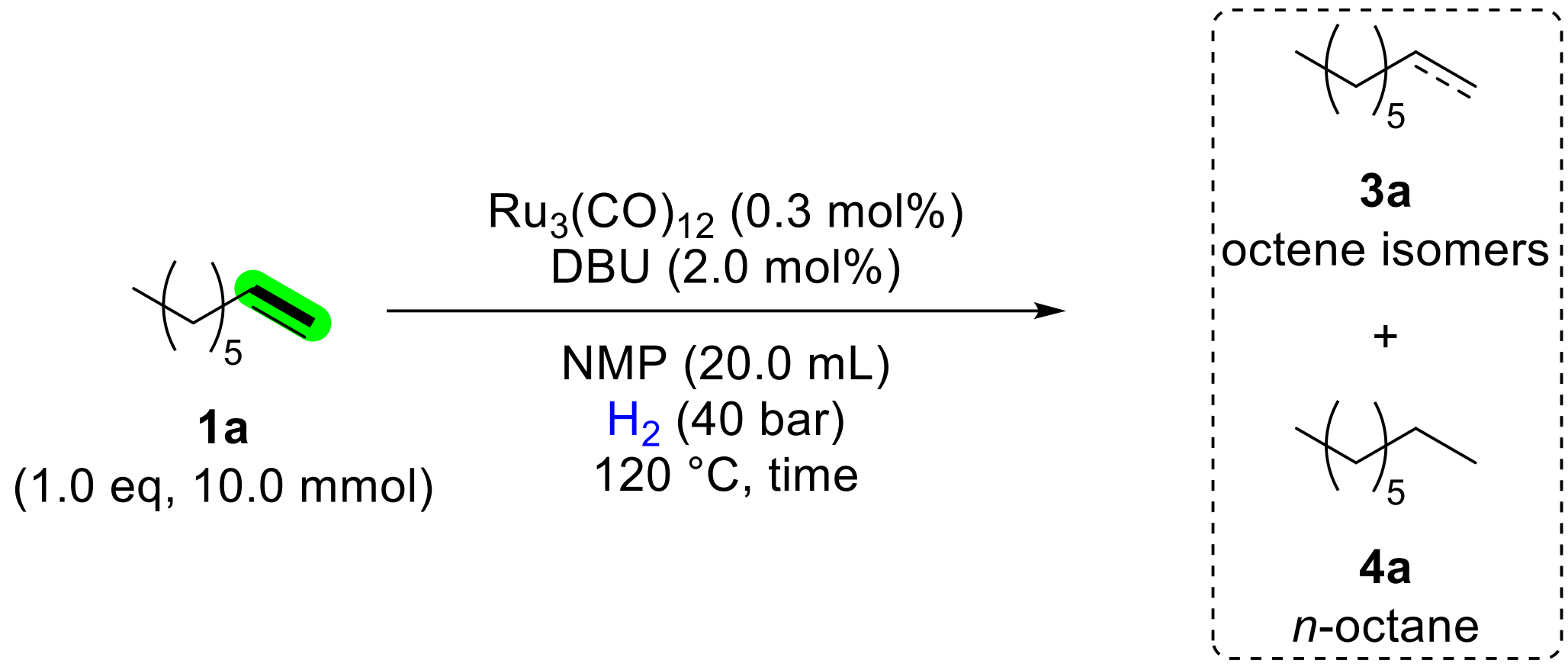

Entry	time [min]	Yield [%]		
		**1a**	**3a**	**4a**
1	0	100	0	0
2	15	48	15	37
3	30	33	21	46
4	45	14	27	59

5	60	8	27	65
6	120	2	27	71
7	180	2	27	71

Reaction conditions: **1a** (1.0 eq, 10.0 mmol), $Ru_3(CO)_{12}$ (0.3 mol%), DBU (2.0 mol%), NMP (20.0 mL), H_2 (40 bar), 120 °C, time (min). The ratio of linear to all branched products = *n*:*i*. Yields and *n*:*i* are determined by GC analysis using isooctane (57.0 mg, 0.5 mmol) as internal standard.

Table S14 | Behavior of the system after pre-activating in CO/H_2 with adding DBU

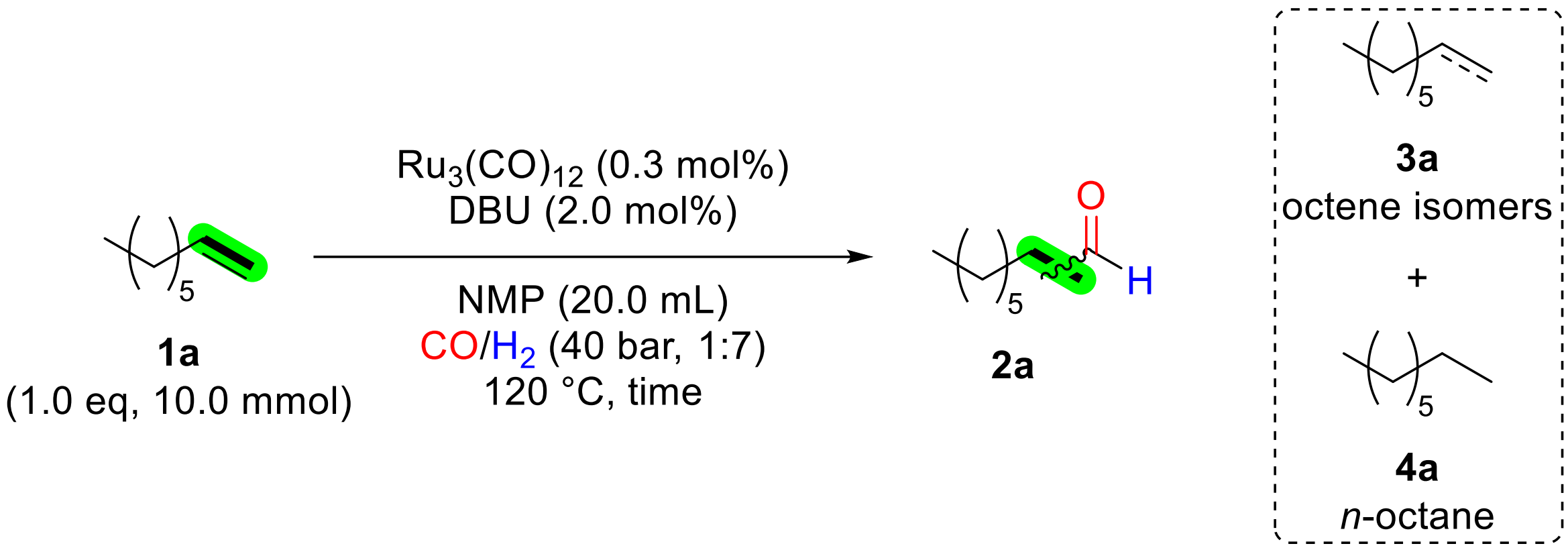

Entry	time [min]	Yield [%]		
		**1a**	**2a**	**3a & 4a**
1	0	100	0	0

2	10	78	10	12 (major **3a**)
3	20	63	18	19 (major **3a**)
4	40	51	25	24 (major **3a**)
5	50	39	36	25 (major **3a**)
6	60	30	45	25 (major **3a**)
7	120	16	59	25 (major **3a**)
8	180	8	67	25 (major **3a**)
9	300	8	67	25 (major **3a**)
10	360	8	67	25 (major **3a**)

Reaction conditions: **1a** (1.0 eq, 10.0 mmol), $Ru_3(CO)_{12}$ (0.3 mol%), DBU (2.0 mol%), NMP (20.0 mL), CO/H_2 (40 bar, 1:7), 120 °C, time (min). Performed a pre-activation using $Ru_3(CO)_{12}$ (0.3 mol%), DBU (2.0 mol%), NMP (20.0 mL), CO/H_2 (40 bar, 1:7), 120 °C, 30 minutes. The ratio of linear to all branched products = *n*:*i*. Yields and *n*:*i* are determined by GC analysis using isooctane (57.0 mg, 0.5 mmol) as internal standard.

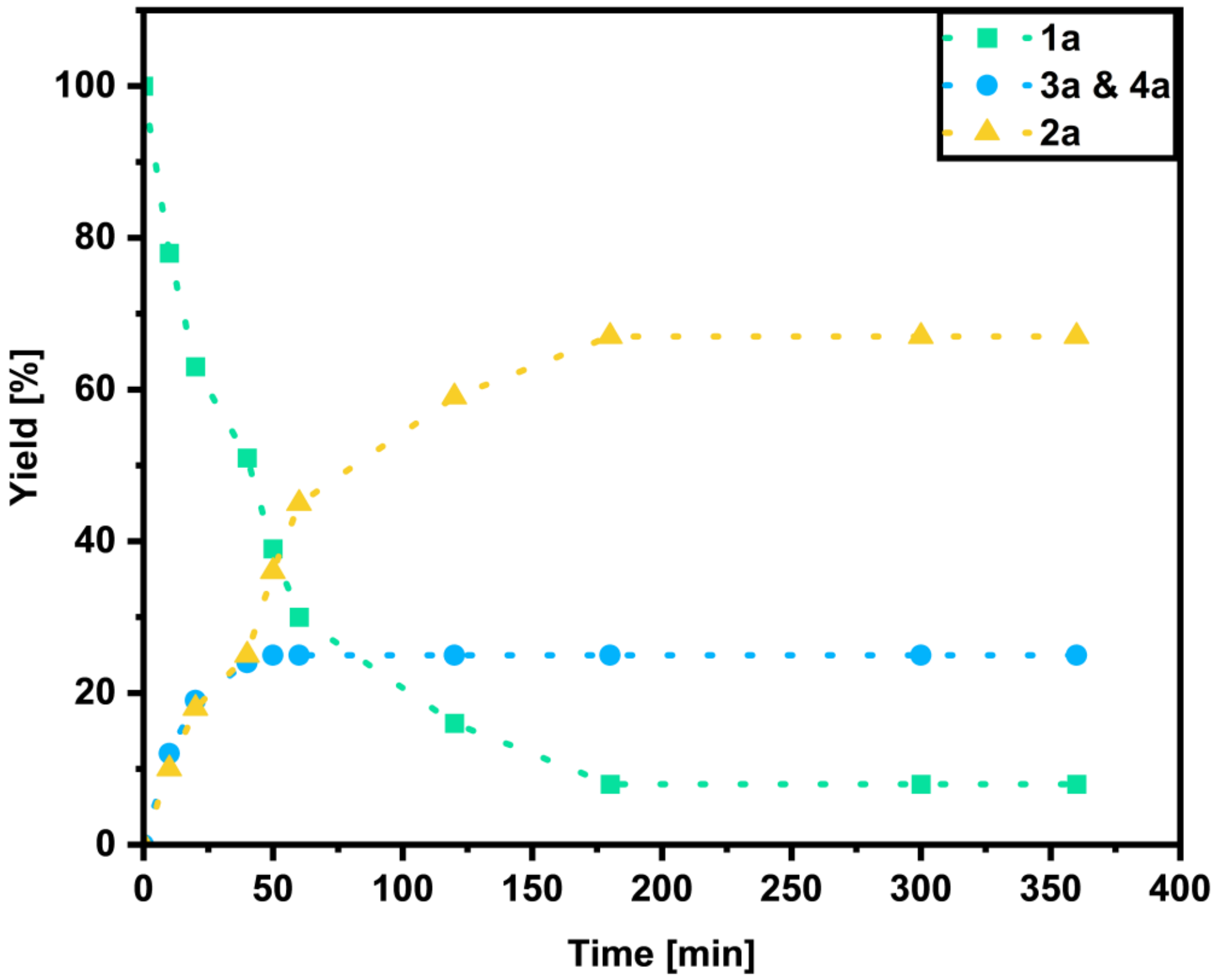

Figure S2 | Kinetic effect of catalyst pre-activation

Scheme S1 | Mercury-Rapid Test demonstrating homogeneous catalytic activity

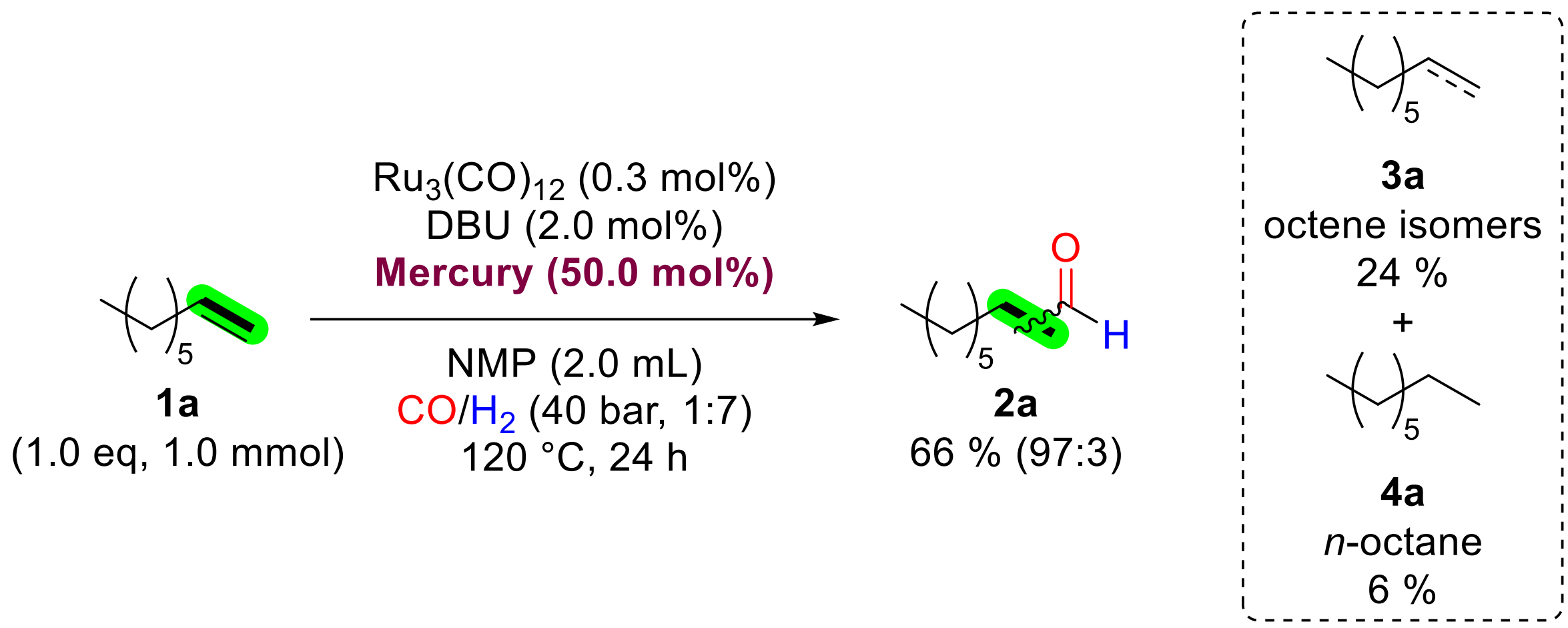

Reaction conditions: **1a** (1.0 eq, 1.0 mmol), $Ru_3(CO)_{12}$ (0.3 mol%), DBU (2.0 mol%), Mercury (50.0 mol%), NMP (2.0 mL), CO/H_2 (40 bar, 1:7), 120 °C, 24 hours reaction time. The ratio of linear to all branched products = *n*:*i*. Yields and *n*:*i* are determined by GC analysis using isooctane (57.0 mg, 0.5 mmol) as internal standard.

Scheme S2 | Control experiment showing absence of isomerization and hydrogenation of internal olefins

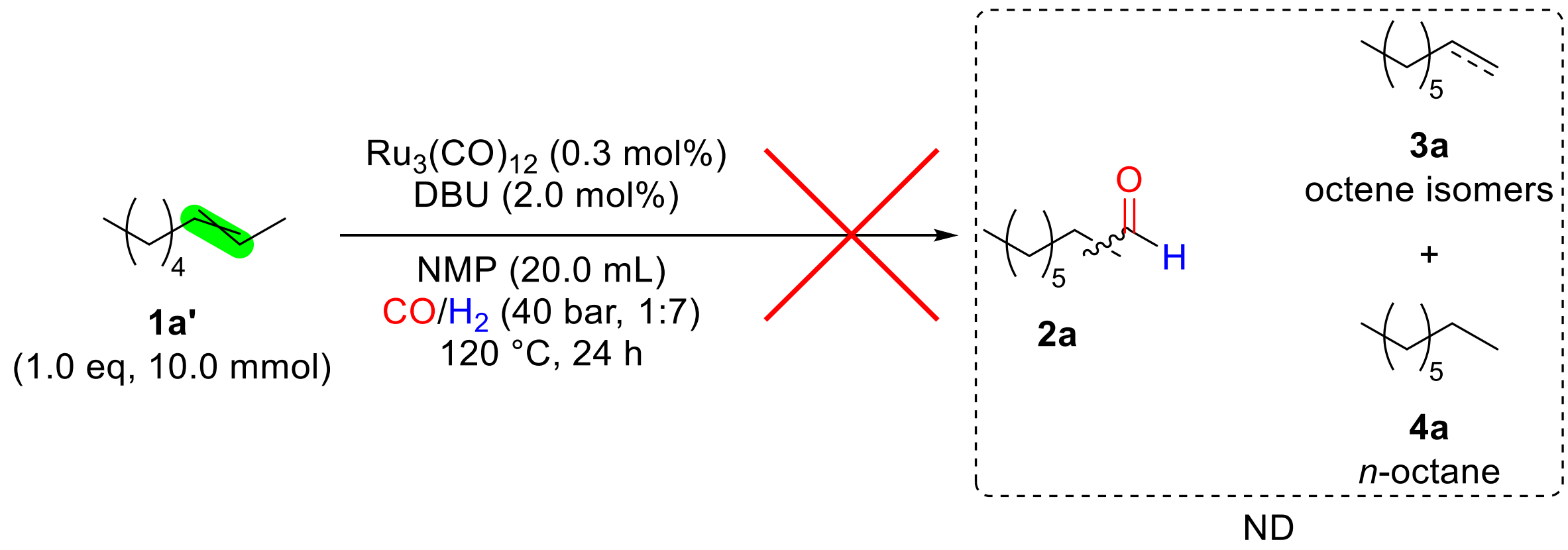

Reaction conditions: **1a'** (1.0 eq, 10.0 mmol), $Ru_3(CO)_{12}$ (0.3 mol%), DBU (2.0 mol%), NMP (20.0 mL), CO/H_2 (40 bar, 1:7), 120 °C, 24 hours reaction time. The ratio of linear to all branched products = *n*:*i*. Yields and *n*:*i* are determined by GC analysis using isooctane (57.0 mg, 0.5 mmol) as internal standard. ND: Not detected.

3. In situ HP-FTIR and HP-NMR spectroscopy studies

3.1. In situ HP-FTIR

[$Ru_3(CO)_{12}$] (1.9 mg, 0.003 mmol) and DBU (2.9 µL, 0.02 mmol) were dissolved in dry CH_3CN (2.0 mL) and stirred under argon for 4 h. The resulting red solution was transferred into the FTIR apparatus and heated at 120 °C under carbon monoxide, p(CO) = 5 bar and hydrogen (p(H_2) = 35 bar, while monitoring.

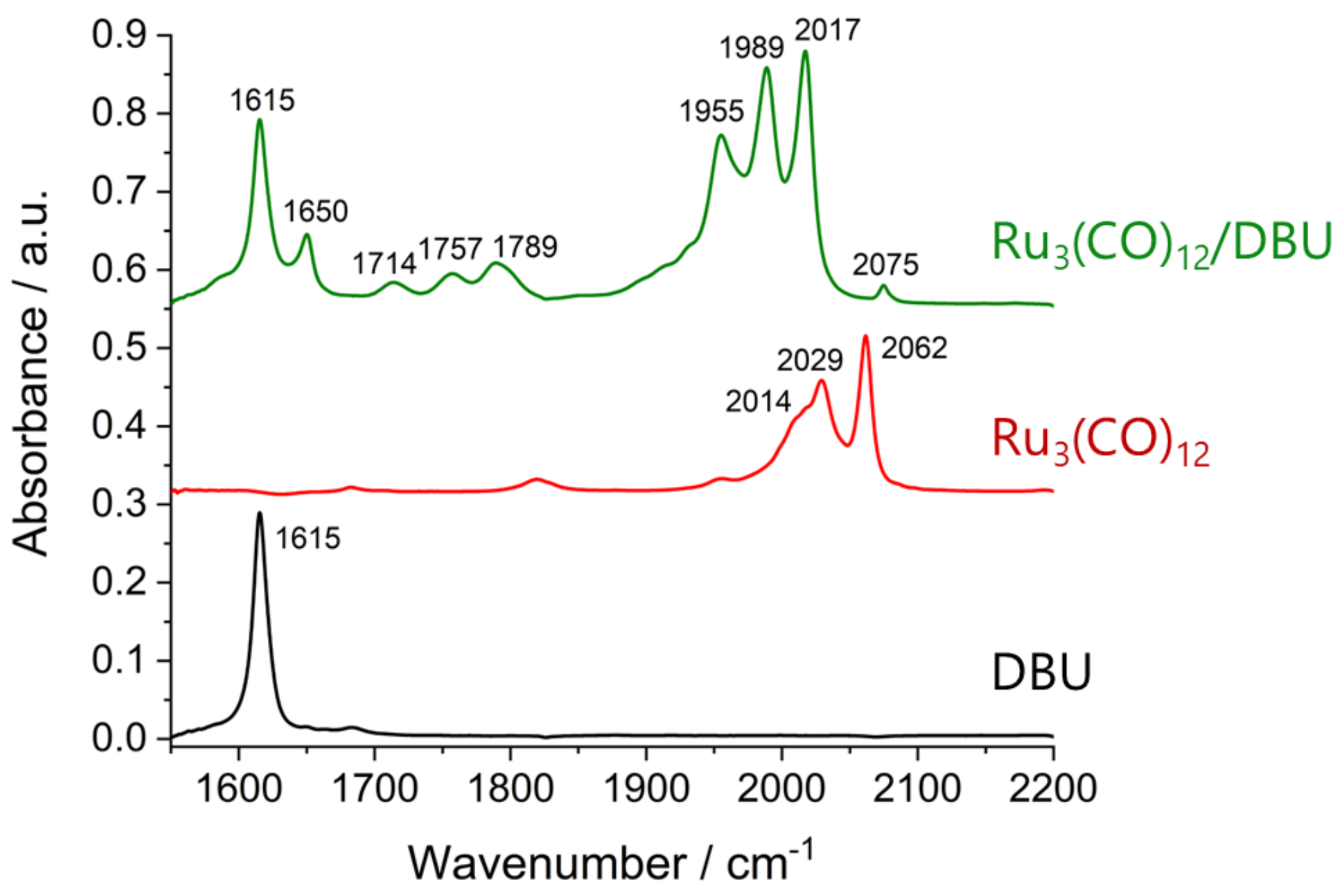

Figure S3 | Preliminary ex situ infrared spectra of liquid solution samples (DBU, $Ru_3(CO)_{12}$, $Ru_3(CO)_{12}$/DBU) prepared in acetonitrile under argon at room temperature

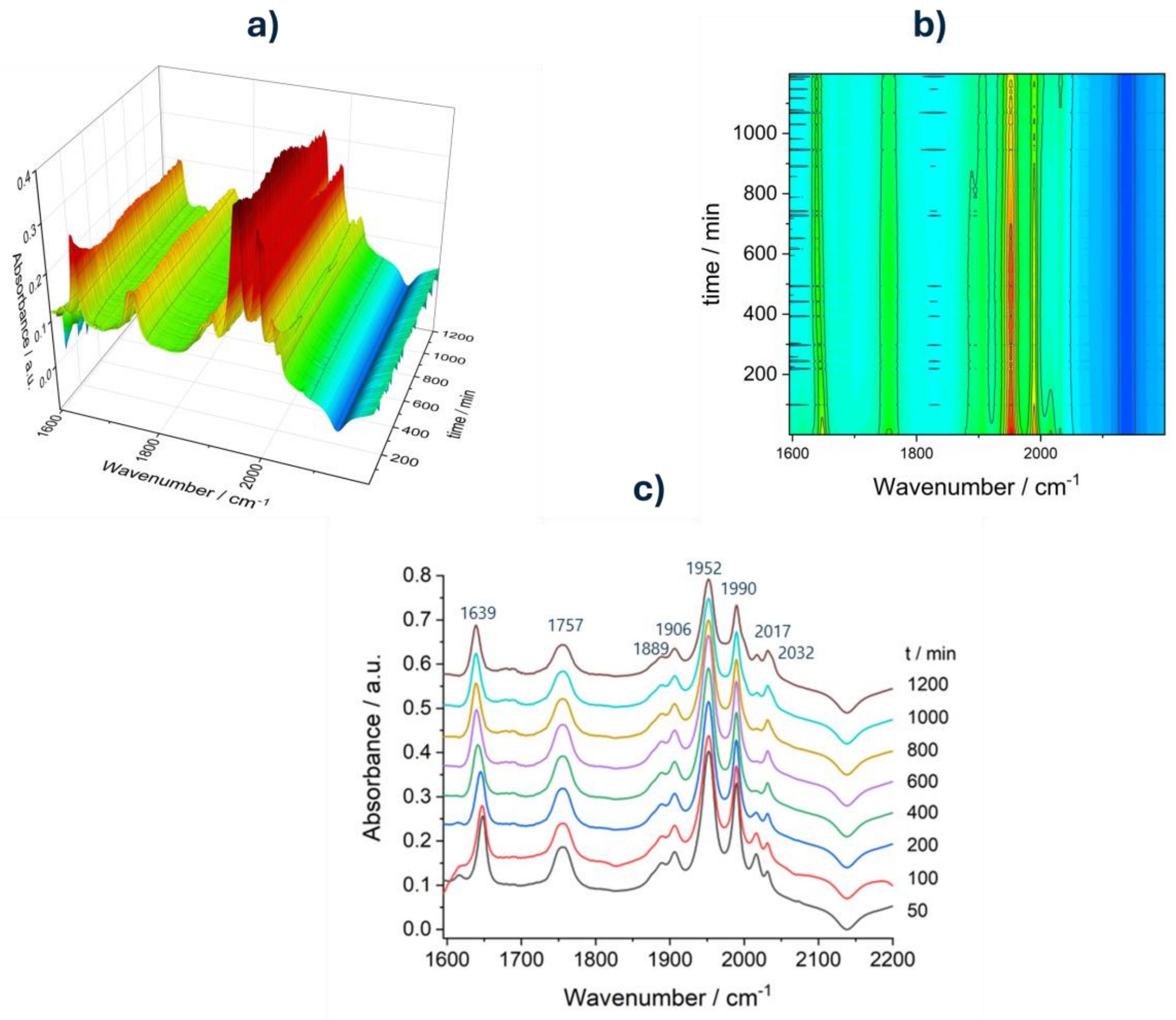

Figure S4 | In situ infrared spectroscopic data (3-D plot (a), contour plot (b), selected spectra as 2-D plot (c)) collected during the deuterium labelling experiment of [$Ru_3(CO)_{12}$]/DBU at reaction conditions for 1200 min: ϑ = 120 °C, p(D_2) = 35 bar, p(CO) = 5 bar

3.2. In situ HP-NMR

3.2.1 Pre-formation of [$Ru_3(CO)_{12}$] with DBU under H_2/CO

[$Ru_3(CO)_{12}$] (5.1 mg, 0.008 mmol) and DBU (4.9 µL, 0.032 mmol) were dissolved in dry CH_3CN (2 mL) by stirring under argon for 14 h at room temperature. 0.45 mL of the resulting red-brown solution were transferred to a 5 mm sapphire high pressure NMR cell under argon and pressurized with 5 bar CO and 35 bar H_2. After quick shaking the pressurized NMR cell was collocated in the spectrometer. The remaining reaction mixture was transferred to a 10 mL Swagelok 316 stainless steel reactor under argon,

pressurized with 5 bar CO and 35 bar H_2, heated at 120 °C and stirred 20 h for ex situ NMR measurements.

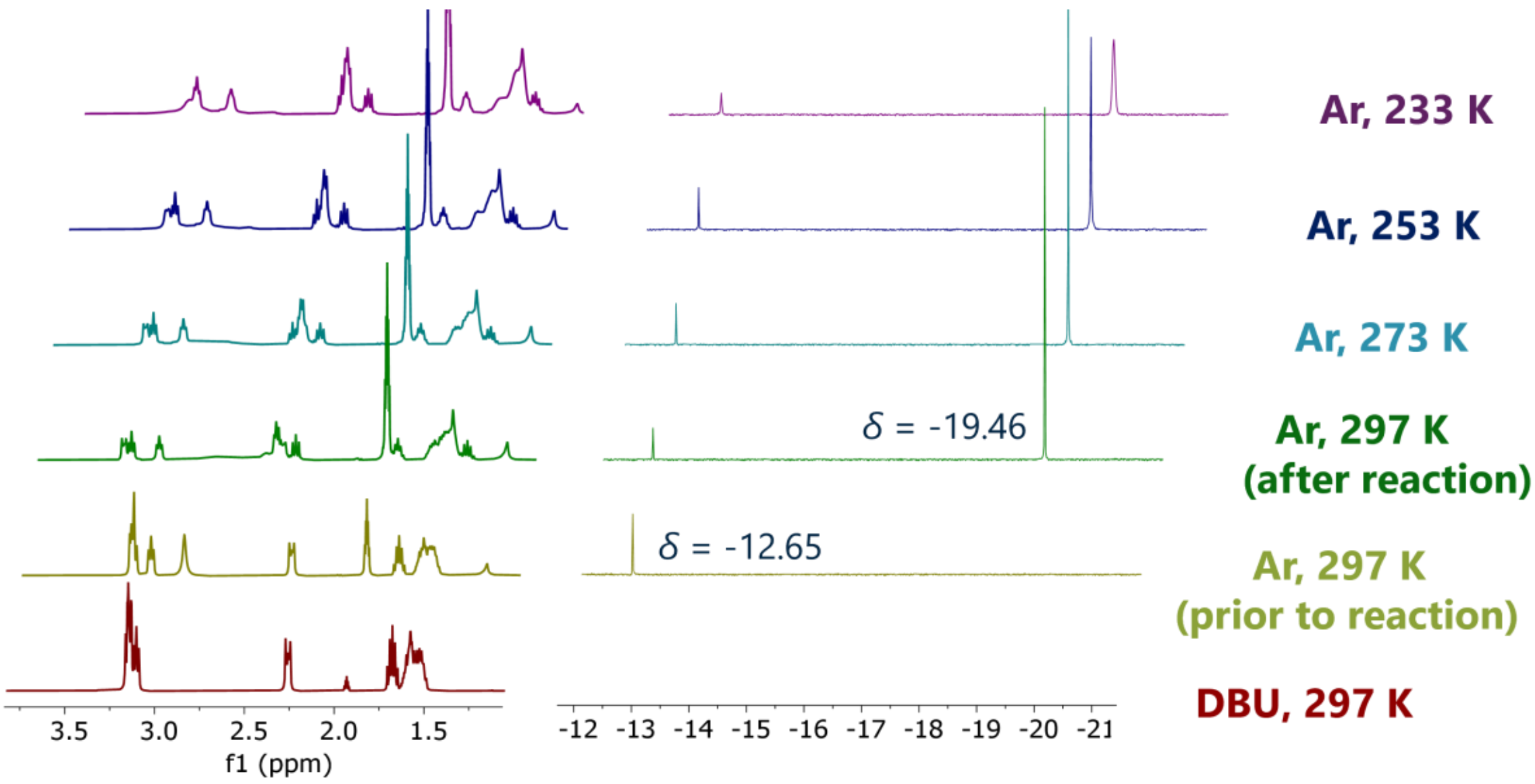

Figure S5 | Ex situ ^{1}H VT-NMR spectra after the reaction of [$Ru_3(CO)_{12}$] with DBU under argon. Reaction conditions: [Ru] = 12 mM, [DBU] = 15.6 mM (1.3 eq.), solvent = CD_3CN, ϑ = 393 K (120 °C), p(^{13}CO) = 5 bar, p(H_2)= 35 bar, reaction time = 15 h

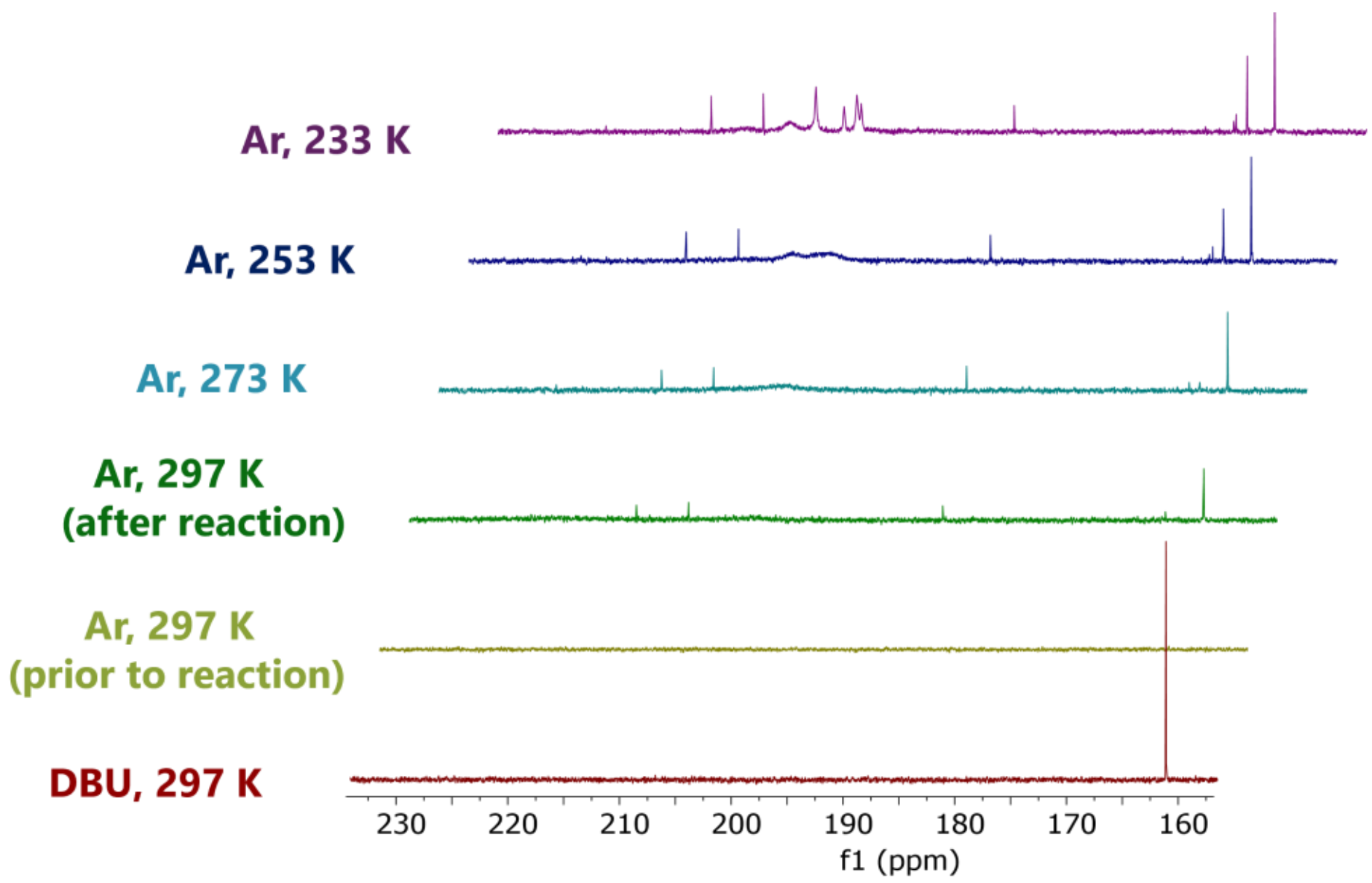

Figure S6 | Ex situ $^{13}C\{^1H\}$ VT-NMR spectra for the reaction of $[Ru_3(CO)_{12}]$ with DBU under argon. Reaction conditions: [Ru] = 12 mM, [DBU] = 15.6 mM (1.3 eq.), solvent = CD_3CN, ϑ = 393 K (120 °C), $p(^{13}CO)$ = 5 bar, $p(H_2)$= 35 bar, reaction time = 15 h

3.2.2. Hydroformylation of 3,3-Dimethylbut-1-ene (neohexene)

$[Ru_3(CO)_{12}]$ (6.4 mg, 0.01 mmol) and DBU (9.9 µL, 0.066 mmol) were dissolved in dry CH_3CN (2.5 mL) by stirring under argon for 14 h at room temperature. Then neohexene (0.2 mL, 1.55 mmol) was added and the mixture was stirred for 2 min. 0.45 mL of the resulting red-brown solution were transferred to a 5 mm sapphire high pressure NMR cell under argon and pressurized with 5 bar CO and 35 bar H_2. After quick shaking the pressurized NMR cell was collocated in the spectrometer. The rest of the reaction mixture was transferred to a 10 mL Swagelok 316 stainless steel reactor under argon, pressurized with 5 bar CO and 35 bar H_2, heated at 120 °C and stirred 22 h for ex situ NMR measurements.

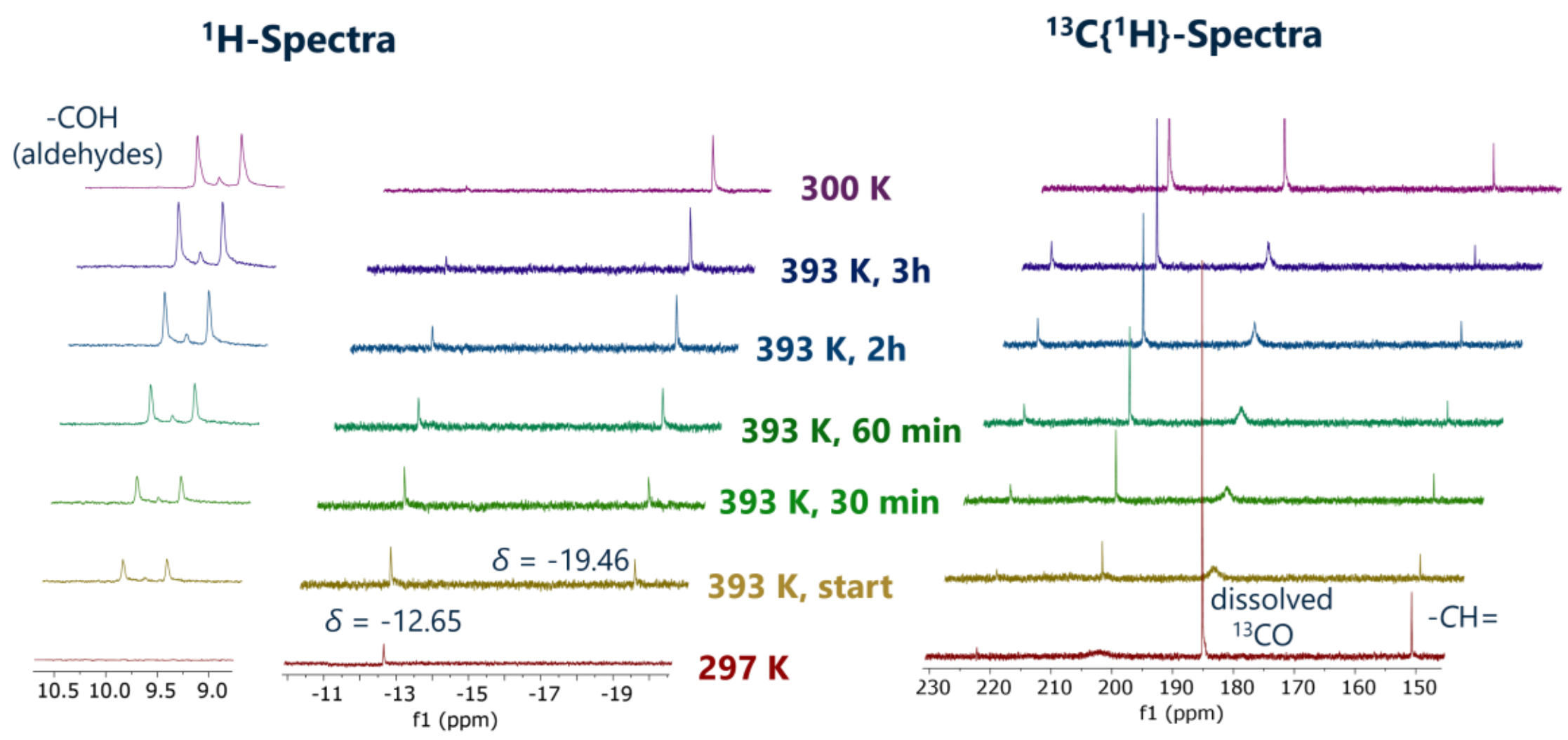

Figure S7 | In situ ^{1}H VT-HP NMR spectra for the hydroformylation of 3,3-Dimethylbut-1-ene with $[Ru_3(CO)_{12}]$ / DBU in a 5 mm sapphire NMR cell. Reaction conditions: [Ru] = 12 mM, [DBU] = 26.5 mM (2.2 eq.), [olefin] = 621 mM, solvent = CD_3CN, ϑ = 393 K (120 °C), p(^{13}CO) = 5 bar, p(H_2)= 35 bar, reaction time = 3 h

4. Isolation and characterization of the cluster anionic complex $[DBUH]_2^{+}[H_2Ru_4(CO)_{12}]^{2-}$

$[Ru_3(CO)_{12}]$ (63 mg, 0.099 mmol) and DBU (0.1 mL, 0.67 mmol) were dissolved in dry CH_3CN (25 mL) by stirring under argon for 14 h at room temperature. The resulting red-brown turbid solution was transferred to a 50 mL Swagelok 316 stainless steel reactor under argon, pressurized with 5 bar CO and 35 bar H_2, heated and stirred 48 h at 120 °C. After cooling down to room temperature, the pressure was released, and the resulting dark orange solution was transferred to a Schlenk-tube under argon. The solvent was removed under vacuum, and the residue was dissolved in dry degassed ethanol (0.9 mL). After adding dry degassed *n*-pentane (4.5 mL) an orange precipitated was formed. The solvent was extracted with a syringe, and the residue was washed with *n*-pentane (2 x 2 mL) and dried under vacuum. The orange solid was dissolved in dry degassed acetone (3 mL) and allowed to stand about 2 h at room temperature. A fine gray precipitate was formed, and the dark orange solution was extracted with a syringe and dried in vacuum to yield a dark orange solid (33.9 mg, 43%).

^{1}**H-NMR** (25 °C, CD_3CN, 400 MHz): δ(ppm) = 5.7 (bs, 2H, NH), 3.58–3.48 (m, 4H), 3.46 (t, *J* = 5.8 Hz, 4H), 3.27 (bs, 4H), 2.57 (bs, 4H), 2.70–2.53 (m, 4H), 1.99–1.95 (m, 4H), 1.71, 1.67 (2 x m, 12H), -19.47 (s, 2H).

^{13}**C-NMR** (25 °C, CD_3CN, 100.6 MHz): δ(ppm) = 221.6 (CO), 166.6, 54.8, 48.9, 38.8, 33.5, 29.0, 26.5, 23.9, 19.5.

^{1}**H-**^{15}**N HMBC** (25 °C, CD_3CN, 41 MHz): δ(ppm) = -258.5 (s), -267.6 (s).

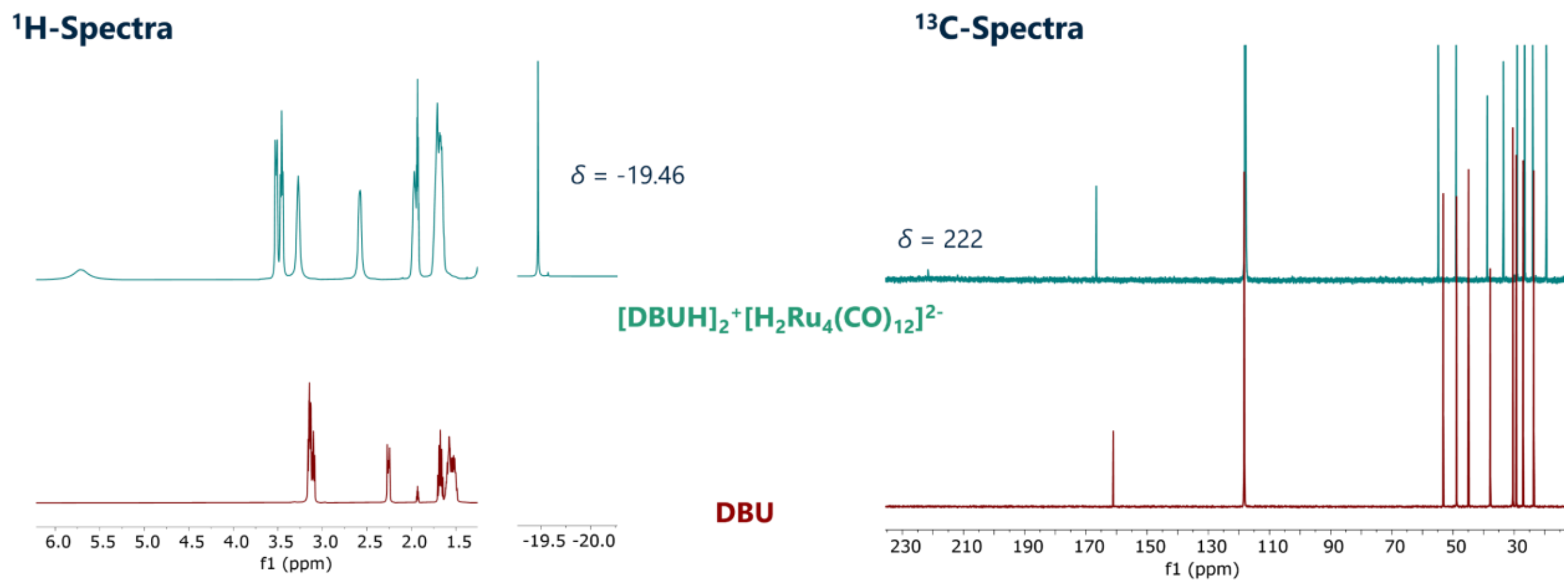

Figure S8 | Comparison of ^{1}H- and ^{13}C-NMR spectra of DBU and $[DBUH]_2^+[H_2Ru_4(CO)_{12}]^{2-}$ in acetonitrile-*d*$_3$

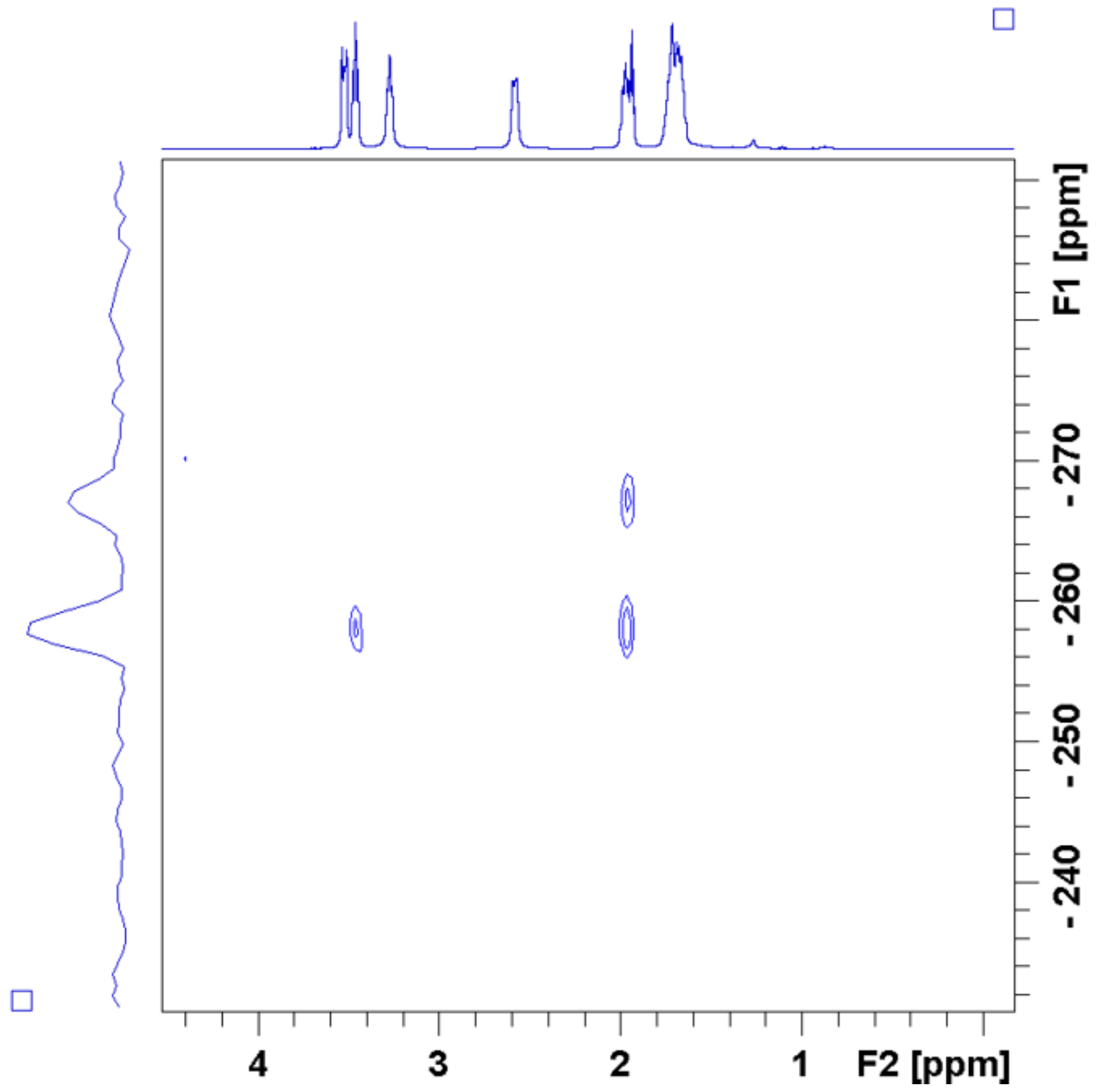

Figure S9 | ^{1}H-^{15}N HMBC NMR spectrum of $[DBUH]_2^{+}[H_2Ru_4(CO)_{12}]^{2-}$ in acetonitrile-*d*$_3$

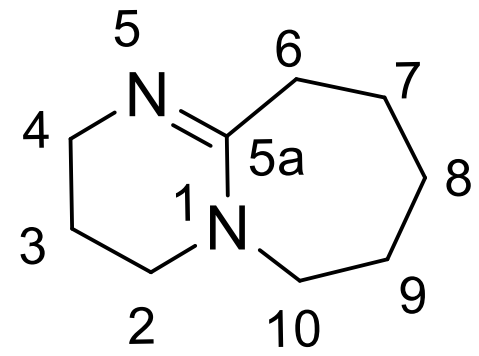

Table S15 | Comparative ^{1}H-NMR data for DBU and DBUH^{+}

Wiench *et al.*[73]			This work		
δ **(DBU), ppm**	***δ*** **(DBUH^{+}), ppm**	**Δ*δ***	***δ*** **(DBU), ppm**	***δ*** **(DBUH^{+})^{a}, ppm**	**Δ*δ***
3.19 (**H2**)	3.39	0.2	3.16	3.47	0.31
1.70 (**H3**)	1.91	0.21	1.68	1.97	0.29

3.12 (**H4**)	3.24	0.12	3.10	3.27	0.17
2.29 (**H6**)	2.61	0.32	2.26	2.58	0.32
1.60 (**H7**)	1.68	0.08	1.58	1.69	0.11
1.62 (**H8**)	1.73	0.11	1.60	1.71	0.11
1.54 (**H9**)	1.66	0.12	1.52	1.67	0.15
3.17 (**H10**)	3.44	0.27	3.14	3.52	0.38
- (**5NH**)	8.19	–	–	5.71	–

[a] corresponds to the chemical shift of the cation from isolated complex $[DBUH]_2^+[H_2Ru_4(CO)_{12}]^{2-}$. Δ***δ*** (in ppm) means the difference: ***δ*** ($DBUH^+$) – ***δ*** (DBU).

Table S16 | Comparative ^{13}C- and ^{15}N-NMR data for DBU and DBUH$^+$

**Wiench *et al.*[73]**			**This work**		
δ **(DBU), ppm**	***δ*** **($DBUH^+$), ppm**	**Δ*δ***	***δ*** **(DBU), ppm**	***δ*** **($DBUH^+$)[a], ppm**	**Δ*δ***

-290.0 (**N1**)	-263.4	26.6	-286.4	-267.6	18.8
48.2 (**C2**)	49.2	1.0	48.8	48.9	0.1
22.3 (**C3**)	20.8	-1.5	23.6	19.5	-4.1
43.9 (**C4**)	39.9	-4.0	44.9	38.8	-6.1
-173.0 (**N5**)	-260.6	-87.6	-183.1	-258.5	-75.4
161.5 (**C5a**)	166.2	4.7	161.1	166.6	5.5
37.0 (**C6**)	33.8	-3.2	37.8	33.5	-4.3
25.8 (**C7**)	25.1	-0.7	27.0	23.9	-3.1
29.6 (**C8**)	29.8	0.2	30.4	29.0	-1.4
28.3 (**C9**)	27.6	-0.7	29.3	26.5	-2.8
52.7 (**C10**)	54.6	1.9	53.2	54.8	1.6

[a] corresponds to the chemical shift of the cation from isolated complex $[DBUH]_2^+[H_2Ru_4(CO)_{12}]^{2-}$. Δ***δ*** (in ppm) means the difference: ***δ*** ($DBUH^+$) – ***δ*** (DBU.

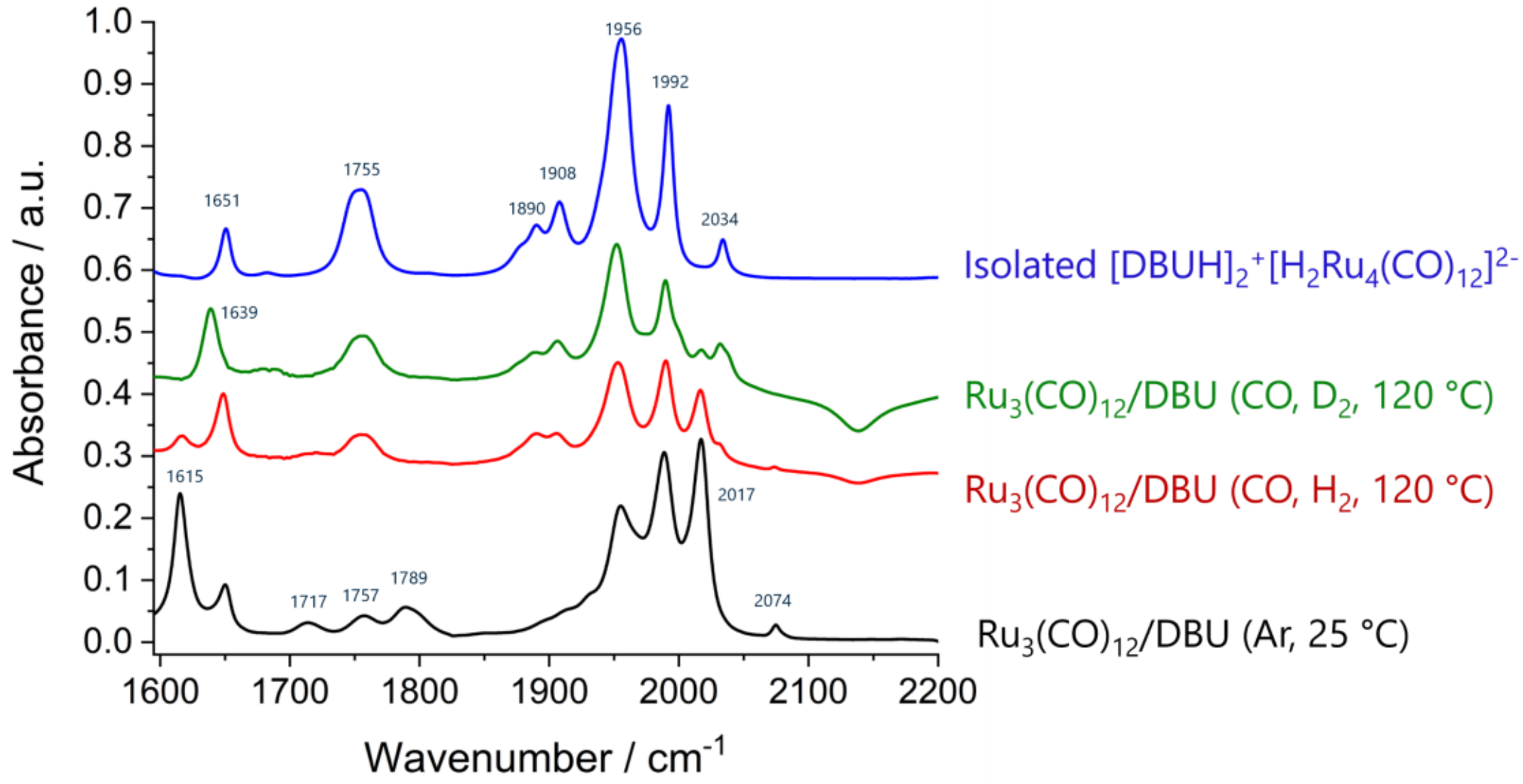

Figure S10 | Comparison of the infrared spectrum of isolated complex $[DBUH]_2^+[H_2Ru_4(CO)_{12}]^{2-}$ in acetonitrile-d_3 with the mixture $[Ru_3(CO)_{12}]$ / DBU under different conditions

5. NMR Spectra

5.1. tridecanal (2b) and 2-methyldodecanal (2b')

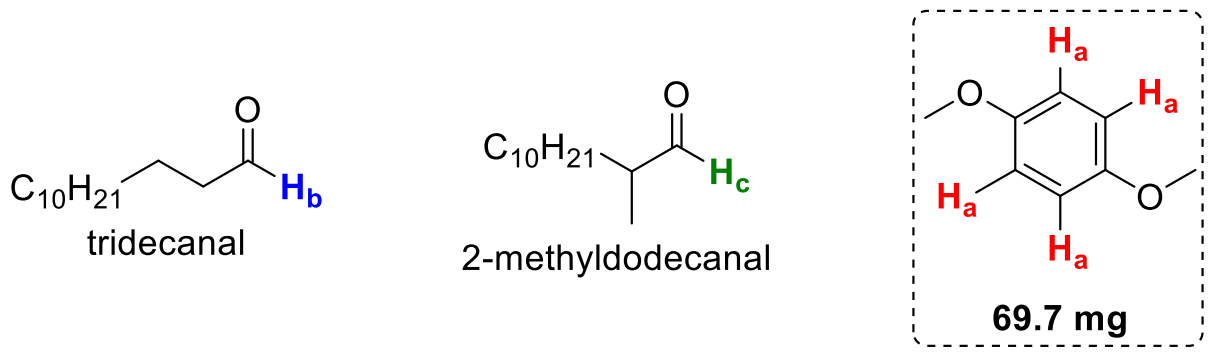

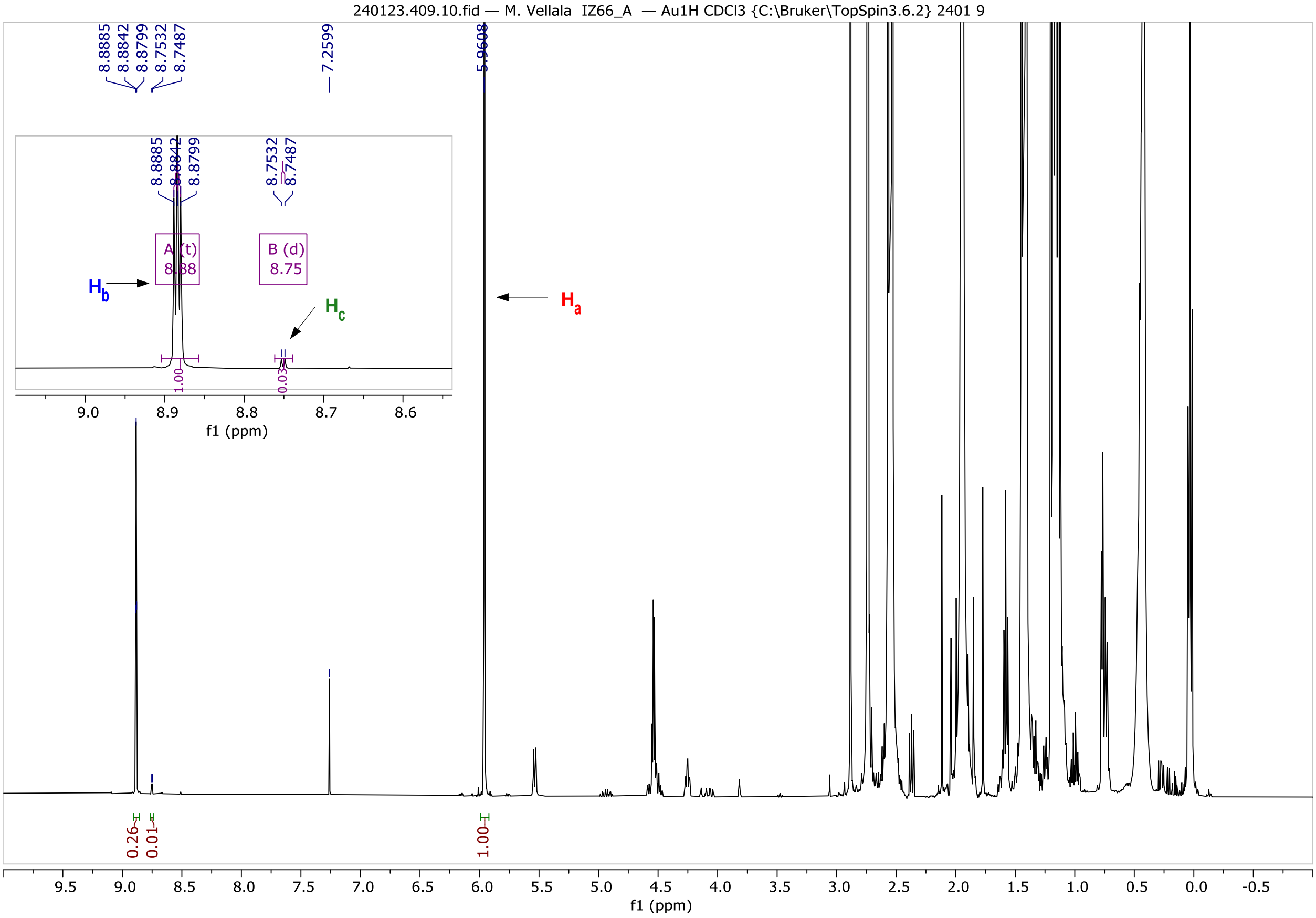

5.2. heptanal (2c) and 2-methylhexanal (2c')

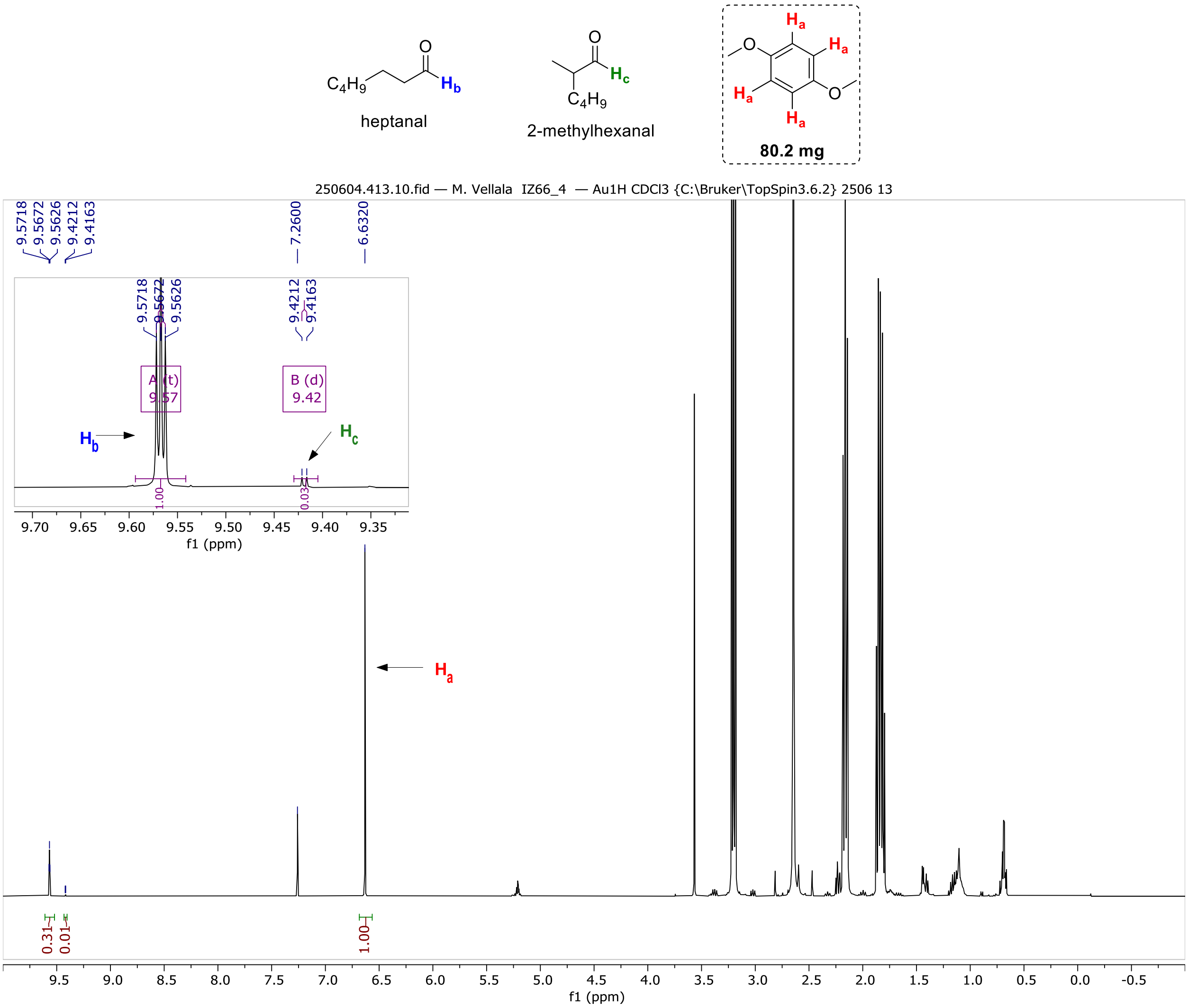

5.3. butyraldehyde (2d) and isobutyraldehyde (2d')

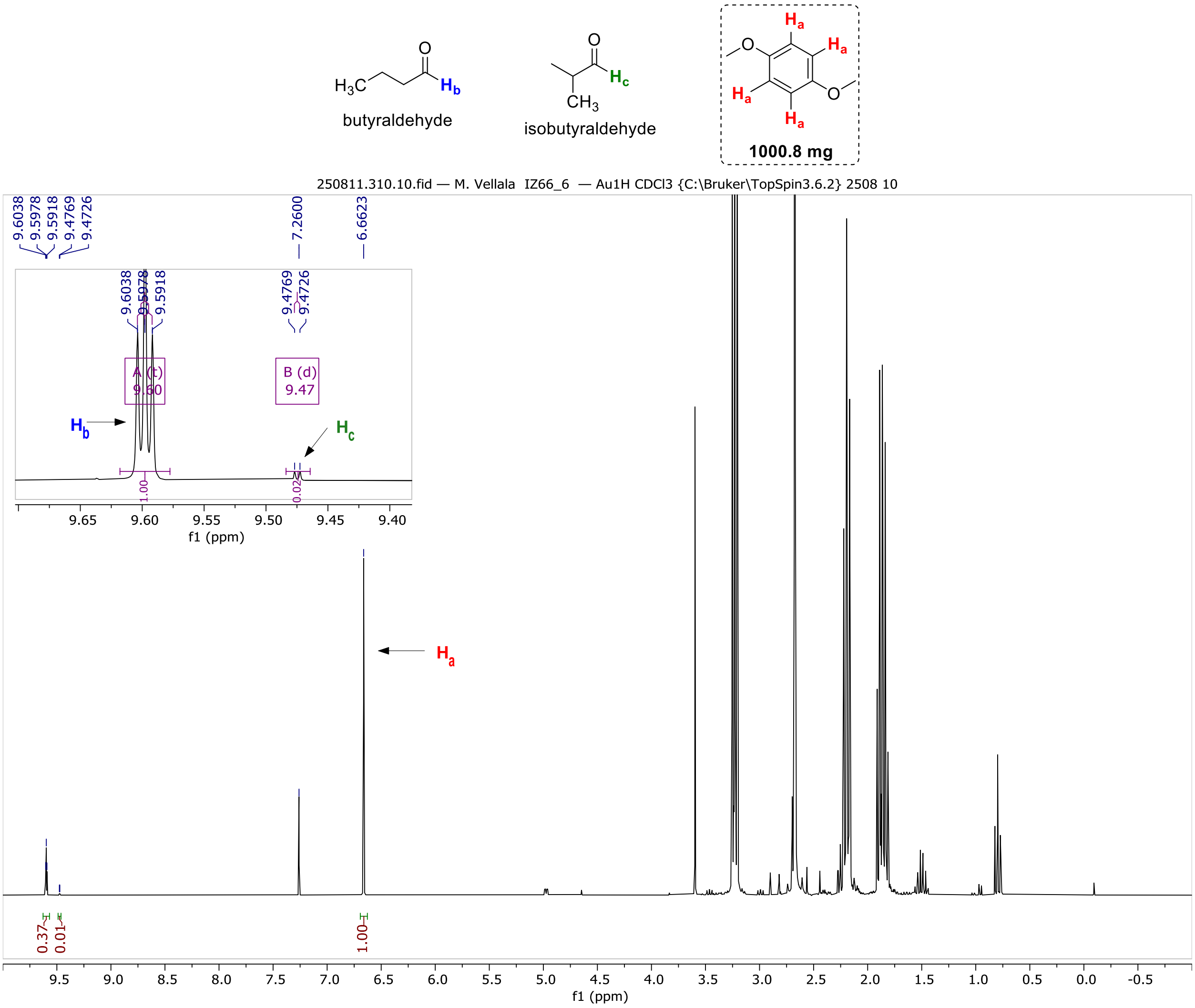

5.4. 3,5,5-trimethylhexanal (2e) and 2,2,4,4-tetramethylpentanal (2e')

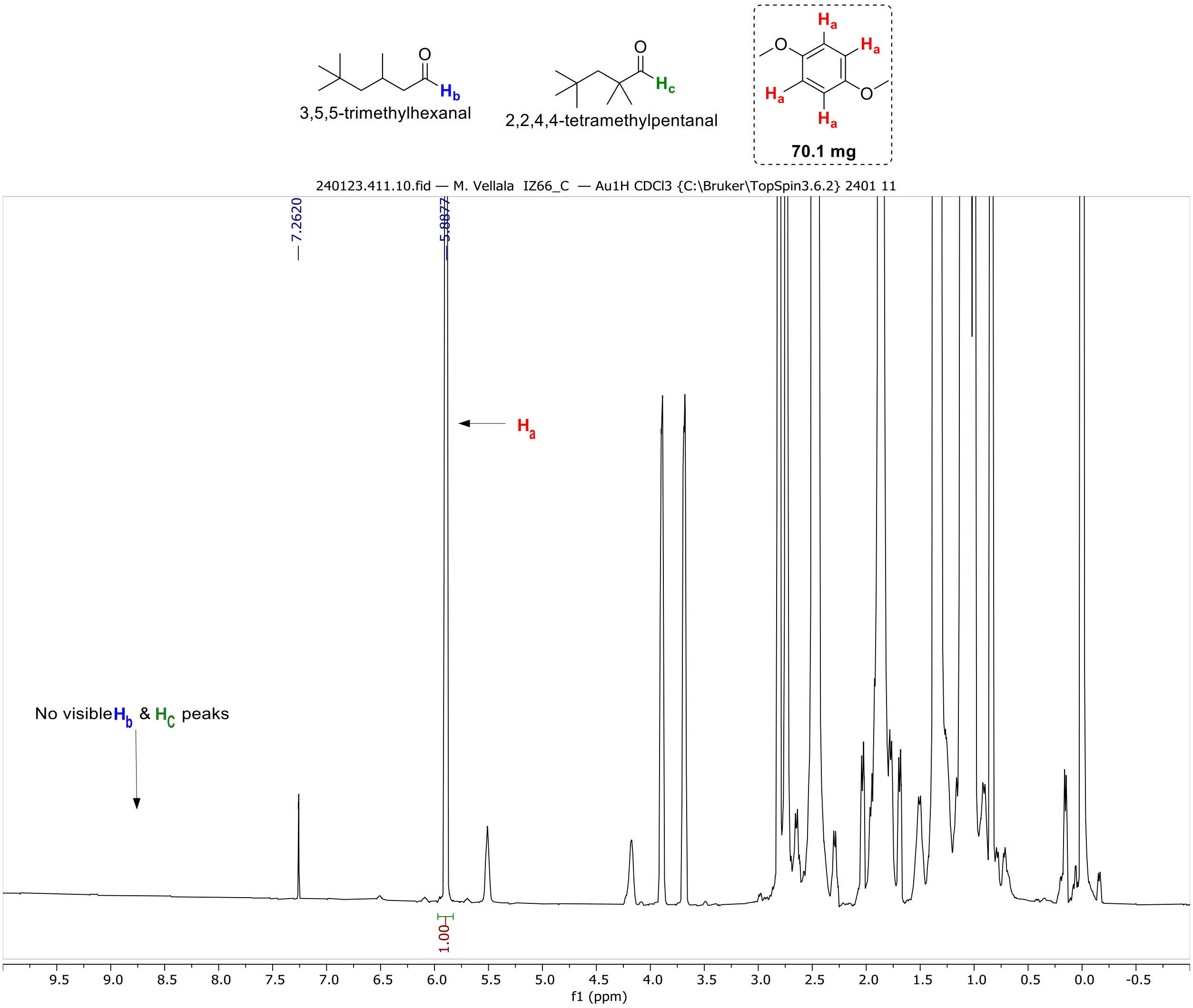

5.5. 4,4-dimethylpentanal (2f) and 2,3,3-trimethylbutanal (2f')

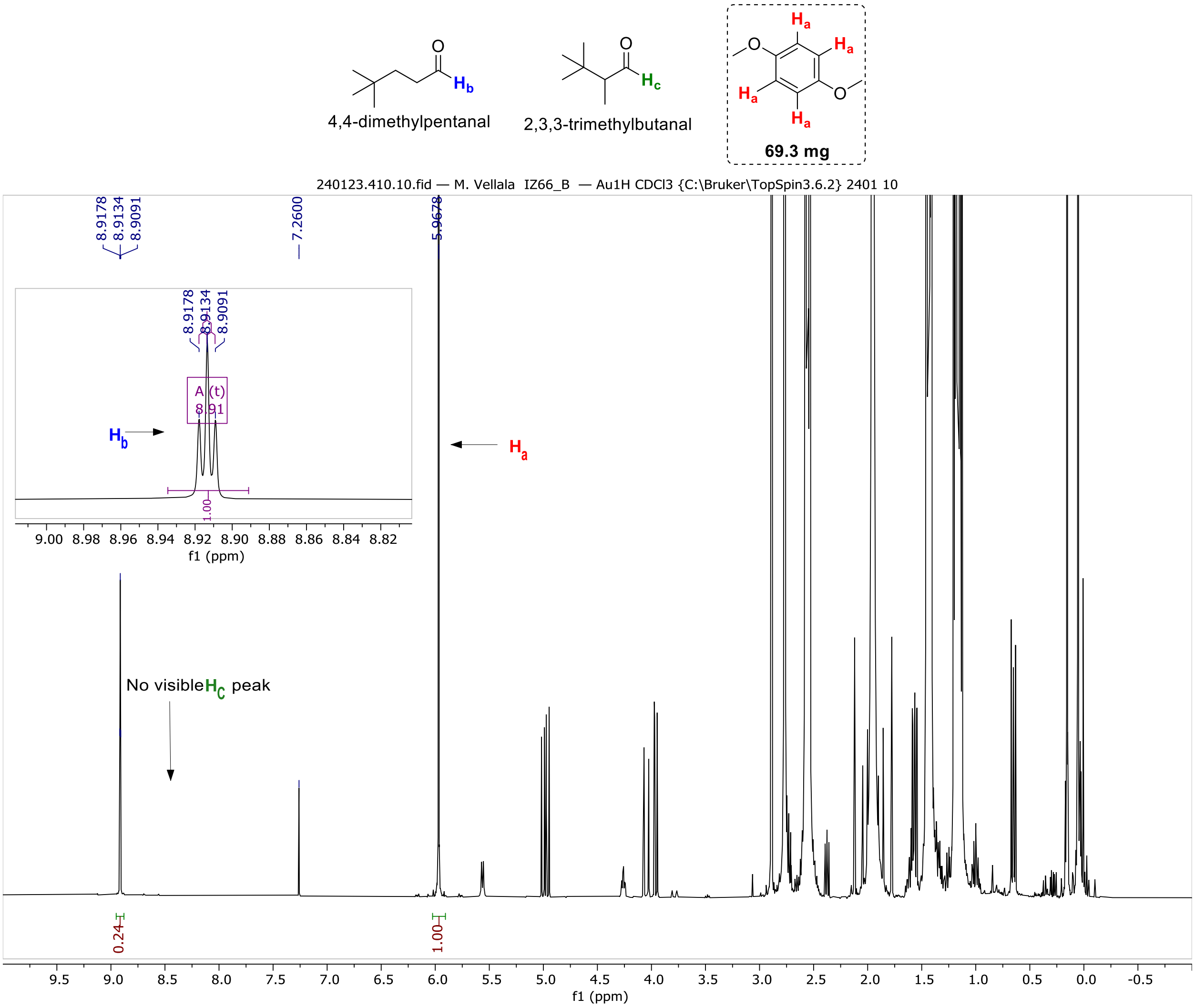

5.6. 3-phenylpropanal (2g) and 2-phenylpropanal (2g')

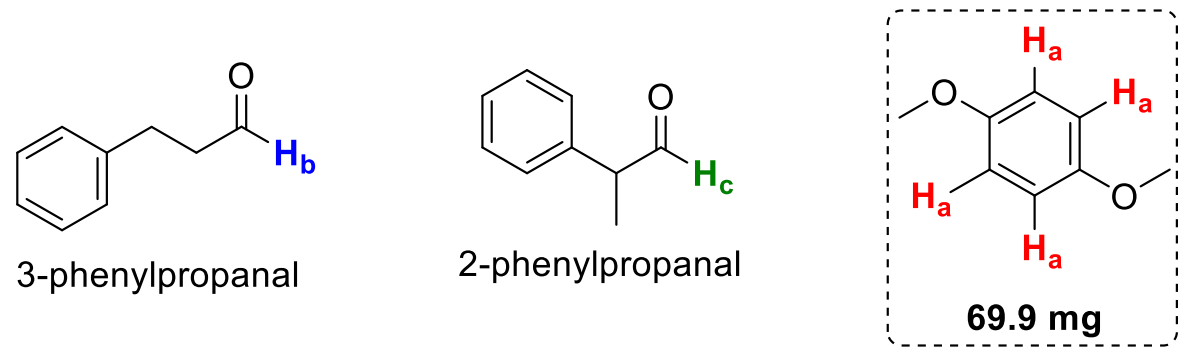

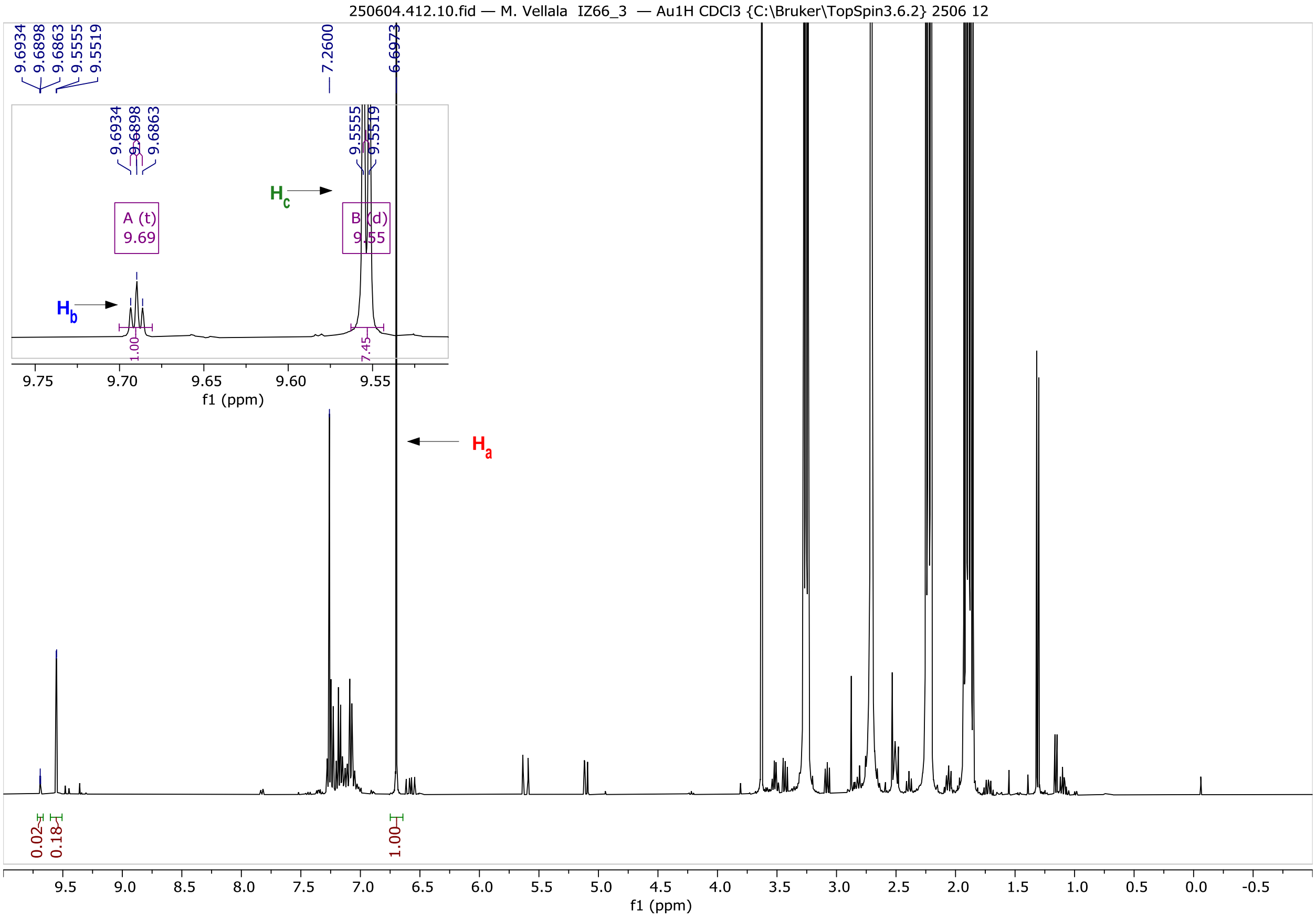

5.7. 3-(4-methoxyphenyl)propanal (2h) and 2-(4-methoxyphenyl)propanal (2h')

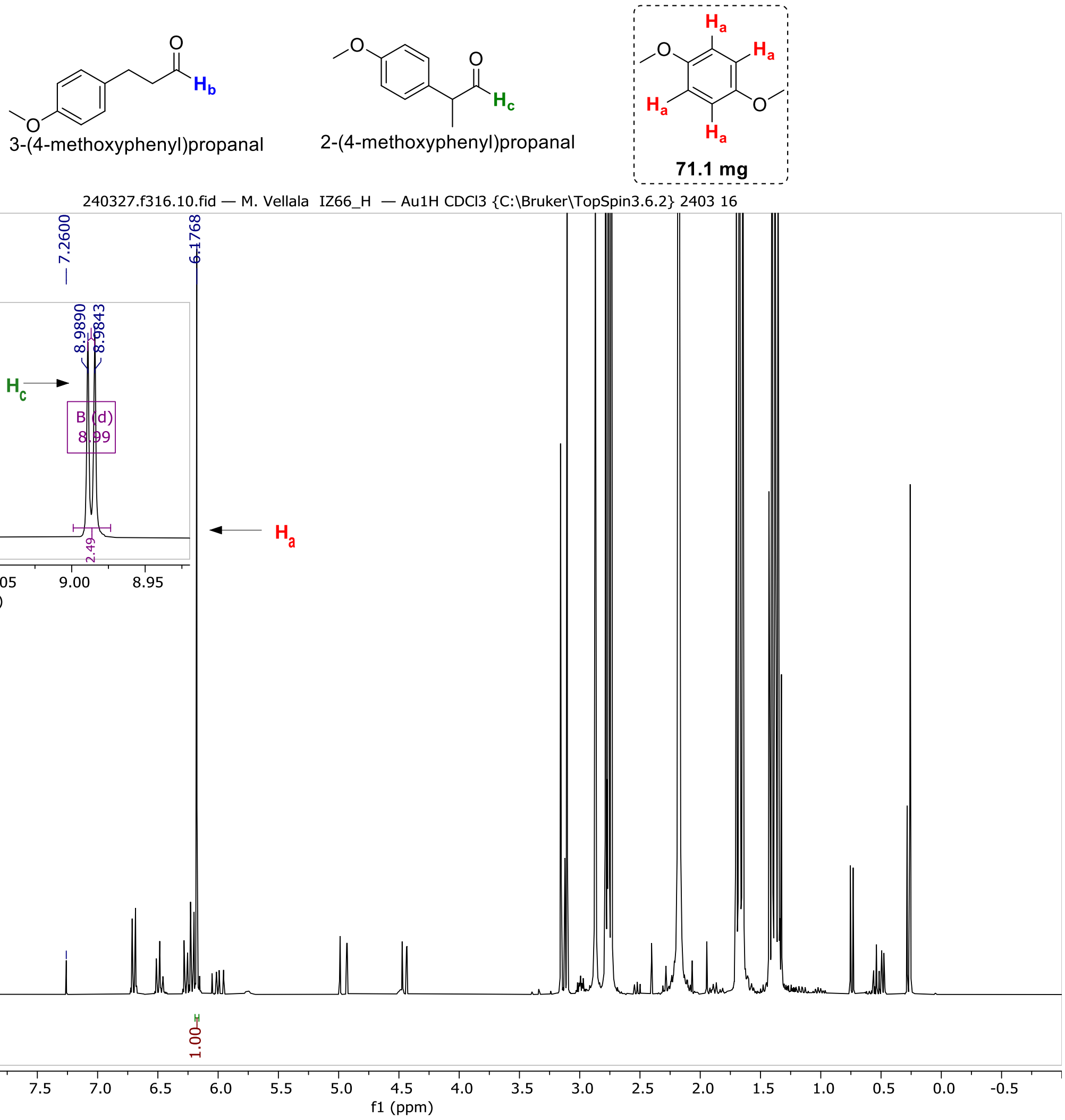

5.8. 3-(*p*-tolyl)propanal (2i) and 2-(*p*-tolyl)propanal (2i')

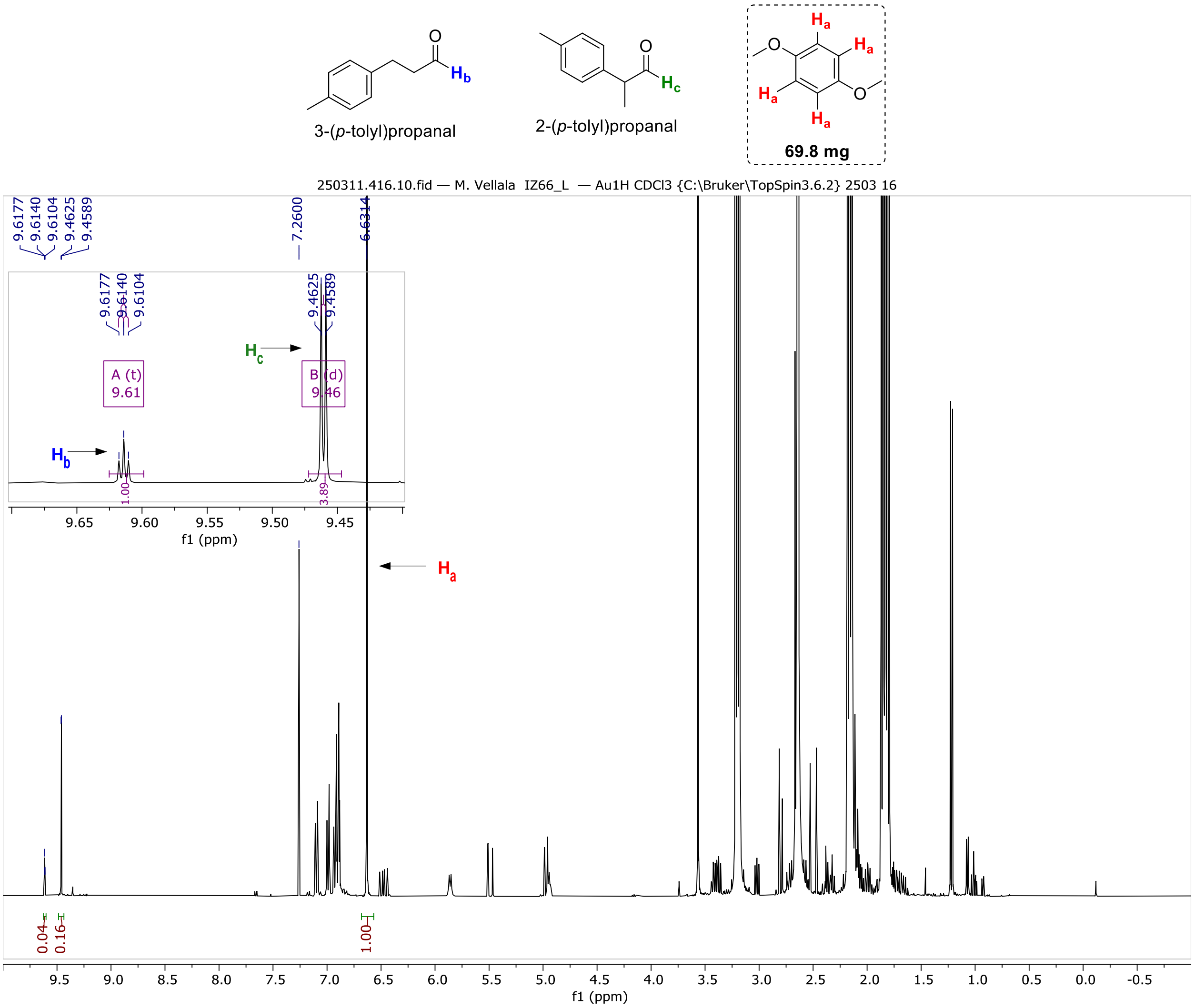

5.9. 3-(4-nitrophenyl)propanal (2j) and 2-(4-nitrophenyl)propanal (2j')

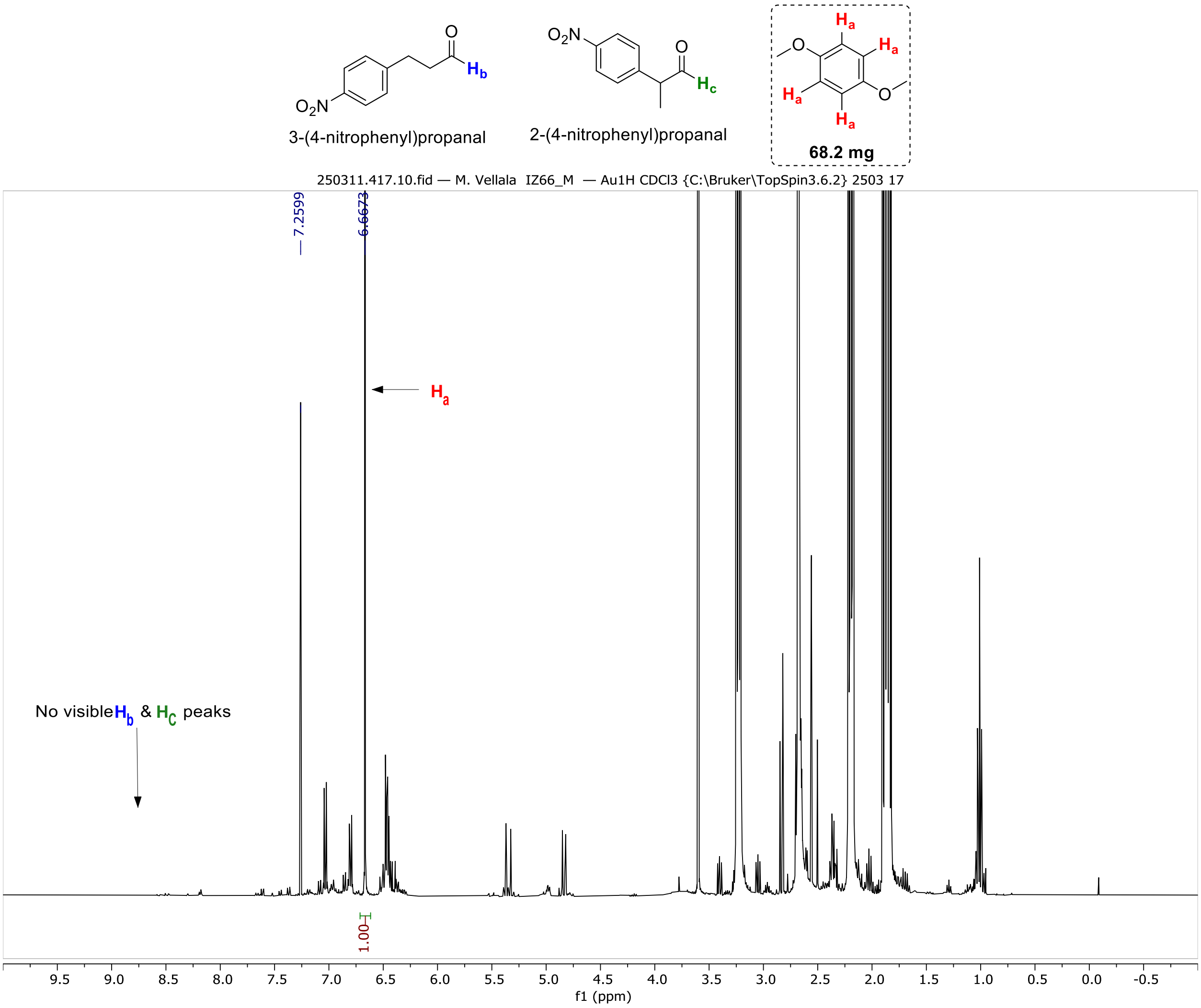

5.10. 3-(4-(trifluoromethyl)phenyl)propanal (2k) and 2-(4-(trifluoromethyl)phenyl)propanal (2k')

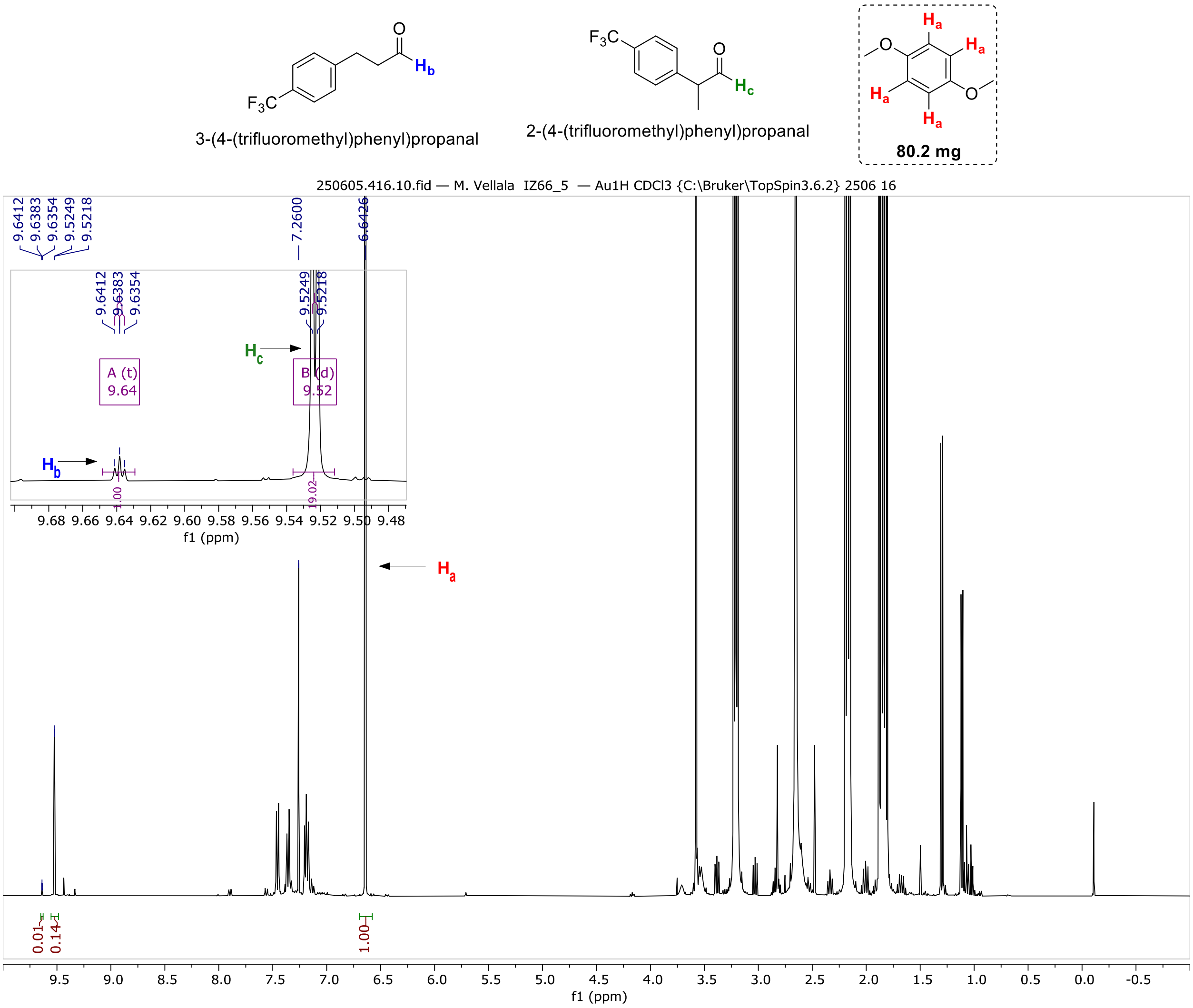

5.11. 3-(1,3-dioxoisoindolin-2-yl)propanal (2l) and 2-(1,3-dioxoisoindolin-2-yl)propanal (2l')

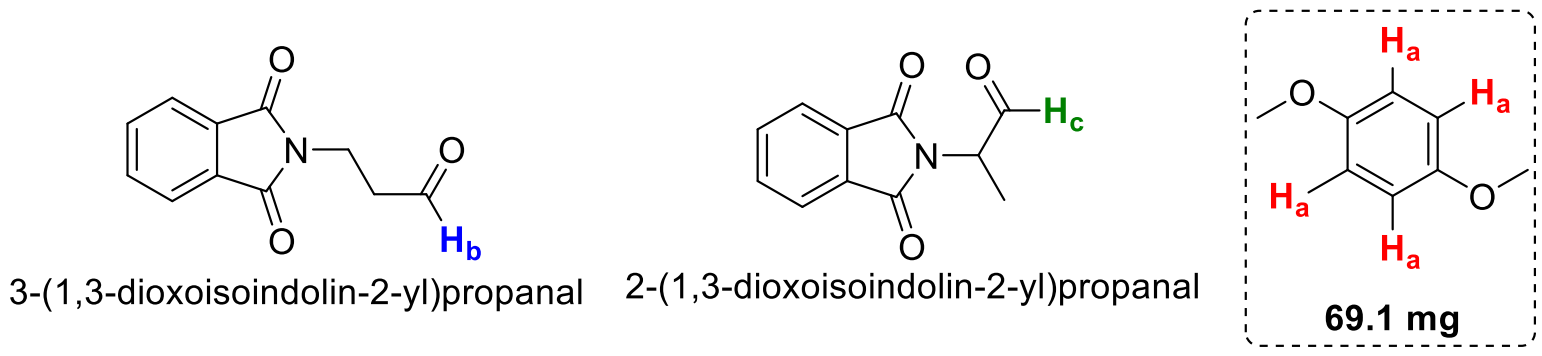

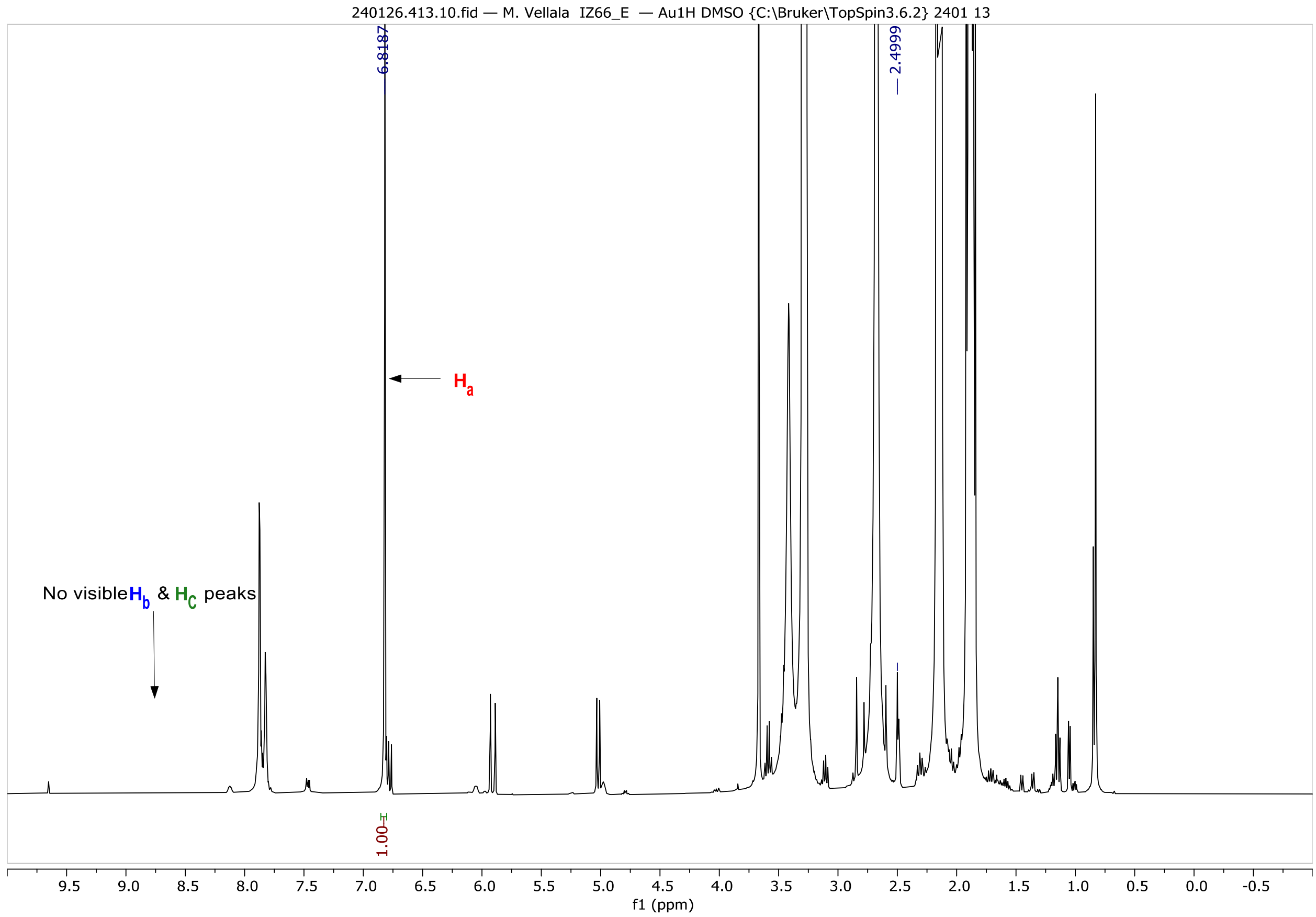

5.12. 3-(cyclohex-3-en-1-yl)propanal (2m) and 2-(cyclohex-3-en-1-yl)propanal (2m')

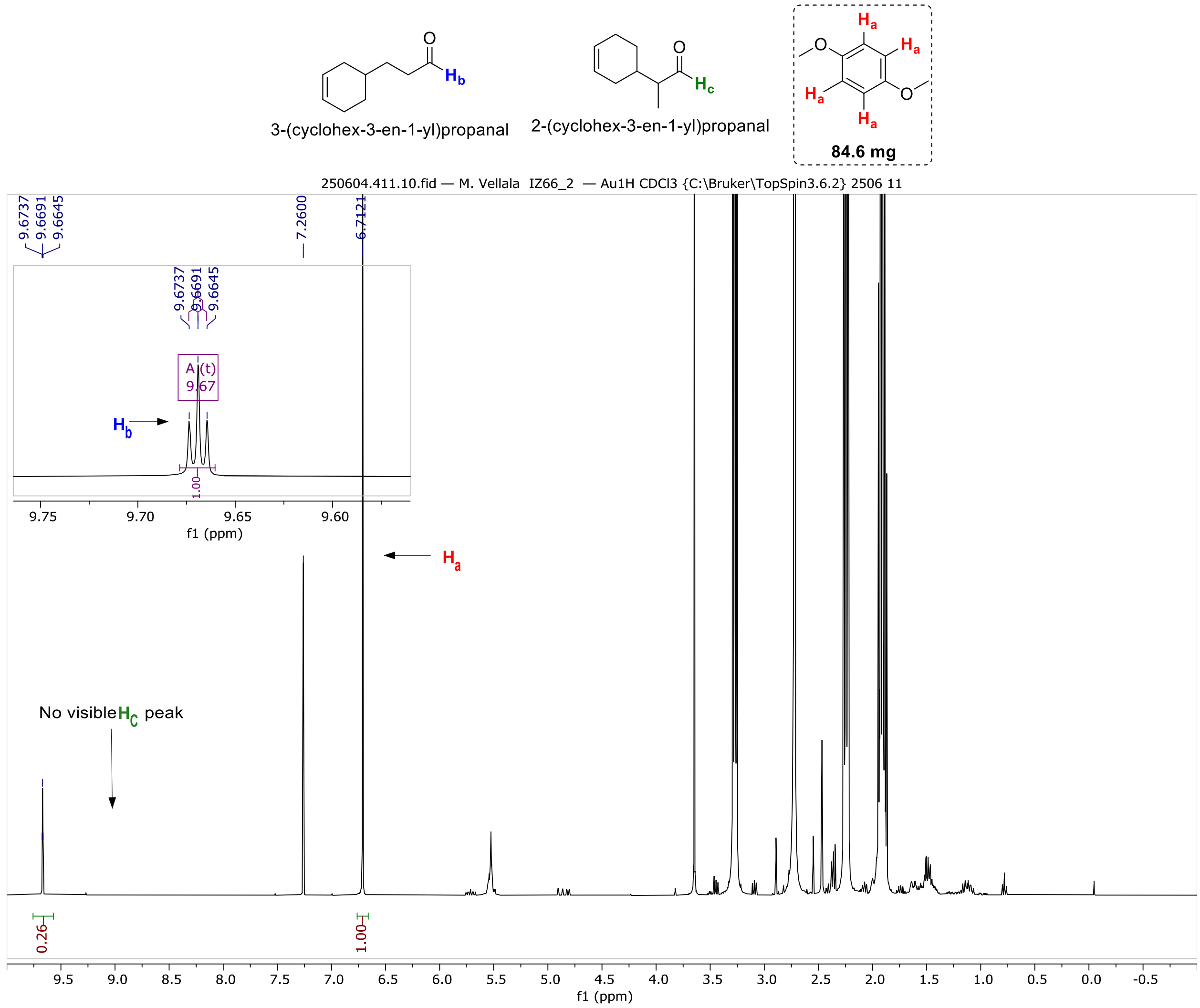

5.13. 3-(1,3-dioxolan-2-yl)propanal (2n) and 2-(1,3-dioxolan-2-yl)propanal (2n’)

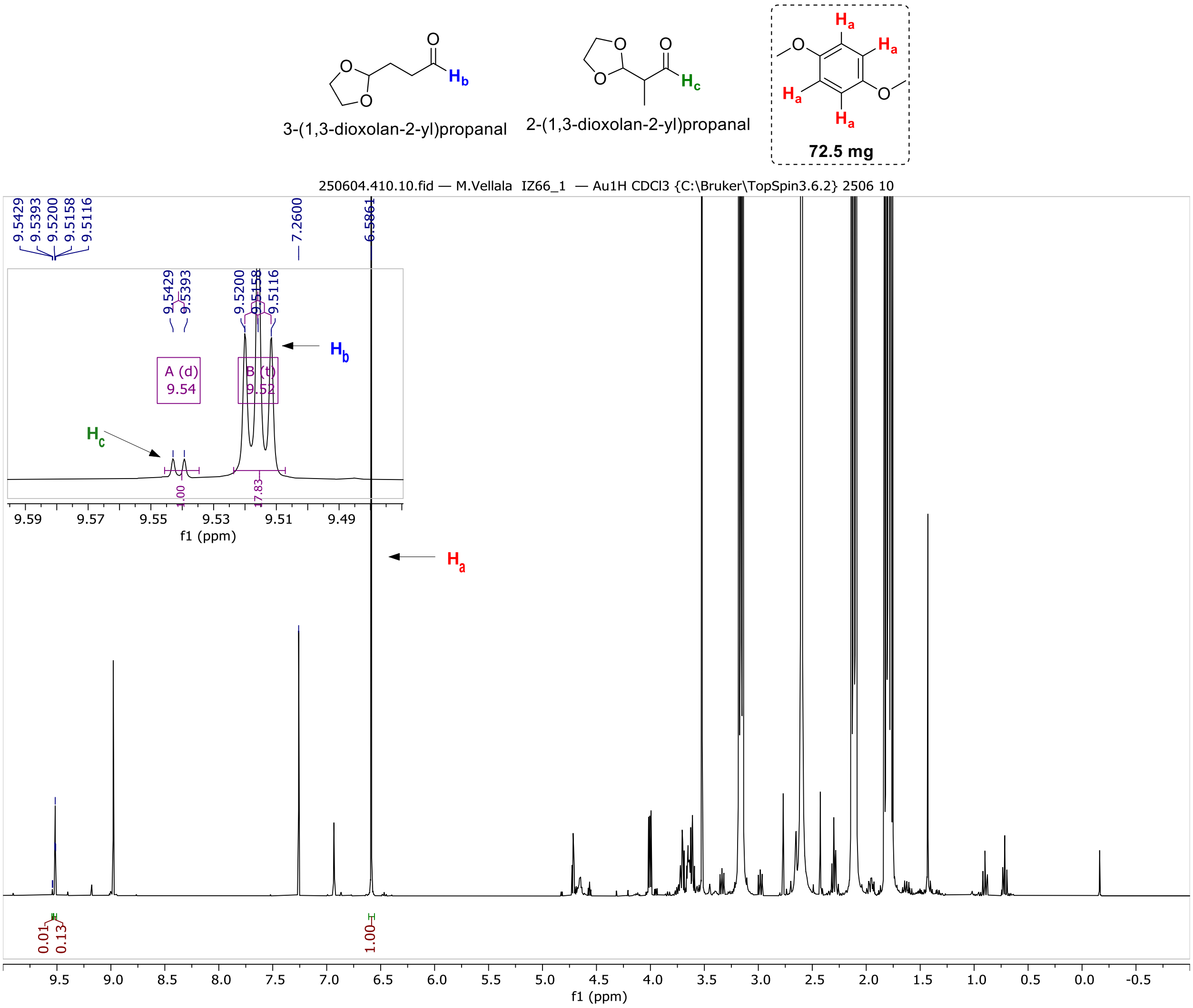

6. Supplementary references

[71] Purification of Laboratory Chemical (Eds: Perrin, D. D.; Armarego, W. L. F.-). Pergamon Press, Oxford, **1988**, *3*.

[72] R. K. Harris, E. D. Becker, S. M. Cabral de Menezes, P. Granger, R. E. Hoffman, K. W. Zilm, *Pure Appl. Chem.* **2008**, *80*, 59-84.

[73] J. W. Wiench, L. Stefaniak, E. Grech, E. Bednarek, *J. Chem. Soc., Perkin Trans. 2* **1999**, 885-890.
